# Synthesis of 1,3-Thiazine and 1,4-Thiazepine Derivatives via Cycloadditions and Ring Expansion

**DOI:** 10.3390/ijms262311543

**Published:** 2025-11-28

**Authors:** Márta Palkó, Nóra Becker, Edit Wéber, Matti Haukka, Attila Márió Remete

**Affiliations:** 1 Institute of Pharmaceutical Chemistry, University of Szeged, Eötvös u. 6, H-6720 Szeged, Hungary; palko.marta@szte.hu (M.P.); beckernori@gmail.com (N.B.); 2 Department of Medical Chemistry, University of Szeged, Dóm tér 8, H-6720 Szeged, Hungary; weber.edit@med.u-szeged.hu; 3 HUN-REN-SZTE Biomimetic Systems Research Group, Dóm tér 8, H-6720 Szeged, Hungary; 4 Department of Chemistry, University of Jyväskylä, FIN-40014 Jyväskylä, Finland; matti.o.haukka@jyu.fi

**Keywords:** alkenes, cycloaddition, hetero-Diels–Alder reaction, β-lactams, 1,4-thiazepines, 1,3-thiazines

## Abstract

Non-cephem drugs with 1,3-thiazine-derived rings are very rare, although a number of bioactive 1,3-thiazine derivatives are known. Similarly, 1,4-thiazepine-derived drugs are rare, but many 1,4-thiazepine derivatives show interesting biological activities. Therefore, our aim was the synthesis of such *N*,*S*-heterocycles using a versatile and short (1–3 steps) literature method. First, a three-component reaction of a cycloalkene, a thioamide, and an aldehyde provided 5,6-dihydro-4*H*-1,3-thiazines. Afterwards, Staudinger ketene–imine cycloaddition with chloroketene resulted in β-lactam-fused 1,3-thiazinanes. Finally, treatment with sodium methoxide induced ring expansion, yielding 4,5,6,7-tetrahydro-1,4-thiazepines. This synthetic pathway generates 3–5 new chiral centers with the help of pericyclic reactions, and almost every cycloaddition proceeded in a diastereoselective manner. Two-dimensional NOESY as well as single-crystal X-ray diffraction enabled unequivocal determination of the stereochemistry of all synthesized compounds.

## 1. Introduction

Azaheterocycles show outstanding structural diversity (azaheterocycles with various ring sizes, heteroatom patterns, substituent patterns, and saturation are readily available synthetically [1]), are widespread in nature (nucleobases [1], many B vitamins [1], numerous alkaloids [2], etc.), and in drugs [3,4,5]. Incorporation of an *N*-heterocycle into a molecule has considerable influence on important pharmaceutical parameters such as hydrogen bond donor/acceptor properties, lipophilicity, and solubility in water [5].

**Figure 1:**
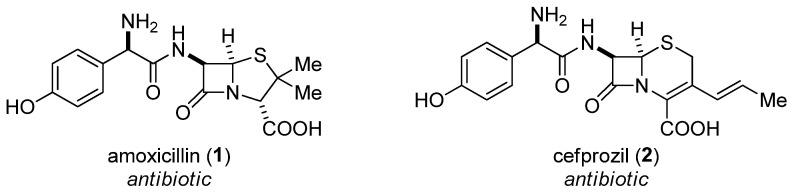
Examples of β-lactam drugs.

Within azaheterocycles, β-lactams are an important subfamily. Many important antibiotics (e.g., penicillins, cephalosporins, and carbapenems) contain a β-lactam ring, which has a key role in their bioactivity [6]. A number of β-lactamase inhibitors (drugs used to counter antibiotic resistance) [6] and the cholesterol-lowering drug ezetimibe [7] are also β-lactam derivatives. Some important β-lactam drugs are depicted in Figure 1.

**Figure 2:**
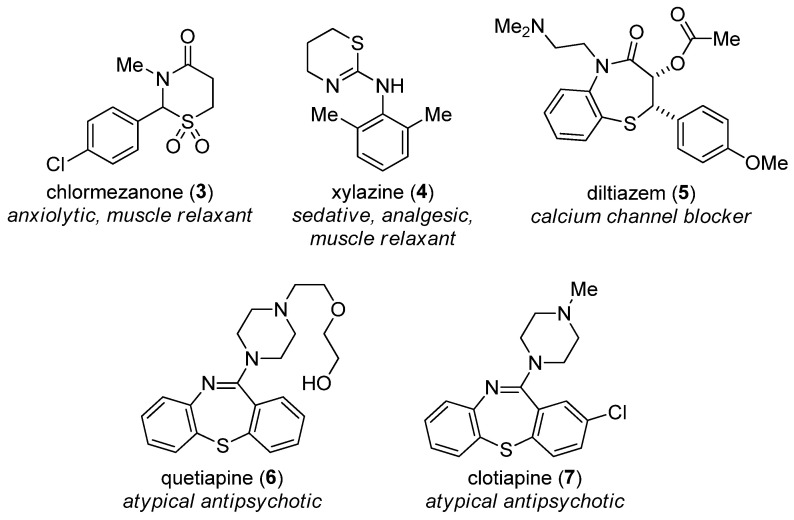
Drugs containing 1,3-thiazine- or 1,4-thiazepine-derived rings.

Heterocycles with both nitrogen and sulfur atoms in the ring are also found in pharmaceuticals. The most common related heterocycles are (benzo)thiazoles, penicillins (β-lactam-fused thiazolidine core, see **1** as an example) [3,4,8,9]. Further examples are (benzo)thiophenes, phenothiazines, and cephems (β-lactam-fused dihydro-1,3-thiazine core, see **2** as an example) [3,4,8,9]. It caught our attention that while cephems are relatively common, other drugs with 1,3-thiazine-derived rings (Figure 2) are very rare [8,9,10], despite the fact that a number of 1,3-thiazine derivatives show interesting biological activities [11,12,13,14,15]. Interestingly, 1,4-thiazepine-derived ring systems show a similar tendency. Such motifs are rare in drugs (Figure 2) [8,9], although they are present in many bioactive compounds [16,17,18,19,20,21,22,23,24,25,26,27,28].

**Scheme 1:**
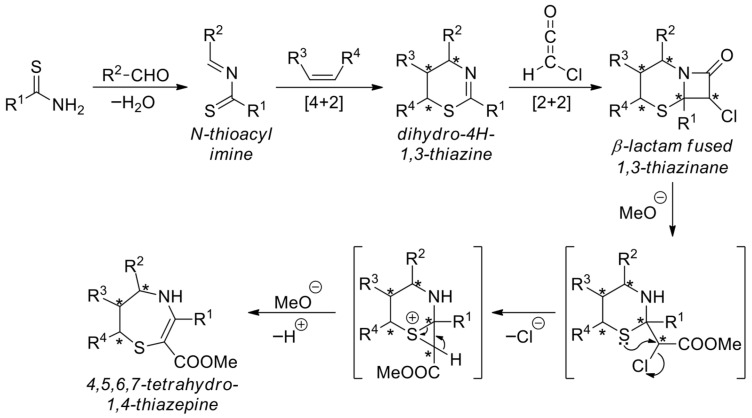
Transformation of alkenes into 5,6-dihydro-4*H*-1,3-thiazines, β-lactam-fused 1,3-thiazinanes, and 4,5,6,7-tetrahydro-1,4-thiazepines.

Fodor and coworkers reported that [2+2] cycloaddition of 5,6-dihydro-4*H*-1,3-thiazines and chloroketene yields chlorinated, β-lactam-fused 1,3-thiazinanes [29,30,31]. They also reported that treatment of the latter compounds (which show some structural similarities with cephems) with alkoxide ions provides 4,5,6,7-tetrahydro-1,4-thiazepines [30,31]. Recently, Peudru et al. improved this pathway by synthesizing the necessary dihydro-1,3-thiazines by the hetero-Diels–Alder reaction of alkenes and in situ generated *N*-thioacyl imines [32]. This modified pathway enables quick and efficient transformation of alkenes into various sulfur-containing azaheterocycles (Scheme 1).

Taking into account the increasing interest in the derivatives of 1,3-thiazines and 1,4-thiazepines, our aim was to use the synthetic method of Peudru et al. [32], utilizing various readily available cycloalkenes for access to novel *N*,*S*-heterocyclic compounds. We were also interested in the stereochemical outcomes of the reactions. Note that the hetero-Diels–Alder reaction creates three new stereocenters, and two additional centers are formed in the Staudinger ketene–imine cycloaddition.

## 2. Results and Discussion

The synthesis of 5,6-dihydro-4*H*-1,3-thiazines is a three-component, one-pot reaction (see Scheme 1). Thioamides and aldehydes, applied in constructing the *N*-thioacyl imine heterodienes, were chosen based on availability: thioacetamide (**8a**, R^1^ = Me), thiobenzamide (**8b**, R^1^ = Ph), and benzaldehyde (**9a**, R^2^ = Ph). [Some reactions were also performed with 4-chlorobenzaldehyde (**9b**, R^2^ = 4-chlorophenyl)]. The cycloalkane reaction partners were more diverse and included norbornadiene, indene, 1,5-cyclooctadiene, 1,3-cyclooctadiene, and 1,3-cyclohexadiene.

**Scheme 2:**
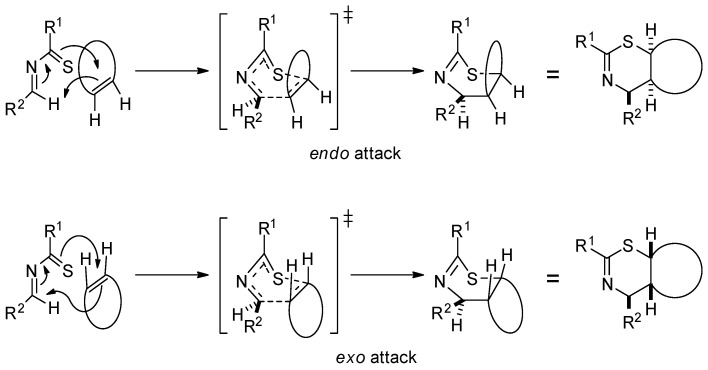
Relative configurations of the stereocenters generated by the hetero-Diels–Alder reaction. In the depicted conformation of *N*-thioacyl imines, steric repulsion between the large R groups and the big sulfur atom is minimized, and the heterodiene part is in the *s-cis* conformation required for the [4+2] cycloaddition.

It was already mentioned briefly that [4+2] cycloaddition of alkenes and *N*-thioacyl imines generate three new chiral centers. Luckily, as long as the reaction is concerted in nature, there are only two possible pathways (Scheme 2). The *exo* attack is favored by steric repulsion between the reactants (it results in a less crowded transition state), while the *endo* approach is favored by attractive van der Waals or π–π interactions between the reagents and the reactants.

**Scheme 3:**
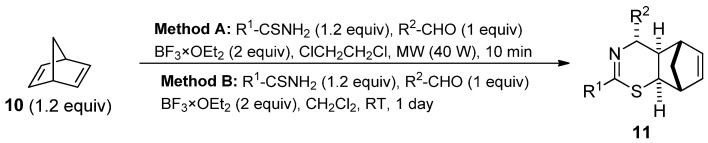
Synthesis of 5,6-dihydro-4*H*-1,3-thiazines from norbornadiene.

First, reactions of norbornadiene **10** were performed (Scheme 3). Utilizing the conditions described by Peudru et al. [1.2 mmol thioamide, 1 mmol aldehyde, 1.2 mmol alkene, 2 mmol BF_3_×OEt_2_, 1,2-dichloroethane, microwave heating for 10 min under 40 W (T ≤ 150 °C)], thiazine derivatives **11aa**, **11ab**, **11ba**, and **11bb** were obtained as single diastereoisomers in reasonable yields. Afterwards, we experimented with slight changes in the conditions. The results mostly agreed with those reported by Peudru et al. Dichloromethane provided results comparable to those of 1,2-dichloroethane, but its lower boiling point (42 °C) made its heating less convenient. Utilization of 2 mmol trifluoroacetic acid instead of BF_3_×OEt_2_ provided inferior yields. Without microwave heating (at RT), the reaction time lengthened to one day and the yield was somewhat lowered, but this setup was more convenient. Therefore, numerous hetero-Diels–Alder reactions were performed under these low-temperature conditions, with CH_2_Cl_2_ as the solvent. The most important results of the reactions with norbornadiene are summarized in Table 1.

**Figure 3:**
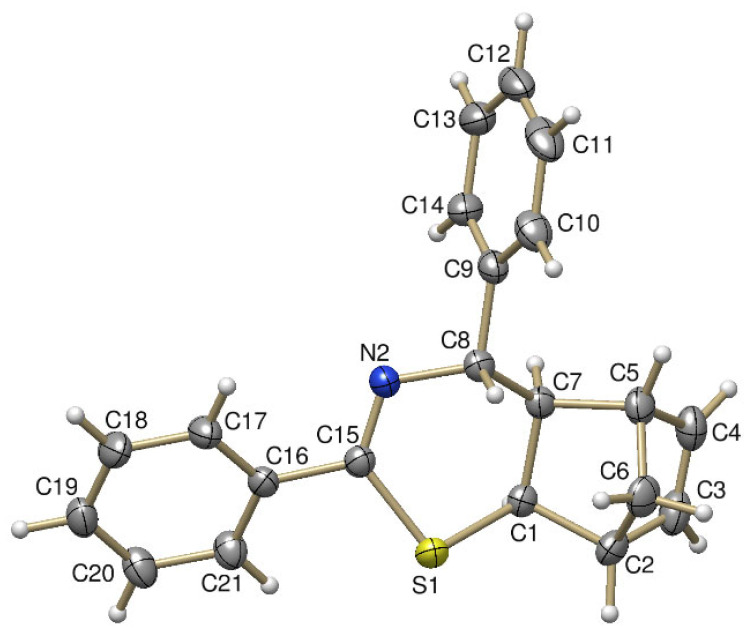
X-ray structure of product **11ba**.

**Scheme 4:**
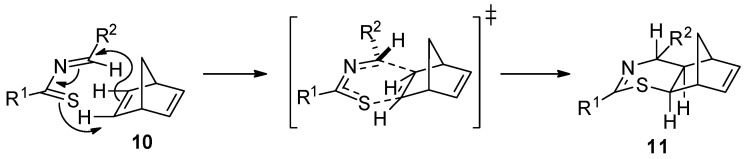
Relative arrangement of norbornadiene and the heterodiene during cycloaddition.

Stereochemistry of the products depicted in Scheme 3 was determined with the help of 2D NOESY measurements. In the case of **11ba**, single-crystal X-ray diffraction also corroborated the structure (Figure 3). Apparently, the reaction of *N*-thioacyl imines with norbornadiene is controlled by steric repulsion. Namely, the heterodiene approaches the olefin bond from the less hindered side (where the bridging methylene is found), and it performs an *exo* attack (Scheme 4).

**Scheme 5:**
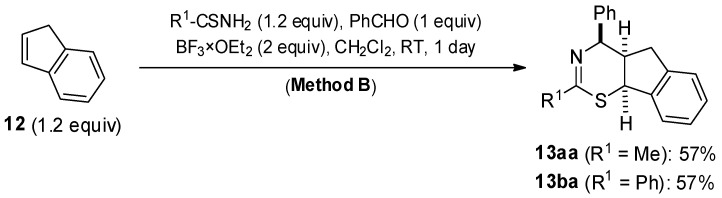
Synthesis of 5,6-dihydro-4*H*-1,3-thiazines from indene.

**Figure 4:**
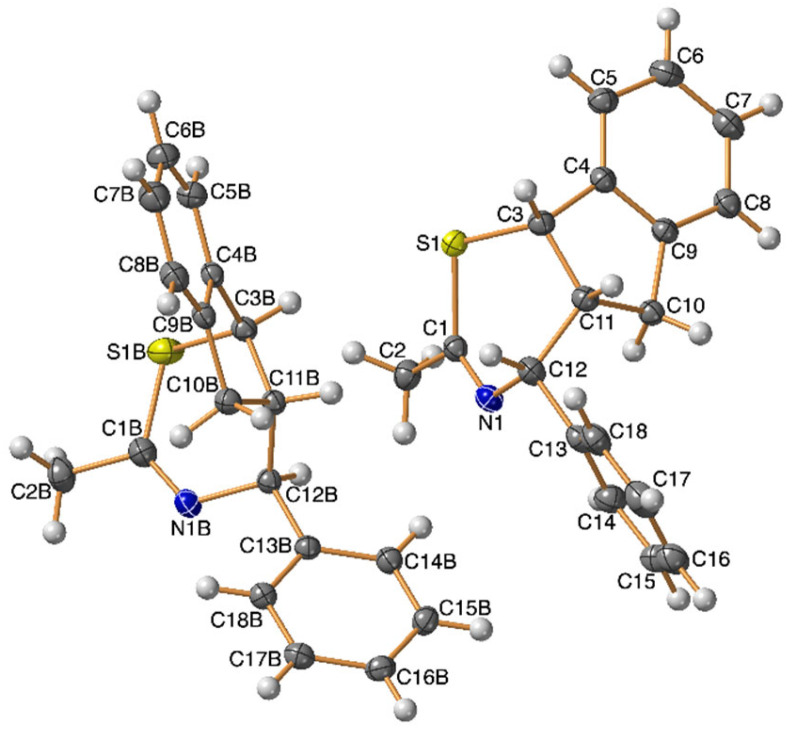
X-ray structure of product **13aa**.

We continued our experiments with indene. In this case, despite the non-symmetric olefin bond, the heterocyclic products were formed as single regio- and diastereoisomers (Scheme 5). The structure of the products was determined with the help of 2D NMR methods (including NOESY), and the structure of **13aa** was corroborated by single-crystal X-ray diffraction (Figure 4).

**Scheme 6:**
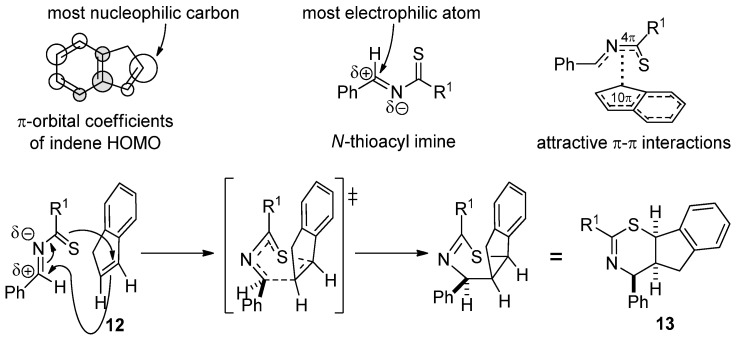
Explanation of the regio- and stereoselectivity of the hetero-Diels–Alder reaction of indene with *N*-thioacyl imines.

The same regioselectivity was observed in other indene–heterodiene Diels–Alder reactions [33,34]. The regioselectivity can be explained by HOMO–LUMO interactions: the HOMO of indene has the highest coefficients at C-2 (this is the most nucleophilic atom) [35], while the most electrophilic atom of the *N*-thioacyl imine is the imine carbon (the electronegative nitrogen withdraws electrons from it via both –I and –M effects). Based on the stereochemistry, the heterodiene performs an *endo* attack. Presumably, energetically favorable π–π interactions between the π-electron systems of indene and *N*-thioacyl imine are responsible for orienting the heterodiene. The above considerations are summarized in Scheme 6.

**Scheme 7:**
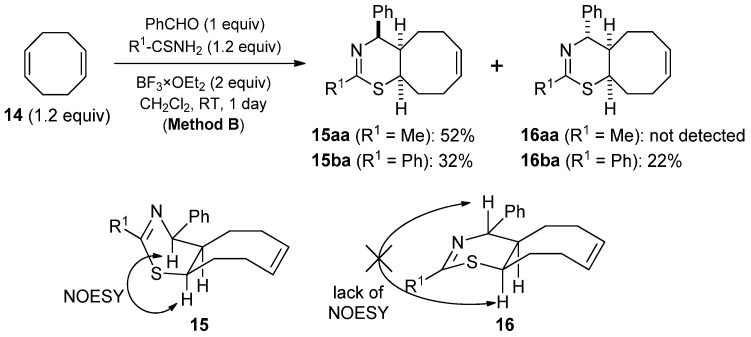
[4+2] Cycloaddition of 1,5-cyclooctadiene with *N*-thioacyl imines and key NOESY interactions in products **15** and **16**.

Afterwards, reactions of 1,5-cyclooctadiene were investigated. In this case, stereoselectivity of the reaction depended on the substituents of the *N*-thioacyl imine. If R^1^ was methyl, only *endo* product **15aa** was formed. If R^1^ was phenyl, however, the hetero-Diels–Alder reaction produced about a 3:2 mixture of *endo* product **15ba** and *exo* product **16ba** (Scheme 7). Stereochemistry of the products was determined by 2D NOESY (Scheme 7). Apparently, the less bulky *N*-thioacetyl imine (R^1^ = Me) can perform an *endo* attack on 1,5-cyclooctadiene without any difficulty. However, the bulkier *N*-thiobenzoyl imine (R^1^ = Ph) experiences more steric hindrance, which enables the *exo* attack to compete successfully with the *endo* attack (the *exo* attack proceeds through a less crowded transition state).

**Scheme 8:**
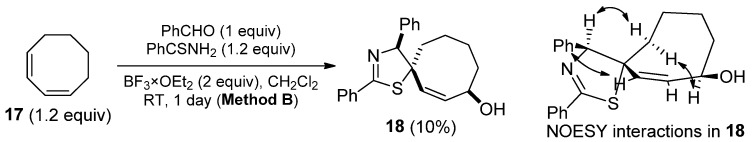
Attempted hetero-Diels–Alder reaction of 1,3-cyclooctadiene and key NOESY interactions in product **18**.

**Scheme 9:**
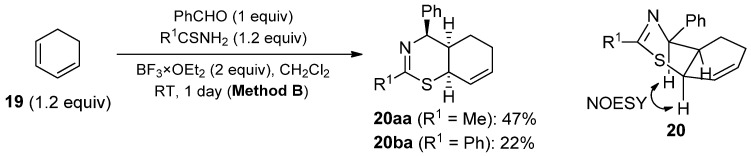
Synthesis of 5,6-dihydro-4*H*-1,3-thiazines **20** from 1,3-cyclohexadiene and key NOESY interactions in these products.

We were interested in extending the hetero-Diels–Alder reaction to conjugated dienes. First, 1,3-cyclooctadiene was reacted with benzaldehyde and thiobenzamide. At the end of the reaction (and after workup), TLC indicated the presence of a single product, but attempts to purify the reaction mixture via column chromatography on silica gel resulted in serious decomposition. In the end, from the various compounds which appeared in the fractions of the column, we were only able to isolate compound **18** in pure form. The structure of this molecule (determined by 2D NMR methods) was very surprising. In comparison to the expected fused-ring hetero-Diels–Alder product (chemical formula: C_22_H_23_NS), it contained an extra oxygen atom (chemical formula of **18**: C_22_H_23_NOS), and it had a spirocyclic structure (Scheme 8). Compound **18** has three stereocenters (including a quaternary carbon center), but luckily, NOESY enabled determination of the configuration of all centers (Scheme 8). At first, we assumed that the conjugated nature of the olefin reactant may be responsible for this unexpected outcome. However, the transformation of 1,3-cyclohexadiene (another conjugated diene) proceeded normally, providing hetero-Diels–Alder products **20** under these conditions (see Scheme 9). In the end, apart from suspecting that the decomposition of the initial product is caused by the acidity of silica gel and that the source of the unexpected oxygen atom is probably the O_2_ content of air, we could not solve the formation mechanism of compound **18**. This prompted us to discontinue investigating the reactions of 1,3-cyclooctadiene with other aldehydes and thioamides.

As mentioned above, there was a possibility that the puzzling formation of product **18** is connected to the conjugated diene nature of the alkene reactant. To test this hypothesis, we attempted the transformation of 1,3-cyclohexadiene. Interestingly, these reactions proceeded normally. Hetero-Diels–Alder products **20aa** and **20ba** formed with complete regio- and stereoselectivity (Scheme 9). Stereochemistry of the products was determined by 2D NOESY (Scheme 9).

**Scheme 10:**
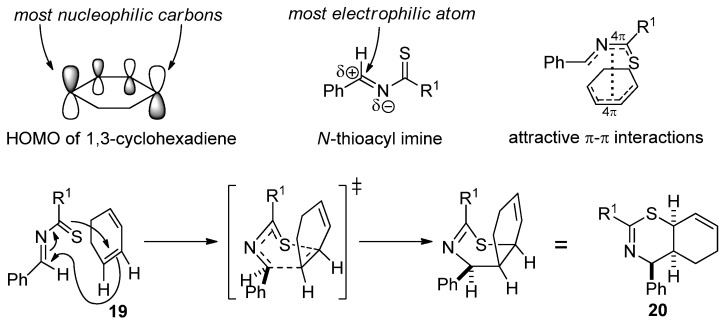
Explanation of the regio- and stereoselectivity of the hetero-Diels–Alder reaction of 1,3-cyclohexadiene with *N*-thioacyl imines.

The same regio- and stereoselectivity was observed in the hetero-Diels–Alder reactions of 1,3-cyclohexadiene and α-keto-β,γ-unsaturated phosphonates such as (*E*)-diethyl cinnamoylphosphonate [36]. The regioselectivity can be explained by HOMO–LUMO interactions: the HOMO of conjugated dienes has the highest coefficients at the ends of the conjugated system (these are the most nucleophilic atoms) [37], while the most electrophilic atom of the *N*-thioacyl imine is the imine carbon (the electronegative nitrogen withdraws electrons from it via both –I and –M effects). The *endo* stereoselectivity can be explained by the favorable π–π interactions between the conjugated electron systems of the heterodiene and the cyclodiene. These factors are summarized in Scheme 10.

**Scheme 11:**
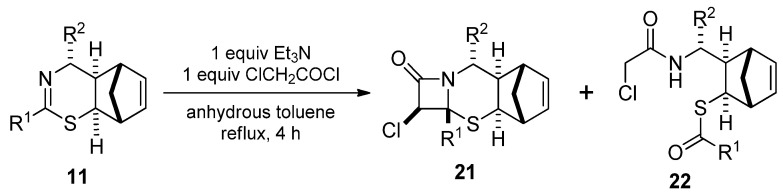
Staudinger ketene–imine cycloaddition of 5,6-dihydro-4*H*-1,3-thiazines **11**.

We continued with the transformation of norbornadiene-derived 5,6-dihydro-4*H*-1,3-thiazines to chlorinated β-lactam-fused 1,3-thiazines by treating them with chloroacetylchloride and triethylamine (these reagents generate chloroketene in situ) at 110 °C in anhydrous toluene. Mixed results were detected (Scheme 11). Compounds **11ba** and **11bb** provided the desired β-lactams **21ba** and **21bb** as single diastereoisomers (note that the Staudinger ketene–imine cycloaddition generates two new chiral centers). In contrast, compound **11ab** provided a mixture of β-lactam **21ab** (as a single diastereoisomer) and ring-opened thioester **22ab**, while compound **22aa** provided solely ring-opened thioester **22aa** (Table 2).

**Figure 5:**
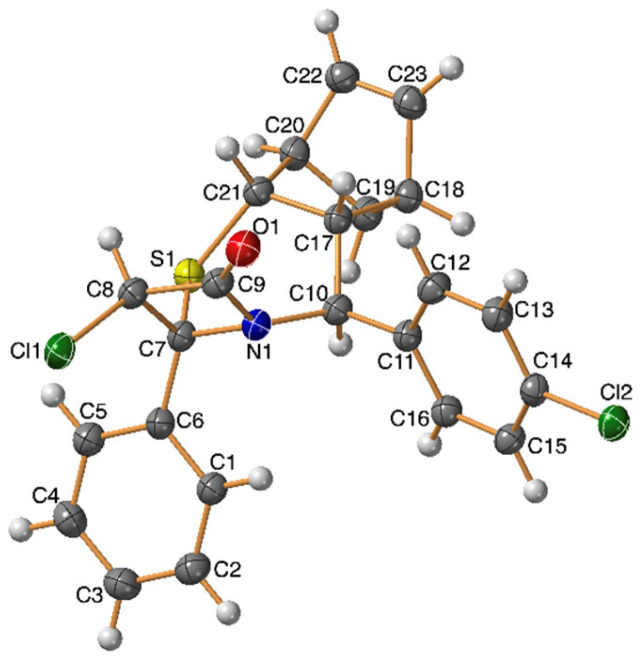
X-ray structure of product **21bb**.

The structure and stereochemistry of β-lactam **21bb** were unequivocally established by single-crystal X-ray diffraction (Figure 5), whereas the stereochemistry of the other synthesized β-lactams was assigned by analogy (in the case of **21ab**, 2D NOESY corroborates this assignment). The observed stereoselectivity of β-lactam formation (the Cl and the R^1^ groups are on the same side, and the ring-junction hydrogens are on the other side) is in agreement with literature data [38].

**Scheme 12:**
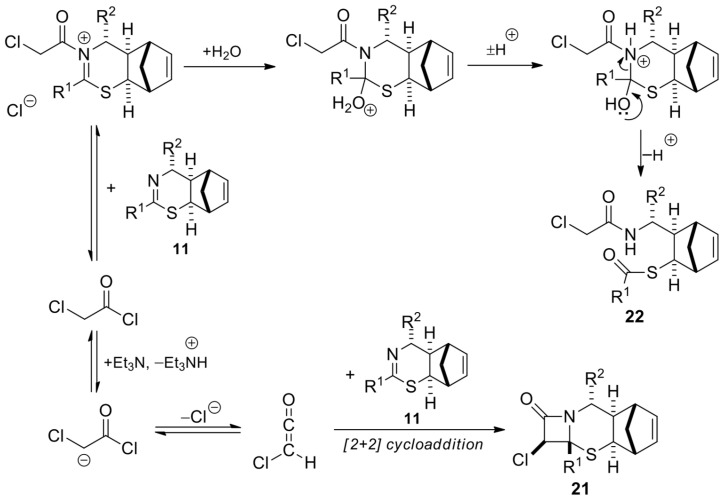
Competing processes during Staudinger ketene–imine cycloaddition and the effect of water on the reaction.

To explain the formation of the ring-opened thioesters, we have to consider that, under the reaction conditions, two processes compete for chloroacetylchloride (Scheme 12). *N*-Acylation of the dihydrothiazine derivative is probably rapid but reversible (similar to *N*-acylation of pyridine). Deprotonation of ClCH_2_COCl by Et_3_N is probably slow like all C–H deprotonations, but the resulting chloroketene can irreversibly perform [2+2] cycloaddition with the dihydrothiazine molecule. As long as water is absent, the irreversibility of the cycloaddition ensures that the β-lactam will be the sole product (although it may take some time). In the presence of water, however, the *N*-acylated dihydrothiazine derivative undergoes rapid and irreversible hydrolysis into the ring-opened thioester. (Note that imines are more nucleophilic than water, that is, chloroacetylchloride can *N*-acylate dihydrothiazine even in the presence of some water.) Although serious precautions were taken to keep the reaction mixtures anhydrous (utilization of CaCl_2_-filled drying tubes, use of anhydrous toluene as solvent, etc.), the observed product distribution strongly suggests that complete exclusion of water is difficult, and even minute amounts of humidity can interfere with β-lactam synthesis to a varying extent.

**Scheme 13:**
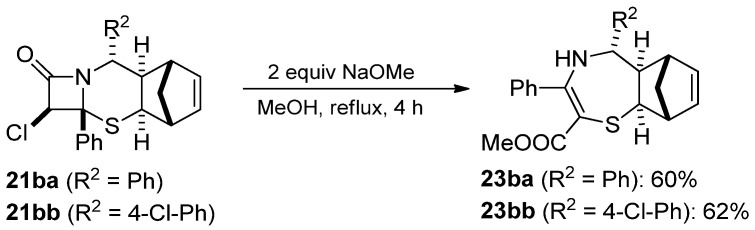
Ring expansion of β-lactam-fused 1,3-thiazines **21ba** and **21bb**.

Finally, chlorinated β-lactam-fused 1,3-thiazines **21ba** and **21bb** were treated with NaOMe in MeOH. The ring expansion process was successfully triggered (see Scheme 1), providing the expected 4,5,6,7-tetrahydro-1,4-thiazepines **23ba** and **23bb** in good yields (Scheme 13).

## 3. Materials and Methods

### 3.1. General Methods

Chemicals were purchased from Molar Chemicals Ltd., Halásztelek, Hungary; Merck Ltd., Budapest, Hungary, and VWR International Ltd., Debrecen, Hungary. Solvents were used as received from the suppliers. Melting points were determined with a Kofler apparatus (Nagema, Dresden, Germany). TLC plates (TLC Silica gel 60 F_254_) and silica gel for column chromatography (technical grade, pore size 60 Å, 70–230 mesh) were purchased from Merck Ltd., Budapest, Hungary.

NMR spectra were acquired at room temperature on a Bruker Avance 400 spectrometer (Flextra-Lab Ltd., Budapest, Hungary) with 9.39 T magnetic field strength (^1^H frequency: 400.13 MHz, ^13^C frequency: 100.76 MHz, respectively), on a Bruker Avance Neo 500 spectrometer (Flextra-Lab Ltd., Budapest, Hungary) with 11.75 T magnetic field strength (^1^H frequency 500.20 MHz, ^13^C frequency 125.78 MHz), or on a Bruker Avance III 600 spectrometer (Flextra-Lab Ltd., Budapest, Hungary) with 14.10 T magnetic field (^1^H frequency 600.20 MHz, ^13^C frequency 150.92 MHz) in CDCl_3_ or D_6_-DMSO, using the deuterium signal of the solvent to lock the field. The ^1^H and ^13^C chemical shifts are given relative to TMS.

HRMS were acquired on a Thermo Scientific Q-Exactive Plus Orbitrap mass spectrometer (Thermo Fisher Scientific Inc., Budapest, Hungary) equipped with an electrospray ionization ion source.

Single-crystal data of compounds **11ba**, **13aa**, and **21bb** were collected on a SuperNova, Dualflex, HyPix-Arc 100 diffractometer (Rigaku Oxford Diffraction, Wrocław, Poland) at 120(2) K. The crystal of **11ba** was solved in the orthorhombic space group Pbca. The unit-cell parameters were as follows: a = 11.7009(2) Å, b = 15.6961(3) Å, c = 17.6878(4) Å, and V = 3248.53(10) Å^3^. The crystal of **13aa** was solved in the monoclinic space group P2_1_/n. The unit-cell parameters were as follows: a = 8.25120(7) Å, b = 16.50920(13) Å, c = 21.02664(18) Å, β = 94.1989(7)°, and V = 2856.58(4) Å^3^. The crystal of **21bb** was solved in the monoclinic space group P2_1_/c. The unit-cell parameters were as follows: a = 13.43125(18) Å, b = 6.82967(9) Å, c = 21.6001(3) Å, β = 98.5980(12)°, and V = 1959.13(4) Å^3^. The crystallographic parameters are given in Section 3 (“X-ray structure determinations”) of the Supporting Material [39,40,41,42,43].

### 3.2. General Methods for the Synthesis of Thiazines

**Method A**: In a 10 mL microwave reaction vial, 1.2 mmol thioamide, 1 mmol aldehyde, and 1.2 mmol cyclic olefin were dissolved in 10 mL 1,2-dichloroethane. To this mixture, 2 mmol (0.25 mL) boron trifluoride etherate (BF_3_×OEt_2_) was added dropwise at RT. After that, the vial was sealed and irradiated at 40 W power in a microwave reactor for 10 min (T ≤ 150 °C) with stirring. After cooling to room temperature, the reaction mixture was washed with 10 mL saturated aqueous NaHCO_3_ solution, then the aqueous phase was extracted with 3×15 mL dichloromethane. The combined organic phase was dried on Na_2_SO_4_. After the drying agent was filtered out, the resulting filtrate was evaporated and purified via column chromatography on silica gel. Sometimes, after column chromatography, recrystallization was also necessary to obtain a pure product.

**Method B**: Similar to Method A, but CH_2_Cl_2_ was used as a solvent, and the reaction mixture was stirred at RT for 24 h (microwave irradiation was omitted).

### 3.3. General Method for the Staudinger Ketene–Imine Cycloaddition of Thiazines

In a three-necked round-bottom flask, 3.0 mmol thiazine was dissolved in 30 mL anhydrous toluene. The stirred reaction mixture was heated to reflux. From two dropping funnels, a chloroacetylchloride solution (1.0 mmol ClCH_2_COCl dissolved in 10 mL anhydrous toluene) and a triethylamine solution (1.0 mmol Et_3_N dissolved in 10 mL anhydrous toluene) were added slowly (under about 1 h) to the reaction mixture under reflux. The “reagent addition step” described in the previous sentence was carried out three times. Then reflux was continued until the overall reaction time reached 4 h. After that, the reaction mixture was evaporated and purified via column chromatography on silica gel (eluent: *n*-hexane/EtOAc 10:1). The raw product was purified further via recrystallization.

### 3.4. General Method for the Ring Expansion of β-Lactam Condensed Thiazinanes

An amount of 0.16 mmol β-lactam condensed 1,3-thiazinane and 2 equiv NaOMe were dissolved in 2 mL methanol. Under an argon atmosphere, the reaction mixture was treated under reflux for 4 h. Afterwards, the reaction mixture was concentrated under reduced pressure. The residue was dissolved in 4 mL water and extracted with 4 × 10 mL CH_2_Cl_2_. The organic phase was dried on Na_2_SO_4_. After the drying agent was filtered out, the resulting filtrate was evaporated and purified via column chromatography on silica gel and subsequent recrystallization.

### 3.5. Characterization of Compounds

(4S*,4aR*,5S*,8R*,8aR*)-2-methyl-4-phenyl-4a,5,8,8a-tetrahydro-4*H*-5,8-methanobenzo[*e*][1,3]thiazine (**11aa**)

Formed diastereoselectively from thioacetamide (**8a**), benzaldehyde (**9a**), and norbornadiene (**10**) via *General methods for the synthesis of thiazines*. Purification: column chromatography (eluent: *n*-hexane/EtOAc 4:1) followed by crystallization. White powder yield as follows: 55% (*Method A*) or 45% (*Method B*). R_f_ = 0.6 (*n*-hexane/EtOAc 4:1). Mp. 88–89 °C.

^1^H NMR (500 MHz, CDCl_3_): *δ* (ppm) = 1.52–1.58 (m, 1H, H-9), 1.83–1.91 (m, 1H, H-4a), 2.32 (s, 3H, CH_3_), 2.37–2.43 (m, 1H, H-9), 2.58–2.62 (m, 1H, H-5), 2.86–2.90 (m, 1H, H-8), 3.05–3.09 (m, 1H, H-8a), 3.77–3.83 (m, 1H, H-4), 6.02–6.06 (m, 1H, H-7), 6.06–6.11 (m, 1H, H-6), 7.27–7.33 (m, 1H, Ar), and 7.35–7.42 (m, 4H, Ar).

^13^C NMR (125 MHz, CDCl_3_): *δ* (ppm) = 28.9, 43.3, 46.0, 46.2, 48.8, 50.5, 70.4, 127.1, 128.3, 128.5, 135.5, 139.4, 144.2, and 165.2.

HRMS calcd. for C_16_H_18_NS^+^ ([M+H]^+^): 256.1154. Found: 256.1150.

(4S*,4aR*,5S*,8R*,8aR*)-2-methyl-4-(4-chlorophenyl)-4a,5,8,8a-tetrahydro-4*H*-5,8-methanobenzo[*e*][1,3]thiazine (**11ab**)

Formed diastereoselectively from thioacetamide (**8a**), 4-chlorobenzaldehyde (**9b**), and norbornadiene (**10**) via *General methods for the synthesis of thiazines*. Purification: column chromatography (eluent: *n*-hexane/EtOAc 4:1). White solid yield as follows: 56% (*Method A*). R_f_ = 0.5 (*n*-hexane/EtOAc 4:1). Mp. 126–128 °C.

^1^H NMR (400 MHz, CDCl_3_): *δ* (ppm) = 1.52–1.59 (m, 1H, H-9), 1.77–1.86 (m, 1H, H-4a), 2.32 (d, *J* = 1.67 Hz, 3H, CH_3_), 2.34–2.40 (m, 1H, H-9), 2.53–2.59 (m, 1H, H-5), 2.86–2.92 (m, 1H, H-8), 3.03–3.10 (m, 1H, H-8a), 3.74–3.82 (m, 1H, H-4), 6.02–6.06 (m, 1H, H-7), 6.06–6.12 (m, 1H, H-6), 7.29–7.34 (m, 2H, Ar), and 7.34–7.40 (m, 2H, Ar).

^13^C NMR (125 MHz, CDCl_3_): *δ* (ppm) = 28.8, 43.2, 45.9, 46.1, 48.8, 50.6, 69.7, 128.7, 129.7, 132.9, 135.6, 139.2, 142.7, and 165.7.

HRMS calcd. for C_16_H_17_ClNS^+^ ([M+H]^+^): 290.0765. Found: 290.0762.

(4S*,4aR*,5S*,8R*,8aR*)-2,4-diphenyl-4a,5,8,8a-tetrahydro-4*H*-5,8-methanobenzo[*e*][1,3]thiazine (**11ba**)

Formed diastereoselectively from thiobenzamide (**8b**), benzaldehyde (**9a**), and norbornadiene (**10**) via *General methods for the synthesis of thiazines*. Purification: column chromatography (eluent: *n*-hexane/EtOAc 4:1) followed by crystallization. White powder yield as follows: 58% (*Method A*) or 50% (*Method B*). R_f_ = 0.6 (*n*-hexane/EtOAc 4:1). Mp. 130–134 °C.

^1^H NMR (500 MHz, CDCl_3_): *δ* (ppm) = 1.63 (d, *J* = 9.05 Hz, 1H, H-9), 1.97–2.03 (m, 1H, H-4a), 2.54 (d, *J* = 9.04 Hz, 1H, H-9), 2.68–2.72 (m, 1H, H-5), 2.99–3.04 (m, 1H, H-8), 3.16 (d, *J* = 7.78 Hz, 1H, H-8a), 4.11 (d, *J* = 10.83 Hz, 1H, H-4), 6.05–6.10 (m, 1H, H-7), 6.10–6.14 (m, 1H, H-6), 7.30–7.39 (m, 3H, Ar), 7.39–7.45 (m, 3H, Ar), 7.47–7.53 (m, 2H, Ar), and 7.98–8.04 (m, 2H, Ar).

^13^C NMR (125 MHz, CDCl_3_): *δ* (ppm) = 43.4, 46.1, 47.2, 48.9, 52.6, 71.4, 127.1, 127.6, 128.3, 128.4, 128.6, 130.8, 135.6, 138.3, 139.7, 144.4, and 165.6.

HRMS calcd. for C_21_H_20_NS^+^ ([M+H]^+^): 318.1311. Found: 318.1305.

(4S*,4aR*,5S*,8R*,8aR*)-4-(4-chlorophenyl)-2-phenyl-4a,5,8,8a-tetrahydro-4*H*-5,8-methanobenzo[*e*][1,3]thiazine (**11bb**)

Formed diastereoselectively from thiobenzamide (**8b**), 4-chlorobenzaldehyde (**9b**), and norbornadiene (**10**) via *General methods for the synthesis of thiazines*. Purification: column chromatography (eluent: *n*-hexane/EtOAc 20:1) followed by a second column chromatography (eluent: *n*-hexane/PhMe 3:1). Colorless oil yield as follows: 36% (*Method A*) or 28% (*Method B*). R_f_ = 0.6 (*n*-hexane/EtOAc 20:1).

^1^H NMR (500 MHz, CDCl_3_): *δ* (ppm) = 1.63 (d, *J* = 9.09 Hz, 1H, H-9), 1.91–1.98 (m, 1H, H-4a), 2.51 (d, *J* = 9.08 Hz, 1H, H-9), 2.64–2.69 (m, 1H, H-5), 2.99–3.04 (m, 1H, H-8), 3.13–3.18 (m, 1H, H-8a), 4.09 (d, *J* = 10.89 Hz, 1H, H-4), 6.06–6.10 (m, 1H, H-7), 6.10–6.15 (m, 1H, H-6), 7.34–7.41 (m, 4H, Ar), 7.41–7.46 (m, 3H, Ar), and 7.97–8.02 (m, 2H, Ar).

^13^C NMR (125 MHz, CDCl_3_): *δ* (ppm) = 43.4, 46.1, 47.1, 48.8, 52.7, 70.7, 127.5, 128.3, 128.5, 129.9, 130.9, 132.9, 135.7, 138.1, 139.5, 142.9, and 166.0.

HRMS calcd. for C_21_H_19_ClNS^+^ ([M+H]^+^): 352.8997. Found: 352.0917.

(4R*,4aR*,9bS*)-4-phenyl-2-methyl-4,4a,5,9b-tetrahydroindeno[2,1-*e*][1,3]thiazine (**13aa**)

Formed regio- and diastereoselectively from thioacetamide (**8a**), benzaldehyde (**9a**), and indene (**12**) via *General methods for the synthesis of thiazines*. Purification: column chromatography (eluent: *n*-hexane/EtOAc 4:1) followed by crystallization. White solid yield as follows: 57% (*Method B*). R_f_ = 0.4 (*n*-hexane/EtOAc 4:1). Mp. 124 °C.

^1^H NMR (500 MHz, CDCl_3_): *δ* (ppm) = 2.25 (d, *J* = 1.91 Hz, 3H, CH_3_), 2.39 (dd, *J* = 15.95 Hz, *J* = 7.53 Hz, 1H, H-5), 2.89 (dd, *J* = 15.89 Hz, *J* = 10.56 Hz, 1H, H-5), 3.18–3.26 (m 1H, H-4a), 4.60–4.65 (m, 1H, H-4), 4.98 (d, *J* = 8.13 Hz, 1H, H-9b), and 7.04–7.51 ppm (9H, m, Ar).

^13^C NMR (125 MHz, CDCl_3_) *δ* (ppm) = 28.6, 30.4, 44.1, 49.4, 62.7, 124.2, 125.3, 126.7, 127.0, 127.2, 128.1, 128.5, 142.0, 143.7, 144.5, and 163.0.

HRMS calcd. for C_18_H_18_NS^+^ ([M+H]^+^): 280.1154. Found: 280.1150.

(4R*,4aR*,9bS*)-2,4-diphenyl-4,4a,5,9b-tetrahydroindeno[2,1-*e*][1,3]thiazine (**13ba**)

Formed regio- and diastereoselectively from thiobenzamide (**8b**), benzaldehyde (**9a**), and indene (**12**) via *General methods for the synthesis of thiazines*. Purification: column chromatography (eluent: *n*-hexane/EtOAc 4:1) followed by crystallization. White solid yield as follows: 57% (*Method B*). R_f_ = 0.7 (*n*-hexane/EtOAc 4:1). Mp. 141–142 °C.

^1^H NMR (500 MHz, D_6_-DMSO): *δ* (ppm) = 2.42 (dd, *J* = 16.25 Hz, *J* = 7.87 Hz, 1H, H-5), 2.91 (dd, *J* = 15.95 Hz, *J* = 10.06 Hz, 1H, H-5), 3.55–3.63 (m, 1H, H-4a), 4.99–5.11 (m, 1H, H-4), 5.40 (d, *J* = 8.23 Hz, 1H, H-9b), 7.09–7.16 (m,1H, Ar), 7.16–7.24 (m, 2H, Ar), 7.33–7.40 (m, 1H, Ar), 7.41–7.51 (m, 5H, Ar), 7.52–7.59 (m, 1H, Ar), 7.64–7.70 (m, 2H, Ar), and 7.80–7.85 (m, 2H, Ar).

^13^C NMR (125 MHz, CDCl_3_): *δ* (ppm) = 30.8, 45.5, 50.4, 64.2, 124.6, 125.1, 126.7, 127.0, 127.4, 128.1, 128.2, 128.4, 130.6, 139.0, 142.0, 143.7, 144.5, and 164.0 ppm.

HRMS calcd. for C_23_H_20_NS^+^ ([M+H]^+^): 342.1311. Found: 342.1306.

(4R*,4aR*,10aR*,*Z*)-2-methyl-4-phenyl-4a,5,6,9,10,10a-hexahydro-4*H*-cycloocta[*e*][1,3]thiazine (**15aa**)

Formed from thioacetamide (**8a**), benzaldehyde (**9a**), and 1,5-cyclooctadiene (**14**) via *General methods for the synthesis of thiazines*. Purification: column chromatography (eluent: *n*-hexane/EtOAc 14:1) followed by crystallization. White solid yield as follows: 52% (*Method B*). R_f_ = 0.3 (*n*-hexane/EtOAc 14:1). Mp. 137–138 °C.

^1^H NMR (500 MHz, CDCl_3_): *δ* (ppm) = 0.92–1.03 (m, 1H, H-5), 1.51–1.62 (m, 1H, H-5), 1.73–1.88 (m, 2H, H-6 and H-10), 2.18–2.34 (m, 5H, CH_3_ and H-9 and H-10), 2.45–2.58 (m, 2H, H-4a and H-9), 2.58–2.69 (m, 1H, H-6), 3.97–4.04 (m, 1H, H-10a), 4.63–4.68 (m, 1H, H-4), 5.28–5.37 (m, 1H, H-7), 5.56–5.64 (m, 1H, H-8), 7.19–7.25 (m, 1H, Ar), and 7.30–7.40 (m, 4H, Ar).

^13^C NMR (125 MHz, CDCl_3_): *δ* (ppm) = 20.3, 23.8, 27.7, 28.8, 30.8, 34.1, 48.2, 66.6, 125.9, 126.1, 127.3, 128.1, 130.9, 143.6, and 158.9.

HRMS calcd. for C_17_H_22_NS^+^ ([M+H]^+^): 272.1467. Found: 272.1463.

(4R*,4aR*,10aR*,*Z*)-2,4-diphenyl-4a,5,6,9,10,10a-hexahydro-4*H*-cycloocta[*e*][1,3]thiazine (**15ba**)

Formed from thiobenzamide (**8b**), benzaldehyde (**9a**), and 1,5-cyclooctadiene (**14**) via *General methods for the synthesis of thiazines*. Purification: column chromatography (eluent: *n*-hexane/EtOAc 4:1), then a second round of column chromatography (eluent: *n*-hexane/EtOAc 14:1), and finally crystallization. White solid yield as follows: 32% (*Method B*). R_f_ = 0.5 (*n*-hexane/EtOAc 14:1). Mp. 69–72 °C.

^1^H NMR (500 MHz, CDCl_3_): *δ* (ppm) = 0.95–1.06 (m, 1H, H-5), 1.57–1.67 (m, 1H, H-5), 1.72–1.82 (m, 1H, H-6), 1.92–2.02 (m, 1H, H-10), 2.26–2.45 (m, 2H, H-9, H-10), 2.54–2.72 (m, 3H, H-4a, H-6, H-9), 4.09–4.16 (m, 1H, H-10a), 4.91 (d, *J* = 3.34 Hz, 1H, H-4), 5.30–5.41 (m, 1H, H-7), 5.59–5.68 (m, 1H, H-8), 7.23–7.26 (m, 1H, Ar), 7.34–7.47 (m, 5H, Ar), 7.47–7.52 (m, 2H, Ar), and 7.95–8.00 (m, 2H, Ar).

^13^C NMR (125 MHz, CDCl_3_): *δ* (ppm) = 20.7, 23.8, 28.8, 31.1, 34.2, 48.3, 66.8, 126.0, 126.4, 126.4, 127.3, 128.1, 128.3, 130.4, 130.9, 138.9, 143.6, and 159.2.

HRMS calcd. for C_22_H_24_NS^+^ ([M+H]^+^): 334.1624. Found: 334.1621.

(4S*,4aR*,10aR*,*Z*)-2,4-diphenyl-4a,5,6,9,10,10a-hexahydro-4*H*-cycloocta[*e*][1,3]thiazine (**16ba**)

Formed from thiobenzamide (**8b**), benzaldehyde (**9a**), and 1,5-cyclooctadiene (**14**) via *General methods for the synthesis of thiazines*. Purification: column chromatography (eluent: *n*-hexane/EtOAc 4:1), then a second round of column chromatography (eluent: *n*-hexane/EtOAc 14:1), and finally crystallization. White solid yield as follows: 22% (*Method B*). R_f_ = 0.3 (*n*-hexane/EtOAc 14:1). Mp. 116–118 °C.

^1^H NMR (500 MHz, CDCl_3_): *δ* (ppm) = 1.47–1.56 (m, 1H, H-5), 1.79–1.88 (m, 1H, H-10), 1.89–1.98 (m, 1H, H-9), 2.02–2.12 (m, 2H, H-5, H-6), 2.27–2.37 (m, 1H, H-10), 2.37–2.43 (m, 1H, H-4a), 2.48–2.57 (m, 1H, H-9), 2.67–2.79 (m, 1H, H-6), 3.38–3.47 (m, 1H, H-10a), 5.16 (d, *J* = 4.1 Hz, 1H, H-4), 5.53–5.67 (m, 2H, H-7, H-8), 7.16–7.21 (m, 2H, Ar), 7.22–7.27 (m, 1H, Ar), 7.30–7.36 (m, 2H, Ar), 7.37–7.46 (m, 3H, Ar), and 7.90–7.94 (m, 2H, Ar).

^13^C NMR (125 MHz, CDCl_3_): *δ* (ppm) = 25.4, 27.4, 28.9, 30.6, 36.3, 39.4, 66.9, 126.5, 126.9, 127.0, 127.1, 128.4, 128.5, 129.8, 130.6, 139.0, and 142.9.

HRMS calcd. for C_22_H_24_NS^+^ ([M+H]^+^): 334.1624. Found: 334.1620.

(4S*,5R*,8R*,*Z*)-2,4-diphenyl-1-thia-3-azaspiro[4.7]dodeca-2,6-dien-8-ol (**18**)

Formed from thiobenzamide (**8b**), benzaldehyde (**9a**), and 1,3-cyclooctadiene (**17**) via *General methods for the synthesis of thiazines*. Purification: column chromatography (eluent: *n*-hexane/EtOAc 7:2), then crystallization. White solid yield as follows: 10% (*Method B*). R_f_ = 0.3 (*n*-hexane/EtOAc 7:2). Mp. 160–165 °C.

^1^H NMR (600 MHz, D_6_-DMSO): *δ* (ppm) = 1.28–1.32 (m, 1H, H-9), 1.35–1.40 (m, 1H, H-10), 1.60–1.70 (m, 2H, H-10, H-11), 1.70–1.84 (m, 1H, H-9), 1.88–1.97 (m, 1H, H-11), 2.01–2.05 (m, 1H, H-12), 2.19–2.28 (m, 1H, H-12), 4.75–4.80 (m, 1H, H-8), 4.88 (d, *J* = 4.2 Hz, 1H, OH), 5.17–5.20 (m, 2H, H-6, H-7), 5.62 (s, 1H, H-4), 7.25–7.31 (m, 2H, Ar), 7.31–7.35 (m, 1H, Ar), 7.35–7.41 (m, 2H, Ar), 7.49–7.55 (m, 2H, Ar), 7.56–7.61 (m, 1H, Ar), and 7.82–7.89 (m, 2H, Ar).

^13^C NMR (150 MHz, D_6_-DMSO): *δ* (ppm) = 23.1, 23.5, 36.7, 42.2, 66.6, 68.4, 86.9, 127.2, 128.0, 128.3, 128.4, 128.9, 131.7, 132.8, 136.5, 136.9, and 166.9.

HRMS calcd. for C_22_H_24_NOS^+^ ([M+H]^+^): 350.1573. Found: 350.1568.

(4S*,4aS*,8aS*)-2-methyl-4-phenyl-4a,5,6,8a-tetrahydro-4*H*-benzo[*e*][1,3]thiazine (**20aa**)

Formed from thioacetamide (**8a**), benzaldehyde (**9a**), and 1,3-cyclohexadiene (**19**) via *General methods for the synthesis of thiazines*. Purification: column chromatography (eluent: *n*-hexane/EtOAc 4:1), then a second round of column chromatography (eluent: *n*-hexane/EtOAc 10:1), and finally crystallization. White solid yield as follows: 47% (*Method B*). R_f_ = 0.4 (*n*-hexane/EtOAc 10:1). Mp. 87–89 °C.

^1^H NMR (500 MHz, D_6_-DMSO): *δ* (ppm) = 1.23–1.42 (m, 2H, H-5), 1.85–1.97 (m, 1H, H-6), 2.03–2.17 (m, 2H, H-4a and H-6), 2.23–2.28 (m, 3H, CH_3_), 4.27–4.32 (m, 1H, H-8a), 4.47–4.51 (m, 1H, H-4), 5.64–5.70 (m, 1H, H-8), 5.77–5.83 (m, 1H, H-7), 7.23–7.29 (m, 1H, Ar), 7.34–7.40 (m, 2H, Ar), and 7.40–7.45 (m, 2H, Ar).

^13^C NMR (125 MHz, D_6_-DMSO): *δ* (ppm) = 15.5, 25.6, 27.9, 35.2, 41.5, 65.0, 125.8, 126.6, 127.5, 128.2, 130.0, 143.0, and 159.1.

HRMS calcd. for C_15_H_18_NS^+^ ([M+H]^+^): 244.1154. Found: 244.1153.

(4S*,4aS*,8aS*)-2,4-diphenyl-4a,5,6,8a-tetrahydro-4*H*-benzo[*e*][1,3]thiazine (**20ba**)

Formed from thiobenzamide (**8b**), benzaldehyde (**9a**), and 1,3-cyclohexadiene (**19**) via *General methods for the synthesis of thiazines*. Purification: column chromatography (eluent: *n*-hexane/EtOAc 20:1), and finally crystallization. White solid yield as follows: 22% (*Method B*). R_f_ = 0.3 (*n*-hexane/EtOAc 20:1). Mp. 98–99 °C.

^1^H NMR (500 MHz, D_6_-DMSO): *δ* (ppm) = 1.05–1.29 (m, 2H, H-5), 1.81–1.93 (m, 1H, H-6), 1.96–2.08 (m, 1H, H-6), 2.24–2.32 (m, 1H, H-4a), 4.55–4.63 (m, 1H, H-8a), 4.77 (d, *J* = 2.58 Hz, 1H, H-4), 5.76–5.83 (m, 2H, H-7 and H-8), 7.25–7.31 (m, 1H, Ar), 7.38–7.43 (m, 2H, Ar), 7.43–7.53 (m, 3H, Ar), 7.55–7.60 (m, 2H, Ar), and 7.84–7.89 (m, 2H, Ar).

^13^C NMR (125 MHz, D_6_-DMSO): *δ* (ppm) = 16.0, 25.5, 35.2, 41.4, 64.8, 126.5, 126.6, 126.9, 127.9, 128.5, 129.0, 130.1, 131.1, 139.1, 143.6, and 158.4.

HRMS calcd. for C_20_H_20_NS^+^ ([M+H]^+^): 306.1311. Found: 306.1306.

(2S*,2aR*,3aR*,4R*,7S*,7aR*,8S*)-2-chloro-8-(4-chlorophenyl)-2a-methyl-2,2a,3a,4,7a,8-hexahydro-4,7-methanoazeto[2,1-*b*]benzo[*e*][1,3]thiazin-1(7*H*)-one (**21ab**)

Formed from thiazine **11ab** via *General method for the Staudinger ketene–imine cycloaddition of thiazines*. White solid yield as follows: 12%. R_f_ = 0.45 (*n*-hexane/EtOAc 10:1). Mp. 158–162 °C.

^1^H NMR (500 MHz, CDCl_3_): *δ* (ppm) = 1.51–1.58 (m, 1H, CH_2_), 1.70 (s, 3H, CH_3_), 2.19–2.24 (m, 1H, CH_2_), 2.41–2.45 (m, 1H, H-7), 2.49 (dd, *J* = 12.22 Hz, *J* = 7.29 Hz, 1H, H-7a), 2.79–2.83 (m, 1H, H-4), 3.15 (d, *J* = 7.23 Hz, 1H, H-3a), 3.97 (d, *J* = 12.27 Hz, 1H, H-8), 5.02 (s, 1H, H-2), 5.99–6.04 (m, 1H, H-5), 6.14–6.20 (m, 1H, H-6), and 7.37 (d, *J* = 8.19 Hz, 2H, Ar), 7.57 (d, *J* = 8.21 Hz, 2H, Ar).

^13^C NMR (125 MHz, CDCl_3_): *δ* (ppm) = 22.2, 41.8, 43.5, 44.2, 44.5, 47.6, 61.0, 67.0, 68.1, 128.9, 130.6, 133.9, 134.4, 134.7, 139.3, and 165.2.

HRMS calcd. for C_18_H_18_Cl_2_NOS^+^ ([M+H]^+^): 366.0481. Found: 366.0478.

(2R*,2aS*,3aR*,4R*,7S*,7aR*,8S*)-2-chloro-2a,8-diphenyl-2,2a,3a,4,7a,8-hexahydro-4,7-methanoazeto[2,1-*b*]benzo[*e*][1,3]thiazin-1(7*H*)-one (**21ba**)

Formed diastereoselectively from thiazine **11ba** via *General method for the Staudinger ketene–imine cycloaddition of thiazines*. White solid yield as follows: 58%. R_f_ = 0.4 (*n*-hexane/EtOAc 10:1). Mp. 206–208 °C.

^1^H NMR (400 MHz, CDCl_3_): *δ* (ppm) = 1.46–1.49 (m, 1H, CH_2_), 2.14–2.24 (m, 1H, CH_2_), 2.41–2.46 (m, 1H, H-7a), 2.66–2.74 (m, 1H, H-7), 2.81–2.87 (m, 1H, H-4), 3.30 (d, *J* = 7.0 Hz, 1H, H-3a), 4.09–4.16 (m, 1H, H-8), 5.27 (s, 1H, H-2), 6.00–6.06 (m, 1H, H-5), 6.16–6.20 (m, 1H, H-6), 7.28–7.33 (m, 2H, Ar), 7.34–7.50 (m, 6H, Ar), and 7.70–7.77 (m, 2H, Ar).

^13^C NMR (125 MHz, CDCl_3_): *δ* (ppm) = 43.0, 43.5, 44.1, 44.5, 47.6, 63.3, 68.5, 73.6, 125.2, 128.6, 128.7, 128.8, 128.9, 129.5, 133.7, 136.0, 137.0, 139.7, and 165.5.

HRMS calcd. for C_23_H_21_ClNOS^+^ ([M+H]^+^): 394.1027. Found: 394.1025.

(2S*,2aR*,3aR*,4R*,7S*,7aR*,8S*)-2-chloro-2a-phenyl-8-(4-chlorophenyl)-2,2a,3a,4,7a,8-hexahydro-4,7-methanoazeto[2,1-*b*]benzo[*e*][1,3]thiazin-1(7*H*)-one (**21bb**)

Formed diastereoselectively from thiazine **11bb** via *General method for the Staudinger ketene–imine cycloaddition of thiazines*. White solid yield as follows: 56%. R_f_ = 0.6 (*n*-hexane/EtOAc 10:1). Mp. 208–210 °C.

^1^H NMR (400 MHz, CDCl_3_): *δ* (ppm) = 1.46–1.52 (m, 1H, CH_2_), 2.13–2.18 (m, 1H, CH_2_), 2.39–2.43 (m, 1H, H-7a), 2.60–2.68 (m, 1H, H-7), 2.83–2-87 (m, 1H, H-4), 3.27–3.31 (m, 1H, H-3a), 4.06–4.13 (m, 1H, H-8), 5.27 (s, 1H, H-2), 6.01–6.06 (m, 1H, H-5), 6.16–6.21 (m, 1H, H-6), 7.25–7.30 (m, 2H, Ar), 7.34–7.48 (m, 5H, Ar), and 7.66–7.72 (m, 2H, Ar).

^13^C NMR (125 MHz, CDCl_3_): *δ* (ppm) = 42.9, 43.5, 44.3, 44.4, 47.6, 62.6, 68.4, 73.6, 125.2, 128.8, 128.9, 129.1, 130.9, 133.9, 134.6, 134.6, 136.8, 139.6, and 165.7.

HRMS calcd. for C_23_H_20_Cl_2_NOS^+^ ([M+H]^+^): 428.0637. Found: 428.0634.

*S*-((1R*,2R*,3R*,4S*)-3-((S*)-(2-chloroacetamido)(phenyl)methyl)bicyclo[2.2.1]hept-5-en-2-yl) ethanethioate (**22aa**)

Formed from thiazine **11aa** via *General method for the Staudinger ketene–imine cycloaddition of thiazines*. White solid yield as follows: 46%. R_f_ = 0.3 (*n*-hexane/EtOAc 10:1). Mp. 215–216 °C.

^1^H NMR (500 MHz, CDCl_3_): *δ* (ppm) = 1.40–1.47 (m, 1H, H-7), 1.54–1.60 (m, 1H, H-7), 2.16 (dd, *J* = 11.23 Hz, *J* = 7.91 Hz, 1H, H-3), 2.22–2.26 (m, 1H, H-4), 2.32 (s, 3H, CH_3_), 2.87–2.91 (m, 1H, H-1), 3.65 (dd, *J* = 7.63 Hz, *J* = 1.21 Hz, 1H, H-2), 3.86 (s, 2H, ClCH_2_), 4.65 (dd, *J* = 11.41 Hz, *J* = 7.50 Hz, 1H, N-CH), 6.08–6.13 (m, 1H, H-5), 6.15–6.20 (m, 1H, H-6), 6.70 (d, *J* = 6.72 Hz, 1H, NH), 7.26–7.30 (m, 3H, Ar), and 7.32–7.38 (m, 2H, Ar).

^13^C NMR (125 MHz, CDCl_3_): *δ* (ppm) = 30.6, 42.5, 44.9, 45.0, 45.1, 47.8, 51.1, 57.3, 127.1, 127.7, 128.7, 136.2, 139.3, 141.8, 164.6, and 196.1.

HRMS calcd. for C_18_H_21_ClNO_2_S^+^ ([M+H]^+^): 350.0976. Found: 350.0974.

*S*-((1R*,2R*,3R*,4S*)-3-((S*)-(2-chloroacetamido)(4-chlorophenyl)methyl)bicyclo[2.2.1]hept-5-en-2-yl) ethanethioate (**22ab**)

Formed from thiazine **11ab** via *General method for the Staudinger ketene–imine cycloaddition of thiazines*. White solid yield as follows: 25%. R_f_ = 0.75 (*n*-hexane/EtOAc 1:1). Mp. 175–178 °C.

^1^H NMR (500 MHz, CDCl_3_): *δ* (ppm) = 1.45 (d, *J* = 9.23 Hz, 1H, H-7), 1.53 (d, *J* = 9.22 Hz, 1H, H-7), 2.12 (dd, *J* = 11.33 Hz, *J* = 7.86 Hz, 1H, H-3), 2.20–2.25 (m, 1H, H-4), 2.32 (s, 3H, CH_3_), 2.87–2.93 (m, 1H, H-1), 3.61–3.67 (m, 1H, H-2), 3.85 (s, 2H, ClCH_2_), 4.60 (dd, *J* = 11.47 Hz, *J* = 7.09 Hz, 1H, N-CH), 6.08–6.14 (m, 1H, H-5), 6.16–6.21 (m, 1H, H-6), 6.69 (d, *J* = 6.55 Hz, 1H, NH), and 7.21 (d, *J* = 8.21 Hz, 2H, Ar), 7.32 (d, *J* = 8.23 Hz, 2H, Ar).

^13^C NMR (125 MHz, CDCl_3_): *δ* (ppm) = 30.6, 42.4, 44.9, 44.9, 45.1, 47.7, 51.0, 56.9, 128.5, 128.9, 133.5, 136.3, 139.2, 140.4, 164.8, and 196.1.

HRMS calcd. for C_18_H_20_Cl_2_NO_2_S^+^ ([M+H]^+^): 384.0586. Found: 384.0585.

(5S*,5aR*,6S*,9R*,9aR*)-3,5-diphenyl-4,5,5a,6,9,9a-hexahydro-6,9-methanobenzo[*f*][1,4]thiazepine-2-carboxylic acid methyl ester (**23ba**)

Formed from β-lactam **21ba** via *General method for the ring expansion of β-lactam condensed thiazinanes*. (eluent of column chromatography: *n*-hexane/EtOAc 10:1). Yellow solid yield as follows: 60%. R_f_ = 0.2 (*n*-hexane/EtOAc 10:1). Mp. 121–122 °C.

^1^H NMR (500 MHz, CDCl_3_): *δ* (ppm) = 1.24–1.29 (m, 1H, CH_2_), 1.88–1.93 (m, 1H, CH_2_), 2.13–2.17 (m, 1H, H-6), 2.20–2.27 (m, 1H, H-5a), 2.96–3.00 (m, 1H, H-9), 3.44 (s, 3H, OCH_3_), 3.68–3.73 (m, 1H, H-9a), 4.16 (brs, 1H, NH), 6.04–6.10 (m, 2H, H-5 and H-7), 6.12–6.16 (m, 1H, H-8), 7.20–7.25 (m, 2H, Ar), 7.27–7.33 (m, 3H, Ar), 7.33–7.37 (m, 3H, Ar), and 7.37–7.43 (m, 2H, Ar).

^13^C NMR (125 MHz, CDCl_3_): *δ* (ppm) = 42.8, 45.5, 47.7, 49.1, 50.2, 51.4, 61.8, 89.9, 127.4, 127.5, 128.2, 128.6, 128.7, 129.4, 135.4, 138.1, 140.9, 141.7, 157.1, and 168.4.

HRMS calcd. for C_24_H_24_NO_2_S^+^ ([M+H]^+^): 390.1522. Found: 390.1520.

(5S*,5aR*,6S*,9R*,9aR*)-5-(4-chlorophenyl)-3-phenyl-4,5,5a,6,9,9a-hexahydro-6,9-methanobenzo[*f*][1,4]thiazepine-2-carboxylic acid methyl ester (**23bb**)

Formed from β-lactam **21bb** via *General method for the ring expansion of β-lactam condensed thiazinanes* (eluent of column chromatography: *n*-hexane/EtOAc 10:1). Yellow solid yield as follows: 62%. R_f_ = 0.2 (*n*-hexane/EtOAc 10:1). Mp. 170–172 °C.

^1^H NMR (500 MHz, CDCl_3_): *δ* (ppm) = 1.25–1.30 (m, 1H, CH_2_), 1.86–1.91 (m, 1H, CH_2_), 2.12–2.16 (m, 1H, H-6), 2.19 (dd, *J* = 10.94 Hz, *J* = 7.16 Hz, 1H, H-5a), 2.96–3.00 (m, 1H, H-9), 3.43 (s, 3H, OCH_3_), 3.70 (d, *J* = 6.95 Hz, 1H, H-9a), 4.02 (broad doublet, *J* = 3.57 Hz, 1H, NH), 6.03–6.06 (m, 1H, H-5), 6.06–6.10 (m, 1H, H-7), 6.12–6.16 (m, 1H, H-8), 7.19–7.23 (m, 2H, Ar), 7.27–7.34 (m, 5H, Ar), and 7.35–7.41 (m, 2H, Ar).

^13^C NMR (125 MHz, CDCl_3_): *δ* (ppm) = 42.8, 45.4, 47.7, 49.1, 50.2, 51.4, 61.2, 90.4, 127.5, 128.3, 128.8, 128.9, 129.6, 134.4, 135.5, 138.0, 140.1, 140.7, 156.7, and 168.3.

HRMS calcd. for C_24_H_23_ClNO_2_S^+^ ([M+H]^+^): 424.1133. Found: 424.1129.

## 4. Conclusions

From readily available reagents, various *N*,*S*-heterocycles were prepared in a short synthetic pathway (1–3 steps). First, a three-component reaction of a cycloalkene, a thioamide, and an aldehyde provided 5,6-dihydro-4*H*-1,3-thiazines. Afterwards, Staudinger ketene–imine cycloaddition with chloroketene resulted in β-lactam-fused 1,3-thiazinanes. Finally, treatment with methoxide resulted in ring expansion, yielding 4,5,6,7-tetrahydro-1,4-thiazepines. Although the synthetic pathway generated 3–5 new chiral centers with the help of pericyclic reactions, almost every cycloaddition proceeded in a diastereoselective manner, and 2D NOESY as well as single-crystal X-ray diffraction enabled unequivocal determination of the stereochemistry of all synthesized compounds.

Based on our results and the findings of Peudru et al., the initial three-component reaction is applicable to various thioamides, aromatic aldehydes, and olefins. Therefore, in our opinion, the method described in this paper can be useful for the synthesis of a variety of *N*,*S*-heterocycles.

## Figures and Tables

**Table 1:** Hetero-Diels–Alder reactions of norbornadiene and *N*-thioacyl imines.

Product	R^1^	R^2^	Yields (*Method A*)	Yields (*Method B*)
**11aa**	Me	Ph	55%	45%
**11ab**	Me	4-Cl-Ph	56%	–
**11ba**	Ph	Ph	58%	50%
**11bb**	Ph	4-Cl-Ph	36%	28%

**Table 2:** Reactions of compounds **16** with chloroketene.

Substrate	R^1^	R^2^	Yield of β-Lactam	Yield of Thioester
**11aa**	Me	Ph	not formed	46% **22aa**
**11ab**	Me	4-Cl-Ph	12% **21ab**	25% **22ab**
**11ba**	Ph	Ph	58% **21ba**	not formed
**11bb**	Ph	4-Cl-Ph	56% **21bb**	not formed

## Data Availability

The original contributions presented in this study are included in the article and Supplementary Materials. Further inquiries can be directed to the corresponding author.

## Supplementary Materials

The following supporting information can be downloaded at: https://www.mdpi.com/article/10.3390/ijms262311543/s1, SuppInfo_N_thioacyl_imines.docx (Experimental section, Characterization data, X-ray structure determinations, Copies of NMR spectra).

## References

[1] Eicher T., Hauptmann S., Speicher A. (2012). The Chemistry of Heterocycles: Structure, Reactions, Synthesis, and Applications.

[2] Aniszewski T. (2015). Alkaloids: Chemistry, Biology, Ecology, and Applications.

[3] Vitaku E., Smith D.T., Njardarson J.T. (2014). Analysis of the Structural Diversity, Substitution Patterns, and Frequency of Nitrogen Heterocycles among U.S. FDA Approved Pharmaceuticals. J. Med. Chem..

[4] Marshall C.M., Federice J.G., Bell C.N., Cox P.B., Njardarson J.T. (2024). An Update on the Nitrogen Heterocycle Compositions and Properties of U.S. FDA-Approved Pharmaceuticals (2013–2023). J. Med. Chem..

[5] Gomtsyan A. (2012). Heterocycles in drugs and drug discovery. Chem. Heterocycl. Comp..

[6] Mora-Ochomogo M., Lohans C.T. (2021). β-Lactam antibiotic targets and resistance mechanisms: From covalent inhibitors to substrates. RSC Med. Chem..

[7] Śnieżek M., Stecko S., Panfil I., Furman B., Chmielewski M. (2013). Total Synthesis of Ezetimibe, a Cholesterol Absorption Inhibitor. J. Org. Chem..

[8] Scott K.A., Njardarson J.T. (2018). Analysis of US FDA-Approved Drugs Containing Sulfur Atoms. Top Curr. Chem. (Z).

[9] Kapoor K., Kaur N., Sohal H.S., Kaur M., Singh K., Kumar A. (2025). Drugs and Their Mode of Action: A Review on Sulfur-Containing Heterocyclic Compounds. Polycycl. Aromat. Compd..

[10] Ruiz-Colón K., Chavez-Arias C., Díaz-Alcalá J.E., Martínez M.A. (2014). Xylazine intoxication in humans and its importance as an emerging adulterant in abused drugs: A comprehensive review of the literature. Forensic Sci. Int..

[11] Koketsu M., Tanaka K., Takenaka Y., Kwong C.D., Ishihara H. (2002). Synthesis of 1,3-thiazine derivatives and their evaluation as potential antimycobacterial agents. Eur. J. Pharm. Sci..

[12] Matysiak J. (2006). Synthesis, antiproliferative and antifungal activities of some 2-(2,4-dihydroxyphenyl)-4*H*-3,1-benzothiazines. Bioorg. Med. Chem..

[13] Niewiadomy A., Matysiak J., Karpińska M.M. (2011). Synthesis and Anticancer Activity of New 2-Aryl-4H-3,1-benzothiazines. Arch. Pharm. Chem. Life Sci..

[14] Güschow M., Schlenk M., Gäb J., Paskaleva M., Alnouri M.W., Scolari S., Iqbal J., Müller C.E. (2012). Benzothiazinones: A Novel Class of Adenosine Receptor Antagonists Structurally Unrelated to Xanthine and Adenine Derivatives. J. Med. Chem..

[15] Li J., Fan X., Deng J., Liang Y., Ma S., Lu Y., Zhang J., Shi T., Tan W., Wang Z. (2020). Design and synthesis of 1,3-benzothiazinone derivatives as potential anti-inflammatory agents. Bioorg. Med. Chem..

[16] Zhang P., Min Z., Gao Y., Bian J., Lin X., He J., Ye D., Li Y., Peng C., Cheng Y. (2021). Discovery of Novel Benzothiazepinones as Irreversible Covalent Glycogen Synthase Kinase 3β Inhibitors for the Treatment of Acute Promyelocytic Leukemia. J. Med. Chem..

[17] Nicolau S.M., de Diego A.M.G., Cortés L., Egea J., González J.C., Mosquera M., López M.G., Hernández-Guijo J.M., García A.G. (2009). Mitochondrial Na^+^/Ca^2+^-exchanger blocker CGP37157 protects against chromaffin cell death elicited by veratridine. J. Pharmacol. Exp. Ther..

[18] Mitronova G.Y., Quentin C., Belov V.N., Wegener J.W., Kiszka K.A., Lehnart S.E. (2023). 1,4-Benzothiazepines with Cyclopropanol Groups and Their Structural Analogues Exhibit Both RyR2-Stabilizing and SERCA2a-Stimulating Activities. J. Med. Chem..

[19] Kaneko N., Matsuda R., Hata Y., Shimamoto K. (2009). Pharmacological characteristics and clinical applications of K201. Curr. Clin. Pharmacol..

[20] Wong B.S., Camilleri M. (2013). Elobixibat for the treatment of constipation. Expert Opin. Investig. Drugs.

[21] Magdalenić K., Ronse U., De Jonghe S., Persoons L., Schols D., De Munck J., Grootaert C., Van Camp J., D’hooghe M. (2023). Synthesis and cancer cell cytotoxicity of 2-aryl-4-(4-aryl-2-oxobut-3-en-1-ylidene)-substituted benzothiazepanes. Phytochem. Lett..

[22] Kaneko N., Loughrey C.M., Smith G., Matsuda R., Hasunuma T., Mark P.B., Toda M., Shinozaki M., Otani N., Kayley S. (2024). A novel ryanodine receptor 2 inhibitor, M201-A, enhances natriuresis, renal function and lusi-inotropic actions: Preclinical and phase I study. Br. J. Pharmacol..

[23] Free R.B., Nilson A.N., Boldizsar N.M., Doyle T.B., Rodriguiz R.M., Pogorelov V.M., Machino M., Lee K.H., Bertz J.W., Xu J. (2023). Identification and Characterization of ML321: A Novel and Highly Selective D_2_ Dopamine Receptor Antagonist with Efficacy in Animal Models That Predict Atypical Antipsychotic Activity. ACS Pharmacol. Transl. Sci..

[24] Barcan G.A., Kwon D.-H., Guo J., Kowalski J.A., Liu L., Nilson M., Sisko J., Wang H., Brown T.A., Gholipour-Ranjbar H. (2023). A Sulfur-Controlled Approach to the Synthesis of Linerixibat. J. Org. Chem..

[25] Zheng X., Gao L., Wang L., Liang C., Wang B., Liu Y., Feng S., Zhang B., Zhou M., Yu X. (2019). Discovery of Ziresovir as a Potent, Selective, and Orally Bioavailable Respiratory Syncytial Virus Fusion Protein Inhibitor. J. Med. Chem..

[26] Huang Q., Cai D., Yan R., Li L., Zong Y., Guo L., Mercier A., Zhou Y., Tang A., Henne K. (2020). Preclinical Profile and Characterization of the Hepatitis B Virus Core Protein Inhibitor ABI-H0731. Antimicrob. Agents Chemother..

[27] Harris P.A., Berger S.B., Jeong J.U., Nagilla R., Bandyopadhyay D., Campobasso N., Capriotti C.A., Cox J.A., Dare L., Dong X. (2017). Discovery of a First-in-Class Receptor Interacting Protein 1 (RIP1) Kinase Specific Clinical Candidate (GSK2982772) for the Treatment of Inflammatory Diseases. J. Med. Chem..

[28] Kent C.N., Fulton M.G., Stillwell K.J., Dickerson J.W., Loch M.T., Rodriguez A.L., Blobaum A.L., Boutaud O., Rook J.L., Niswender C.M. (2021). Discovery and optimization of a novel CNS penetrant series of mGlu_4_ PAMs based on a 1,4-thiazepane core with in vivo efficacy in a preclinical Parkinsonian model. Bioorg. Med. Chem. Lett..

[29] Fodor L., Szabó J. (1981). Cycloaddition reactions of 1,3-benzothiazines–I. Reactions of 2-phenyl-4-H and 4-phenyl-2H-1,3-benzothiazine derivatives with substituted acetyl chlorides. Tetrahedron.

[30] Fodor L., Szabó J., Bernáth G. (1981). Synthesis of 4,5-dihydro-1,4-benzothiazepine derivatives via ring expansion. Tetrahedron Lett..

[31] Fodor L., Szabó J., Szűcs E., Bernáth G., Sohár P., Tamás J. (1984). Ring transformations of 1,3-benzothiazine derivatives II. Conversion of 6α-aryl-7α-chloro-2,3(2′,3′-dialkoxybenzo)-1-thiaoctems into 2-carbomethoxy-3-aryl-7,8-dialkoxy-4,5-dihydro-1,4-benzothiazepines. Tetrahedron.

[32] Peudru F., Lohier J.-F., Gulea M., Reboul V. (2016). Synthesis of 1,3-thiazines by a three-component reaction and their transformations into β-lactam-condensed 1,3-thiazine and 1,4-thiazepine derivatives. Phosphorus Sulfur Silicon Relat. Elem..

[33] Weichert A., Hoffman H.M.R. (1991). Synthesis and Reactions of α-Methylene-β-keto Sulfones. J. Org. Chem..

[34] Hsiao C.-C., Raja S., Liao H.-H., Atodiresei I., Rueping M. (2015). *Ortho*-Quinone Methides as Reactive Intermediates in Asymmetric Brønsted Acid Catalyzed Cycloadditions with Unactivated Alkenes by Exclusive Activation of the Electrophile. Angew. Chem. Int. Ed..

[35] Edwards W.D., Du M., Royal J.S., McHale J.L. (1990). Theoretical Study of the Charge-Transfer Spectrum of the Indene-Tetracyanoethylene Electron Donor-Acceptor Complex. J. Phys. Chem..

[36] Hanessian S., Compain P. (2002). Lewis acid promoted cyclocondensations of α-ketophosphonoenoates with dienes—From Diels–Alder to hetero Diels–Alder reactions. Tetrahedron.

[37] Clayden J., Greeves N., Warren S., Wothers P. (2009). Organic Chemistry.

[38] Sohár P., Szabó J., Simon L., Talpas G.S., Szűcs E., Bernáth G. (1991). Synthesis and Steric Structure of Alicycle-Fused 1,3-Thiazine-β-lactams. Magn. Reson. Chem..

[39] (2015). CrysAlisPro.

[40] (2023). CrysAlisPro.

[41] Sheldrick G.M. (2015). SHELXT—Integrated space-group and crystal-structure determination. Acta Crystallogr. A.

[42] Sheldrick G.M. (2015). Crystal structure refinement with *SHELXL*. Acta Crystallogr. C.

[43] Hübschle C.B., Sheldrick G.M., Dittrich B. (2011). *ShelXle*: A Qt graphical user interface for *SHELXL*. J. Appl. Cryst..

