# Supplementary material for: Synthesis of 1,3-Thiazine and 1,4-Thiazepine Derivatives via Cycloadditions and Ring Expansion
Source: Int J Mol Sci. 2025 Nov 28;26(23):11543. doi: 10.3390/ijms262311543 (PMC12692644; doi:10.3390/ijms262311543)

Supporting Information

Synthesis of 1,3-thiazine and 1,4-thiazepine derivatives via cycloadditions and ring expansion

M. Palkó[1], N. Becker[1], E. Wéber[2,3], M. Haukka[4], A. M. Remete[1]*

[1]*Institute of Pharmaceutical Chemistry, University of Szeged, H-6720 Szeged, Eötvös u. 6, Hungary;* palko.marta@szte.hu; beckernori@gmail.com

[2]*Department of Medical Chemistry, University of Szeged, H-6720 Szeged, Dóm tér 8, Hungary;* weber.edit@med.u-szeged.hu

[3]*HUN-REN-SZTE Biomimetic Systems Research Group, Dóm tér 8, H-6720, Szeged, Hungary;* weber.edit@med.u-szeged.hu

[4]*Department of Chemistry, University of Jyväskylä, FIN-40014, Jyväskylä, Finland;* matti.o.haukka@jyu.fi

Correspondance: remete.attila.mario@szte.hu

Table of contents

1. Experimental section 2

1.1. General information 2

1.2. Synthetic procedures 2

2. Characterization data 4

3. X-ray structure determinations 15

4. Copies of NMR spectra 19

1. Experimental section

1.1. General information

Chemicals were purchased from Sigma–Aldrich and Acros Organics. Solvents were used as received from the suppliers. Melting points were determined with a Kofler apparatus. TLC plates (TLC Silica gel 60 F_{254}) and silica gel for column chromatography (technical grade, pore size 60 Å, 70-230 mesh) were purchased from Merck. NMR spectra were acquired at room temperature on a Bruker Avance 400 spectrometer with 9.39 T magnetic field strength (^{1}H frequency: 400.13 MHz, ^{13}C frequency: 100.76 MHz, respectively), on a Bruker Avance Neo 500 spectrometer with 11.75 T magnetic field strength (^{1}H frequency 500.20 MHz, ^{13}C frequency 125.78 MHz), or on a Bruker Avance III 600 spectrometer with 14.10 T magnetic field (^{1}H frequency 600.20 MHz, ^{13}C frequency 150.92 MHz) in $CDCl_3$ or D_6-DMSO, using the deuterium signal of the solvent to lock the field. The ^{1}H and ^{13}C chemical shifts are given relative to TMS. HRMS were acquired on a Thermo Scientific Q-Exactive Plus Orbitrap mass spectrometer (Thermo Fisher Scientific Inc., Budapest, Hungary) equipped with an electrospray ionization ion source. Single crystal data of compounds **11ba**, **13aa**, and **21bb** was collected on a SuperNova, Dualflex, HyPix-Arc 100 diffractometer at 120(2) K. The crystal of **11ba** was solved in orthorhombic space group Pbca. The unit cell parameters: a = 11.7009(2) Å, b = 15.6961(3) Å, c = 17.6878(4) Å, V = 3248.53(10) Å^3. The crystal of **13aa** was solved in monoclinic space group $P2_1/n$. The unit cell parameters: a = 8.25120(7) Å, b = 16.50920(13) Å, c = 21.02664(18) Å, β = 94.1989(7)°, V = 2856.58(4) Å^3. The crystal of **21bb** was solved in monoclinic space group $P2_1/c$. The unit cell parameters: a = 13.43125(18) Å, b = 6.82967(9) Å, c = 21.6001(3) Å, β = 98.5980(12)°, V = 1959.13(4) Å^3. The crystallographic parameters are given in Section 3 ('X-ray structure determinations') of the supporting material.

1.2. Synthetic procedures

General methods for the synthesis of thiazines

Method A: In a 10 mL microwave reaction vial, 1.2 mmol thioamide, 1 mmol aldehyde, and 1.2 mmol cyclic olefin were dissolved in 10 mL 1,2-dichloroethane. To this mixture, 2 mmol (0.25 ml) boron trifluoride etherate ($BF_3 \times OEt_2$) was added dropwise at RT. After that, the vial was sealed and irradiated at 40 W power in a microwave reactor for 10 minutes (T ≤ 150 °C) with stirring. After cooling to room temperature, the reaction mixture was washed with 10 ml

saturated aqueous $NaHCO_3$ solution, then the aqueous phase was extracted with 3×15 mL dichloromethane. The combined organic phase was dried on Na_2SO_4. After the drying agent was filtered out, the resulting filtrate was evaporated and purified via column chromatography on silica gel. Sometimes, after column chromatography, recrystallization was also necessary to obtain a pure product.

Method B: Similar to Method A, but CH_2Cl_2 was used as a solvent, and the reaction mixture was stirred at RT for 24 hours (microwave irradiation was omitted).

General method for the Staudinger ketene–imine cycloaddition of thiazines

In a three-necked round-bottom flask, 3.0 mmol thiazine was dissolved in 30 ml anhydrous toluene. The stirred reaction mixture was heated to reflux. From two dropping funnels, a chloroacetyl chloride solution (1.0 mmol $ClCH_2COCl$ dissolved in 10 ml anhydrous toluene) and a triethylamine solution (1.0 mmol Et_3N dissolved in 10 ml anhydrous toluene) were added slowly (under about 1 hour) to the reaction mixture under reflux. The "reagent addition step" described in the previous sentence was carried out three times. Then, reflux was continued until the overall reaction time reached 4 hours. After that, the reaction mixture was evaporated and purified via column chromatography on silica gel (eluent: *n*-hexane/ EtOAc 10:1). The raw product was purified further via recrystallization.

General method for the ring expansion of β-lactam condensed thiazinanes

0.16 mmol β-lactam condensed 1,3-thiazinane and 2 equiv NaOMe were dissolved in 2 ml methanol. Under an argon atmosphere, the reaction mixture was treated under reflux for 4 hours. Afterwards, the reaction mixture was concentrated under reduced pressure. The residue was dissolved in 4 ml water and extracted with 4×10 ml CH_2Cl_2. The organic phase was dried on Na_2SO_4. After the drying agent was filtered out, the resulting filtrate was evaporated and purified via column chromatography on silica gel and subsequent recrystallization.

2. Characterization data

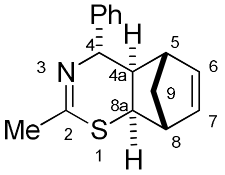

(4*S,4a*R**,5*S**,8*R**,8a*R**)-2-methyl-4-phenyl-4a,5,8,8a-tetrahydro-4*H*-5,8-methanobenzo[*e*][1,3]thiazine (11aa)**

Formed diastereoselectively from thioacetamide (**8a**), benzaldehyde (**9a**), and norbornadiene (**10**) via *General methods for the synthesis of thiazines*. Purification: column chromatography (eluent: *n*-hexane/EtOAc 4:1) followed by crystallization. White powder, yield: 55% (*Method A*) or 45% (*Method B*). R_f = 0.6 (*n*-hexane/EtOAc 4:1). Mp. 88-89 °C.

^{1}H NMR (500 MHz, $CDCl_3$): δ (ppm) = 1.52-1.58 (m, 1H, H-9), 1.83-1.91 (m, 1H, H-4a), 2.32 (s, 3H, CH_3), 2.37-2.43 (m, 1H, H-9), 2.58-2.62 (m, 1H, H-5), 2.86-2.90 (m, 1H, H-8), 3.05-3.09 (m, 1H, H-8a), 3.77-3.83 (m, 1H, H-4), 6.02-6.06 (m, 1H, H-7), 6.06-6.11 (m, 1H, H-6), 7.27-7.33 (m, 1H, Ar), 7.35-7.42 (m, 4H, Ar).

^{13}C NMR (125 MHz, $CDCl_3$): δ (ppm) = 28.9, 43.3, 46.0, 46.2, 48.8, 50.5, 70.4, 127.1, 128.3, 128.5, 135.5, 139.4, 144.2, 165.2.

HRMS calcd. for $C_{16}H_{18}NS^+$ ($[M+H]^+$): 256.1154. Found: 256.1150.

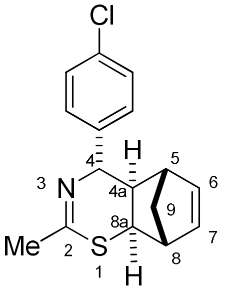

(4*S,4a*R**,5*S**,8*R**,8a*R**)-2-methyl-4-(4-chlorophenyl)-4a,5,8,8a-tetrahydro-4*H*-5,8-methanobenzo[*e*][1,3]thiazine (11ab)**

Formed diastereoselectively from thioacetamide (**8a**), 4-chlorobenzaldehyde (**14b**), and norbornadiene (**10**) via *General methods for the synthesis of thiazines*. Purification: column chromatography (eluent: *n*-hexane/EtOAc 4:1). white solid, yield: 56% (*Method A*). R_f = 0.5 (*n*-hexane/EtOAc 4:1). Mp. 126-128 °C.

^{1}H NMR (400 MHz, $CDCl_3$): δ (ppm) = 1.52-1.59 (m, 1H, H-9), 1.77-1.86 (m, 1H, H-4a), 2.32 (d, J = 1.67 Hz, 3H, CH_3), 2.34-2.40 (m, 1H, H-9), 2.53-2.59 (m, 1H, H-5), 2.86-2.92 (m, 1H, H-8), 3.03-3.10 (m, 1H, H-8a), 3.74-3.82 (m, 1H, H-4), 6.02-6.06 (m, 1H, H-7), 6.06-6.12 (m, 1H, H-6), 7.29-7.34 (m, 2H, Ar), 7.34-7.40 (m, 2H, Ar).

^{13}C NMR (125 MHz, $CDCl_3$): δ (ppm) = 28.8, 43.2, 45.9, 46.1, 48.8, 50.6, 69.7, 128.7, 129.7, 132.9, 135.6, 139.2, 142.7, 165.7.
HRMS calcd. for $C_{16}H_{17}ClNS^+$ ($[M+H]^+$): 290.0765. Found: 290.0762.

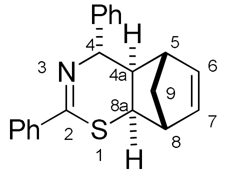

(4*S,4a*R**,5*S**,8*R**,8a*R**)-2,4-diphenyl-4a,5,8,8a-tetrahydro-4*H*-5,8-methanobenzo[*e*][1,3]thiazine (11ba)**

Formed diastereoselectively from thiobenzamide (**8b**), benzaldehyde (**9a**), and norbornadiene (**10**) via *General methods for the synthesis of thiazines*. Purification: column chromatography (eluent: *n*-hexane/EtOAc 4:1) followed by crystallization. White powder, yield: 58% (*Method A*) or 50% (*Method B*). R_f = 0.6 (*n*-hexane/EtOAc 4:1). Mp. 130-134 °C.
^{1}H NMR (500 MHz, $CDCl_3$): δ (ppm) = 1.63 (d, J = 9.05 Hz, 1H, H-9), 1.97-2.03 (m, 1H, H-4a), 2.54 (d, J = 9.04 Hz, 1H, H-9), 2.68-2.72 (m, 1H, H-5), 2.99-3.04 (m, 1H, H-8), 3.16 (d, J = 7.78 Hz, 1H, H-8a), 4.11 (d, J = 10.83 Hz, 1H, H-4), 6.05-6.10 (m, 1H, H-7), 6.10-6.14 (m, 1H, H-6), 7.30-7.39 (m, 3H, Ar), 7.39-7.45 (m, 3H, Ar), 7.47-7.53 (m, 2H, Ar), 7.98-8.04 (m, 2H, Ar).
^{13}C NMR (125 MHz, $CDCl_3$): δ (ppm) = 43.4, 46.1, 47.2, 48.9, 52.6, 71.4, 127.1, 127.6, 128.3, 128.4, 128.6, 130.8, 135.6, 138.3, 139.7, 144.4, 165.6.
HRMS calcd. for $C_{21}H_{20}NS^+$ ($[M+H]^+$): 318.1311. Found: 318.1305.

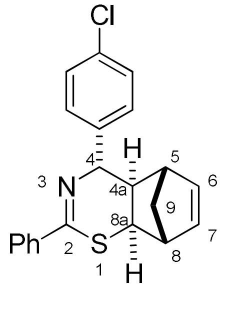

(4*S,4a*R**,5*S**,8*R**,8a*R**)-4-(4-chlorophenyl)-2-phenyl-4a,5,8,8a-tetrahydro-4*H*-5,8-methanobenzo[*e*][1,3]thiazine (11bb)**

Formed diastereoselectively from thiobenzamide (**8b**), 4-chlorobenzaldehyde (**14b**), and norbornadiene (**10**) via *General methods for the synthesis of thiazines*. Purification: column chromatography (eluent: *n*-hexane/EtOAc 20:1) followed by a second column chromatography (eluent: *n*-hexane/PhMe 3:1). White oil, yield: 36% (*Method A*) or 28% (*Method B*). R_f = 0.6 (*n*-hexane/EtOAc 20:1).

^{1}H NMR (500 MHz, $CDCl_3$): δ (ppm) = 1.63 (d, J = 9.09 Hz, 1H, H-9), 1.91-1.98 (m, 1H, H-4a), 2.51 (d, J = 9.08 Hz, 1H, H-9), 2.64-2.69 (m, 1H, H-5), 2.99-3.04 (m, 1H, H-8), 3.13-3.18 (m, 1H, H-8a), 4.09 (d, J = 10.89 Hz, 1H, H-4), 6.06-6.10 (m, 1H, H-7), 6.10-6.15 (m, 1H, H-6), 7.34-7.41 (m, 4H, Ar), 7.41-7.46 (m, 3H, Ar), 7.97-8.02 (m, 2H, Ar).

^{13}C NMR (125 MHz, $CDCl_3$): δ (ppm) = 43.4, 46.1, 47.1, 48.8, 52.7, 70.7, 127.5, 128.3, 128.5, 129.9, 130.9, 132.9, 135.7, 138.1, 139.5, 142.9, 166.0.

HRMS calcd. for $C_{21}H_{19}ClNS^+$ ($[M+H]^+$): 352.8997. Found: 352.0917.

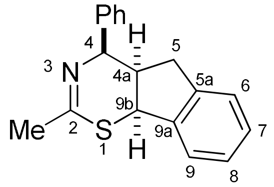

(4*R,4a*R**,9b*S**)-4-phenyl-2-methyl-4,4a,5,9b-tetrahydroindeno[2,1-*e*][1,3]thiazine (13aa)**

Formed regio- and diastereoselectively from thioacetamide (**8a**), benzaldehyde (**9a**), and indene (**12**) via *General methods for the synthesis of thiazines*. Purification: column chromatography (eluent: *n*-hexane/EtOAc 4:1) followed by crystallization. White solid, yield: 57% (*Method B*). R_f = 0.4 (*n*-hexane/EtOAc 4:1). Mp. 124 °C.

^{1}H NMR (500 MHz, $CDCl_3$): δ (ppm) = 2.25 (d, J = 1.91 Hz, 3H, CH_3), 2.39 (dd, J = 15.95 Hz, J = 7.53 Hz, 1H, H-5), 2.89 (dd, J = 15.89 Hz, J = 10.56 Hz, 1H, H-5), 3.18-3.26 (m 1H, H-4a), 4.60-4.65 (m, 1H, H-4), 4.98 (d, J = 8.13 Hz, 1H, H-9b), 7.04-7.51 ppm (9H, m, Ar).

^{13}C NMR (125 MHz, $CDCl_3$) δ (ppm) = 28.6, 30.4, 44.1, 49.4, 62.7, 124.2, 125.3, 126.7, 127.0, 127.2, 128.1, 128.5, 142.0, 143.7, 144.5, 163.0.

HRMS calcd. for $C_{18}H_{18}NS^+$ ($[M+H]^+$): 280.1154. Found: 280.1150.

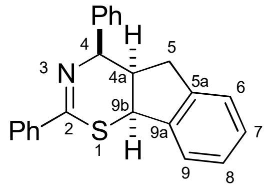

(4*R,4a*R**,9b*S**)-2,4-diphenyl-4,4a,5,9b-tetrahydroindeno[2,1-*e*][1,3]thiazine (13ba)**

Formed regio- and diastereoselectively from thiobenzamide (**8b**), benzaldehyde (**9a**), and indene (**12**) via *General methods for the synthesis of thiazines*. Purification: column chromatography (eluent: *n*-hexane/EtOAc 4:1) followed by crystallization. White solid, yield: 57% (*Method B*). R_f = 0.7 (*n*-hexane/EtOAc 4:1). Mp. 141-142 °C.

^{1}H NMR (500 MHz, D_6-DMSO): δ (ppm) = 2.42 (dd, J = 16.25 Hz, J = 7.87 Hz, 1H, H-5), 2.91 (dd, J = 15.95 Hz, J = 10.06 Hz, 1H, H-5), 3.55-3.63 (m, 1H, H-4a), 4.99-5.11 (m, 1H, H-4), 5.40 (d, J = 8.23 Hz, 1H, H-9b), 7.09-7.16 (m ,1H, Ar), 7.16-7.24 (m, 2H, Ar), 7.33-7.40 (m, 1H, Ar), 7.41-7.51 (m, 5H, Ar), 7.52-7.59 (m, 1H, Ar), 7.64-7.70 (m, 2H, Ar), 7.80-7.85 (m, 2H, Ar).
^{13}C NMR (125 MHz, $CDCl_3$): δ (ppm) = 30.8, 45.5, 50.4, 64.2, 124.6, 125.1, 126.7, 127.0, 127.4, 128.1, 128.2, 128.4, 130.6, 139.0, 142.0, 143.7, 144.5, 164.0 ppm.
HRMS calcd. for $C_{23}H_{20}NS^+$ ($[M+H]^+$): 342.1311. Found: 342.1306.

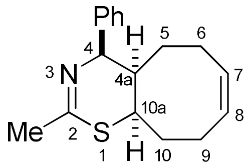

(4*R,4a*R**,10a*R**,*Z*)-2-methyl-4-phenyl-4a,5,6,9,10,10a-hexahydro-4*H*-cycloocta[*e*][1,3]thiazine (15aa)**

Formed from thioacetamide (**8a**), benzaldehyde (**9a**), and 1,5-cyclooctadiene (**14**) via *General methods for the synthesis of thiazines*. Purification: column chromatography (eluent: *n*-hexane/EtOAc 14:1) followed by crystallization, white solid, yield: 52% (*Method B*). R_f = 0.3 (*n*-hexane/EtOAc 14:1). Mp. 137-138 °C.
^{1}H NMR (500 MHz, $CDCl_3$): δ (ppm) = 0.92-1.03 (m, 1H, H-5), 1.51-1.62 (m, 1H, H-5), 1.73-1.88 (m, 2H, H-6 and H-10), 2.18-2.34 (m, 5H, CH_3 and H-9 and H-10), 2.45-2.58 (m, 2H, H-4a and H-9), 2.58-2.69 (m, 1H, H-6), 3.97-4.04 (m, 1H, H-10a), 4.63-4.68 (m, 1H, H-4), 5.28-5.37 (m, 1H, H-7), 5.56-5.64 (m, 1H, H-8), 7.19-7.25 (m, 1H, Ar), 7.30-7.40 (m, 4H, Ar).
^{13}C NMR (125 MHz, $CDCl_3$): δ (ppm) = 20.3, 23.8, 27.7, 28.8, 30.8, 34.1, 48.2, 66.6, 125.9, 126.1, 127.3, 128.1, 130.9, 143.6, 158.9.
HRMS calcd. for $C_{17}H_{22}NS^+$ ($[M+H]^+$): 272.1467. Found: 272.1463.

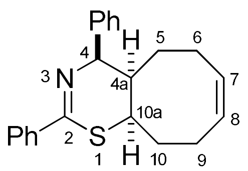

(4*R,4a*R**,10a*R**,*Z*)-2,4-diphenyl-4a,5,6,9,10,10a-hexahydro-4*H*-cycloocta[*e*][1,3]thiazine (15ba)**

Formed from thiobenzamide (**8b**), benzaldehyde (**9a**), and 1,5-cyclooctadiene (**14**) via *General methods for the synthesis of thiazines*. Purification: column chromatography (eluent: *n*-hexane/EtOAc 4:1), then a second round of column chromatography (eluent:

n-hexane/EtOAc 14:1), and finally crystallization. White solid, yield: 32% (*Method B*). R_f = 0.5 (*n*-hexane/EtOAc 14:1). Mp. 69-72 °C.

^{1}H NMR (500 MHz, $CDCl_3$): δ (ppm) = 0.95-1.06 (m, 1H, H-5), 1.57-1.67 (m, 1H, H-5), 1.72-1.82 (m, 1H, H-6), 1.92-2.02 (m, 1H, H-10), 2.26-2.45 (m, 2H, H-9, H-10), 2.54-2.72 (m, 3H, H-4a, H-6, H-9), 4.09-4.16 (m, 1H, H-10a), 4.91 (d, *J* = 3.34 Hz, 1H, H-4), 5.30-5.41 (m, 1H, H-7), 5.59-5.68 (m, 1H, H-8), 7.23-7.26 (m, 1H, Ar), 7.34-7.47 (m, 5H, Ar), 7.47-7.52 (m, 2H, Ar), 7.95-8.00 (m, 2H, Ar).

^{13}C NMR (125 MHz, $CDCl_3$): δ (ppm) = 20.7, 23.8, 28.8, 31.1, 34.2, 48.3, 66.8, 126.0, 126.4, 126.4, 127.3, 128.1, 128.3, 130.4, 130.9, 138.9, 143.6, 159.2.

HRMS calcd. for $C_{22}H_{24}NS^+$ ($[M+H]^+$): 334.1624. Found: 334.1621.

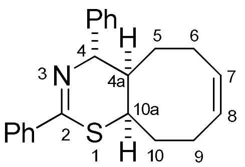

(4*S,4a*R**,10a*R**,*Z*)-2,4-diphenyl-4a,5,6,9,10,10a-hexahydro-4*H*-cycloocta[*e*][1,3]thiazine (16ba)**

Formed from thiobenzamide (**8b**), benzaldehyde (**9a**), and 1,5-cyclooctadiene (**14**) via *General methods for the synthesis of thiazines*. Purification: column chromatography (eluent: *n*-hexane/EtOAc 4:1), then a second round of column chromatography (eluent: *n*-hexane/EtOAc 14:1), and finally crystallization. White solid, yield: 22% (*Method B*). R_f = 0.3 (*n*-hexane/EtOAc 14:1). Mp. 116-118 °C.

^{1}H NMR (500 MHz, $CDCl_3$): δ (ppm) = 1.47-1.56 (m, 1H, H-5), 1.79-1.88 (m, 1H, H-10), 1.89-1.98 (m, 1H, H-9), 2.02-2.12 (m, 2H, H-5, H-6), 2.27-2.37 (m, 1H, H-10), 2.37-2.43 (m, 1H, H-4a), 2.48-2.57 (m, 1H, H-9), 2.67-2.79 (m, 1H, H-6), 3.38-3.47 (m, 1H, H-10a), 5.16 (d, *J* = 4.1 Hz, 1H, H-4), 5.53-5.67 (m, 2H, H-7, H-8), 7.16-7.21 (m, 2H, Ar), 7.22-7.27 (m, 1H, Ar), 7.30-7.36 (m, 2H, Ar), 7.37-7.46 (m, 3H, Ar), 7.90-7.94 (m, 2H, Ar).

^{13}C NMR (125 MHz, $CDCl_3$): δ (ppm) = 25.4, 27.4, 28.9, 30.6, 36.3, 39.4, 66.9, 126.5, 126.9, 127.0, 127.1, 128.4, 128.5, 129.8, 130.6, 139.0, 142.9.

HRMS calcd. for $C_{22}H_{24}NS^+$ ($[M+H]^+$): 334.1624. Found: 334.1620.

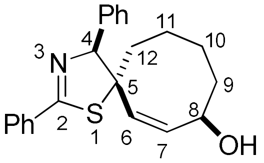

(4*S,5*R**,8*R**,*Z*)-2,4-diphenyl-1-thia-3-azaspiro[4.7]dodeca-2,6-dien-8-ol (18)**

Formed from thiobenzamide (**8b**), benzaldehyde (**9a**), and 1,3-cyclooctadiene (**17**) via *General methods for the synthesis of thiazines*. Purification: column chromatography (eluent: *n*-hexane/EtOAc 7:2), then crystallization. White solid, yield: 10% (*Method B*). R_f = 0.3 (*n*-hexane/EtOAc 7:2). Mp. 160-165 °C.

^{1}H NMR (600 MHz, D_6-DMSO): δ (ppm) = 1.28-1.32 (m, 1H, H-9), 1.35-1.40 (m, 1H, H-10), 1.60-1.70 (m, 2H, H-10, H-11), 1.70-1.84 (m, 1H, H-9), 1.88-1.97 (m, 1H, H-11), 2.01-2.05 (m, 1H, H-12), 2.19-2.28 (m, 1H, H-12), 4.75-4.80 (m, 1H, H-8), 4.88 (d, J = 4.2 Hz, 1H, OH), 5.17-5.20 (m, 2H, H-6, H-7), 5.62 (s, 1H, H-4), 7.25-7.31 (m, 2H, Ar), 7.31-7.35 (m, 1H, Ar), 7.35-7.41 (m, 2H, Ar), 7.49-7.55 (m, 2H, Ar), 7.56-7.61 (m, 1H, Ar), 7.82-7.89 (m, 2H, Ar).

^{13}C NMR (150 MHz, D_6-DMSO): δ (ppm) = 23.1, 23.5, 36.7, 42.2, 66.6, 68.4, 86.9, 127.2, 128.0, 128.3, 128.4, 128.9, 131.7, 132.8, 136.5, 136.9, 166.9.

HRMS calcd. for $C_{22}H_{24}NOS^+$ ($[M+H]^+$): 350.1573. Found: 350.1568.

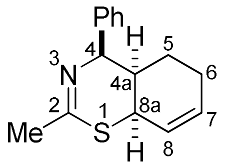

(4*S,4a*S**,8a*S**)-2-methyl-4-phenyl-4a,5,6,8a-tetrahydro-4*H*-benzo[*e*][1,3]thiazine (20aa)**

Formed from thioacetamide (**8a**), benzaldehyde (**9a**), and 1,3-cyclohexadiene (**19**) via *General methods for the synthesis of thiazines*. Purification: column chromatography (eluent: *n*-hexane/EtOAc 4:1), then a second round of column chromatography (eluent: *n*-hexane/EtOAc 10:1), then crystallization. White solid, yield: 47% (*Method B*). R_f = 0.4 (*n*-hexane/EtOAc 10:1). Mp. 87-89 °C.

^{1}H NMR (500 MHz, D_6-DMSO): δ (ppm) = 1.23-1.42 (m, 2H, H-5), 1.85-1.97 (m, 1H, H-6), 2.03-2.17 (m, 2H, H-4a and H-6), 2.23-2.28 (m, 3H, CH_3), 4.27-4.32 (m, 1H, H-8a), 4.47-4.51 (m, 1H, H-4), 5.64-5.70 (m, 1H, H-8), 5.77-5.83 (m, 1H, H-7), 7.23-7.29 (m, 1H, Ar), 7.34-7.40 (m, 2H, Ar), 7.40-7.45 (m, 2H, Ar).

^{13}C NMR (125 MHz, D_6-DMSO): δ (ppm) =15.5, 25.6, 27.9, 35.2, 41.5, 65.0, 125.8, 126.6, 127.5, 128.2, 130.0, 143.0, 159.1.

HRMS calcd. for $C_{15}H_{18}NS^+$ ($[M+H]^+$): 244.1154. Found: 244.1153.

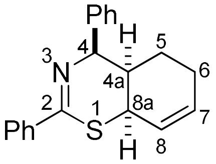

(4*S,4a*S**,8a*S**)-2,4-diphenyl-4a,5,6,8a-tetrahydro-4*H*-benzo[*e*][1,3]thiazine (20ba)**

Formed from thiobenzamide (**8b**), benzaldehyde (**9a**), and 1,3-cyclohexadiene (**19**) via *General methods for the synthesis of thiazines*. Purification: column chromatography (eluent: *n*-hexane/EtOAc 20:1), and finally crystallization. White solid, yield: 22% (*Method B*). R_f = 0.3 (*n*-hexane/EtOAc 20:1). Mp. 98-99 °C.

^{1}H NMR (500 MHz, D_6-DMSO): δ (ppm) = 1.05-1.29 (m, 2H, H-5), 1.81-1.93 (m, 1H, H-6), 1.96-2.08 (m, 1H, H-6), 2.24-2.32 (m, 1H, H-4a), 4.55-4.63 (m, 1H, H-8a), 4.77 (d, J = 2.58 Hz, 1H, H-4), 5.76-5.83 (m, 2H, H-7 and H-8), 7.25-7.31 (m, 1H, Ar), 7.38-7.43 (m, 2H, Ar), 7.43-7.53 (m, 3H, Ar), 7.55-7.60 (m, 2H, Ar), 7.84-7.89 (m, 2H, Ar).

^{13}C NMR (125 MHz, D_6-DMSO): δ (ppm) = 16.0, 25.5, 35.2, 41.4, 64.8, 126.5, 126.6, 126.9, 127.9, 128.5, 129.0, 130.1, 131.1, 139.1, 143.6, 158.4.

HRMS calcd. for $C_{20}H_{20}NS^+$ ($[M+H]^+$): 306.1311. Found: 306.1306.

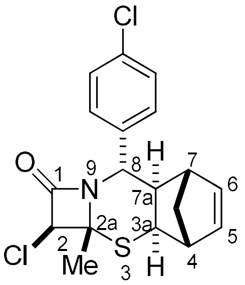

(2*S,2a*R**,3a*R**,4*R**,7*S**,7a*R**,8*S**)-2-chloro-8-(4-chlorophenyl)-2a-methyl-2,2a,3a,4,7a,8-hexahydro-4,7-methanoazeto[2,1-*b*]benzo[*e*][1,3]thiazin-1(7*H*)-one (21ab)**

Formed from thiazine **11ab** via *General method for the Staudinger ketene-imine cycloaddition of thiazines*. White solid, yield: 12%. R_f = 0.45 (*n*-hexane/EtOAc 10:1). Mp. 158-162 °C.

^{1}H NMR (500 MHz, $CDCl_3$): δ (ppm) = 1.51-1.58 (m, 1H, CH_2), 1.70 (s, 3H, CH_3), 2.19-2.24 (m, 1H, CH_2), 2.41-2.45 (m, 1H, H-7), 2.49 (dd, J = 12.22 Hz, J = 7.29 Hz, 1H, H-7a), 2.79-2.83 (m, 1H, H-4), 3.15 (d, J = 7.23 Hz, 1H, H-3a), 3.97 (d, J = 12.27 Hz, 1H, H-8), 5.02 (s, 1H, H-2), 5.99-6.04 (m, 1H, H-5), 6.14-6.20 (m, 1H, H-6), 7.37 (d, J = 8.19 Hz, 2H, Ar), 7.57 (d, J = 8.21 Hz, 2H, Ar).

^{13}C NMR (125 MHz, $CDCl_3$): δ (ppm) = 22.2, 41.8, 43.5, 44.2, 44.5, 47.6, 61.0, 67.0, 68.1, 128.9, 130.6, 133.9, 134.4, 134.7, 139.3, 165.2.

HRMS calcd. for $C_{18}H_{18}Cl_2NOS^+$ ($[M+H]^+$): 366.0481. Found: 366.0478.

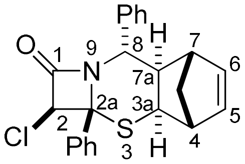

(2*R,2a*S**,3a*R**,4*R**,7*S**,7a*R**,8*S**)-2-chloro-2a,8-diphenyl-2,2a,3a,4,7a,8-hexahydro-4,7-methanoazeto[2,1-*b*]benzo[*e*][1,3]thiazin-1(7*H*)-one (21ba)**

Formed diastereoselectively from thiazine **11ba** via *General method for the Staudinger ketene-imine cycloaddition of thiazines*. White solid, yield: 58%. R_f = 0.4 (*n*-hexane/EtOAc 10:1). Mp. 206-208 °C.

^{1}H NMR (400 MHz, $CDCl_3$): δ (ppm) = 1.46-1.49 (m, 1H, CH_2), 2.14-2.24 (m, 1H, CH_2), 2.41-2.46 (m, 1H, H-7a), 2.66-2.74 (m, 1H, H-7), 2.81-2.87 (m, 1H, H-4), 3.30 (d, J = 7.0 Hz, 1H, H-3a), 4.09-4.16 (m, 1H, H-8), 5.27 (s, 1H, H-2), 6.00-6.06 (m, 1H, H-5), 6.16-6.20 (m, 1H, H-6), 7.28-7.33 (m, 2H, Ar), 7.34-7.50 (m, 6H, Ar), 7.70-7.77 (m, 2H, Ar).

^{13}C NMR (125 MHz, $CDCl_3$): δ (ppm) = 43.0, 43.5, 44.1, 44.5, 47.6, 63.3, 68.5, 73.6, 125.2, 128.6, 128.7, 128.8, 128.9, 129.5, 133.7, 136.0, 137.0, 139.7, 165.5.

HRMS calcd. for $C_{23}H_{21}ClNOS^+$ ($[M+H]^+$): 394.1027. Found: 394.1025.

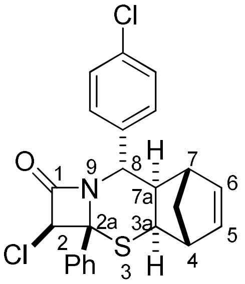

(2*S,2a*R**,3a*R**,4*R**,7*S**,7a*R**,8*S**)-2-chloro-2a-phenyl-8-(4-chlorophenyl)-2,2a,3a,4,7a,8-hexahydro-4,7-methanoazeto[2,1-*b*]benzo[*e*][1,3]thiazin-1(7*H*)-one (21bb)**

Formed diastereoselectively from thiazine **11bb** via *General method for the Staudinger ketene-imine cycloaddition of thiazines*. White solid, yield: 56%. R_f = 0.6 (*n*-hexane/EtOAc 10:1). Mp. 208-210 °C.

^{1}H NMR (400 MHz, $CDCl_3$): δ (ppm) = 1.46-1.52 (m, 1H, CH_2), 2.13-2.18 (m, 1H, CH_2), 2.39-2.43 (m, 1H, H-7a), 2.60-2.68 (m, 1H, H-7), 2.83-2-87 (m, 1H, H-4), 3.27-3.31 (m, 1H, H-3a), 4.06-4.13 (m, 1H, H-8), 5.27 (s, 1H, H-2), 6.01-6.06 (m, 1H, H-5), 6.16-6.21 (m, 1H, H-6), 7.25-7.30 (m, 2H, Ar), 7.34-7.48 (m, 5H, Ar), 7.66-7.72 (m, 2H, Ar).

^{13}C NMR (125 MHz, $CDCl_3$): δ (ppm) = 42.9, 43.5, 44.3, 44.4, 47.6, 62.6, 68.4, 73.6, 125.2, 128.8, 128.9, 129.1, 130.9, 133.9, 134.6, 134.6, 136.8, 139.6, 165.7.

HRMS calcd. for $C_{23}H_{20}Cl_2NOS^+$ ($[M+H]^+$): 428.0637. Found: 428.0634.

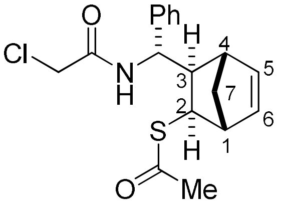

***S*-((1*R**,2*R**,3*R**,4*S**)-3-((*S**)-(2-chloroacetamido)(phenyl)methyl)bicyclo[2.2.1]hept-5-en-2-yl) ethanethioate (22aa)**

Formed from thiazine **11aa** via *General method for the Staudinger ketene-imine cycloaddition of thiazines*. White solid, yield: 46%. R_f = 0.3 (*n*-hexane/EtOAc 10:1). Mp. 215-216 °C.

^{1}H NMR (500 MHz, $CDCl_3$): δ (ppm) = 1.40-1.47 (m, 1H, H-7), 1.54-1.60 (m, 1H, H-7), 2.16 (dd, J = 11.23 Hz, J = 7.91 Hz, 1H, H-3), 2.22-2.26 (m, 1H, H-4), 2.32 (s, 3H, CH_3), 2.87-2.91 (m, 1H, H-1), 3.65 (dd, J = 7.63 Hz, J = 1.21 Hz, 1H, H-2), 3.86 (s, 2H, $ClCH_2$), 4.65 (dd, J = 11.41 Hz, J = 7.50 Hz, 1H, N-CH), 6.08-6.13 (m, 1H, H-5), 6.15-6.20 (m, 1H, H-6), 6.70 (d, J = 6.72 Hz, 1H, NH), 7.26-7.30 (m, 3H, Ar), 7.32-7.38 (m, 2H, Ar).

^{13}C NMR (125 MHz, $CDCl_3$): δ (ppm) = 30.6, 42.5, 44.9, 45.0, 45.1, 47.8, 51.1, 57.3, 127.1, 127.7, 128.7, 136.2, 139.3, 141.8, 164.6, 196.1.

HRMS calcd. for $C_{18}H_{21}ClNO_2S^+$ ($[M+H]^+$): 350.0976. Found: 350.0974.

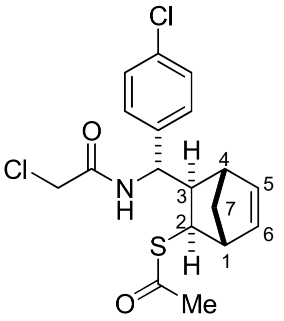

***S*-((1*R**,2*R**,3*R**,4*S**)-3-((*S**)-(2-chloroacetamido)(4-chlorophenyl)methyl)bicyclo[2.2.1]hept-5-en-2-yl) ethanethioate (22ab)**

Formed from thiazine **11ab** via *General method for the Staudinger ketene-imine cycloaddition of thiazines*. White solid, yield: 25%. R_f = 0.75 (*n*-hexane/EtOAc 1:1). Mp. 175-178 °C.

^{1}H NMR (500 MHz, $CDCl_3$): δ (ppm) = 1.45 (d, J = 9.23 Hz, 1H, H-7), 1.53 (d, J = 9.22 Hz, 1H, H-7), 2.12 (dd, J = 11.33 Hz, J = 7.86 Hz, 1H, H-3), 2.20-2.25 (m, 1H, H-4), 2.32 (s, 3H, CH_3), 2.87-2.93 (m, 1H, H-1), 3.61-3.67 (m, 1H, H-2), 3.85 (s, 2H, $ClCH_2$), 4.60 (dd, J = 11.47 Hz, J = 7.09 Hz, 1H, N-CH), 6.08-6.14 (m, 1H, H-5), 6.16-6.21 (m, 1H, H-6), 6.69 (d, J = 6.55 Hz, 1H, NH), 7.21 (d, J = 8.21 Hz, 2H, Ar), 7.32 (d, J = 8.23 Hz, 2H, Ar).

^{13}C NMR (125 MHz, $CDCl_3$): δ (ppm) = 30.6, 42.4, 44.9, 44.9, 45.1, 47.7, 51.0, 56.9, 128.5, 128.9, 133.5, 136.3, 139.2, 140.4, 164.8, 196.1.

HRMS calcd. for $C_{18}H_{20}Cl_2NO_2S^+$ ($[M+H]^+$): 384.0586. Found: 384.0585.

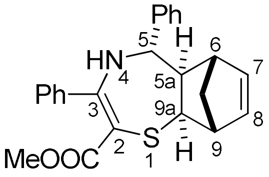

(5*S,5a*R**,6*S**,9*R**,9a*R**)-3,5-diphenyl-4,5,5a,6,9,9a-hexahydro-6,9-methanobenzo[*f*][1,4]thiazepine-2-carboxylic acid methyl ester (23ba)**

Formed from β-lactam **21ba** via *General method for the ring expansion of β-lactam condensed thiazinanes*. (eluent of column chromatography: *n*-hexane/EtOAc 10:1). Yellow solid, yield: 60%. R_f = 0.2 (*n*-hexane/EtOAc 10:1). Mp. 121-122 °C.

^{1}H NMR (500 MHz, $CDCl_3$): δ (ppm) = 1.24-1.29 (m, 1H, CH_2), 1.88-1.93 (m, 1H, CH_2), 2.13-2.17 (m, 1H, H-6), 2.20-2.27 (m, 1H, H-5a), 2.96-3.00 (m, 1H, H-9), 3.44 (s, 3H, OCH_3), 3.68-3.73 (m, 1H, H-9a), 4.16 (brs, 1H, NH), 6.04-6.10 (m, 2H, H-5 and H-7), 6.12-6.16 (m, 1H, H-8), 7.20-7.25 (m, 2H, Ar), 7.27-7.33 (m, 3H, Ar), 7.33-7.37 (m, 3H, Ar), 7.37-7.43 (m, 2H, Ar).

^{13}C NMR (125 MHz, $CDCl_3$): δ (ppm) = 42.8, 45.5, 47.7, 49.1, 50.2, 51.4, 61.8, 89.9, 127.4, 127.5, 128.2, 128.6, 128.7, 129.4, 135.4, 138.1, 140.9, 141.7, 157.1, 168.4.

HRMS calcd. for $C_{24}H_{24}NO_2S^+$ ($[M+H]^+$): 390.1522. Found: 390.1520.

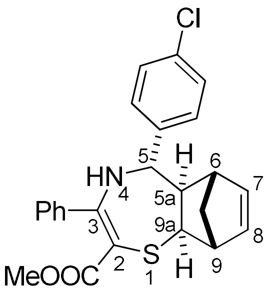

(5*S,5a*R**,6*S**,9*R**,9a*R**)-5-(4-chlorophenyl)-3-phenyl-4,5,5a,6,9,9a-hexahydro-6,9-methanobenzo[*f*][1,4]thiazepine-2-carboxylic acid methyl ester (23bb)**

Formed from β-lactam **21bb** via *General method for the ring expansion of β-lactam condensed thiazinanes* (eluent of column chromatography: *n*-hexane/EtOAc 10:1). Yellow solid, yield: 62%. R_f = 0.2 (*n*-hexane/EtOAc 10:1). Mp. 170-172 °C.

^{1}H NMR (500 MHz, $CDCl_3$): δ (ppm) = 1.25-1.30 (m, 1H, CH_2), 1.86-1.91 (m, 1H, CH_2), 2.12-2.16 (m, 1H, H-6), 2.19 (dd, J = 10.94 Hz, J = 7.16 Hz, 1H, H-5a), 2.96-3.00 (m, 1H, H-9), 3.43 (s, 3H, OCH_3), 3.70 (d, J = 6.95 Hz, 1H, H-9a), 4.02 (broad doublet, J = 3.57 Hz, 1H, NH), 6.03-6.06 (m, 1H, H-5), 6.06-6.10 (m, 1H, H-7), 6.12-6.16 (m, 1H, H-8), 7.19-7.23 (m, 2H, Ar), 7.27-7.34 (m, 5H, Ar), 7.35-7.41 (m, 2H, Ar).

^{13}C NMR (125 MHz, $CDCl_3$): δ (ppm) = 42.8, 45.4, 47.7, 49.1, 50.2, 51.4, 61.2, 90.4, 127.5, 128.3, 128.8, 128.9, 129.6, 134.4, 135.5, 138.0, 140.1, 140.7, 156.7, 168.3.

HRMS calcd. for $C_{24}H_{23}ClNO_2S^+$ ($[M+H]^+$): 424.1133. Found: 424.1129.

3. X-ray structure determinations

The centrosymmetric crystals of **11ba**, **13aa** and **21bb** were immersed in cryo-oil, mounted in a loop, and measured at a temperature of 120 K. The X-ray diffraction data were collected on a Rigaku Oxford Diffraction SuperNova single source diffractometer with EOS detector (**11ba**) using Mo Kα radiation or Rikagu Oxford Diffraction Dualflex HyPix-Arc 100 diffractometer (**13aa** and **21bb**) using Cu Kα radiation. The *CrysAlisPro*[39,40] software package was used for cell refinements and data reductions. An analytical (**11ba**) or an empirical absorption correction using spherical harmonics (**13aa**, **21bb**) was applied to the intensities before the structure solutions (*CrysAlisPro*)[39,40]. The structures were solved by the intrinsic phasing (*SHELXT*)[41] method. Structural refinements were carried out using *SHELXL*[42] software with *SHELXLE*[43] graphical user interface. Hydrogen atoms were positioned geometrically and constrained to ride on their parent atoms, with C-H = 0.95-1.00 Å and U_{iso} = 1.2-1.5· U_{eq}(parent atom). The crystallographic details are summarized in Table S1.

Table S1. Crystal Data.

	**11ba**	**13aa**	**21bb**
CCDC	2491441	2491442	2491443
empirical formula	$C_{21}H_{19}NS$	$C_{18}H_{17}NS$	$C_{23}H_{19}Cl_2NOS$
fw	317.43	279.38	428.35
temp (K)	120(2) K	120(2) K	120(2) K
λ(Å)	0.71073 Å	1.54184 Å	1.54184 Å
crystal system	Orthorhombic	Monoclinic	Monoclinic
space group	Pbca	$P2_1/n$	$P2_1/c$
a (Å)	11.7009(2)	8.25120(7)	13.43125(18)
b (Å)	15.6961(3)	16.50920(13)	6.82967(9)
c (Å)	17.6878(4)	21.02664(18)	21.6001(3)
β (deg)	90	94.1989(7)	98.5980(12)
V (Å^3)	3248.53(10)	2856.58(4)	1959.13(4)
Z	8	8	4
ρ_{calc} (Mg/m^3)	1.298	1.299	1.452
μ(Cu Kα) (mm^{-1})	0.198	1.897	4.085
No. reflns.	51338	66327	65700
Unique reflns.	4711	6178	4252
Completeness to θ=67.684°		100.0%	100.0%
Completeness to θ=26.000°	99.8		
GOOF (F^2)	1.042	1.086	1.045
R_{int}	0.0590	0.0362	0.0775
R1^a ($I \geq 2\sigma$)	0.0415	0.0327	0.0471
wR2^b ($I \geq 2\sigma$)	0.1051	0.0876	0.1310

^{a} $R1 = \Sigma||F_o| - |F_c||/\Sigma|F_o|$. $^b wR2 = \{\Sigma[w(F_o^2 - F_c^2)^2]/ \Sigma[w(F_o^2)^2]\}^{1/2}$

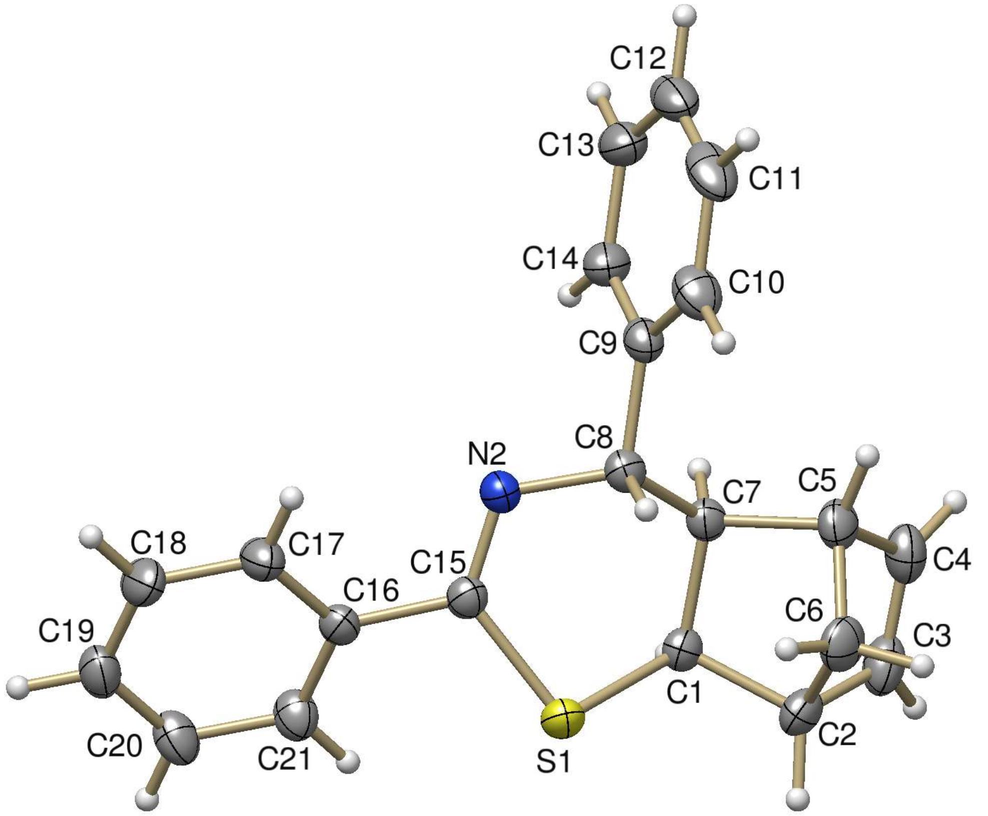

Figure S1. Thermal ellipsoid (50 % probability) plot of **11ba**.

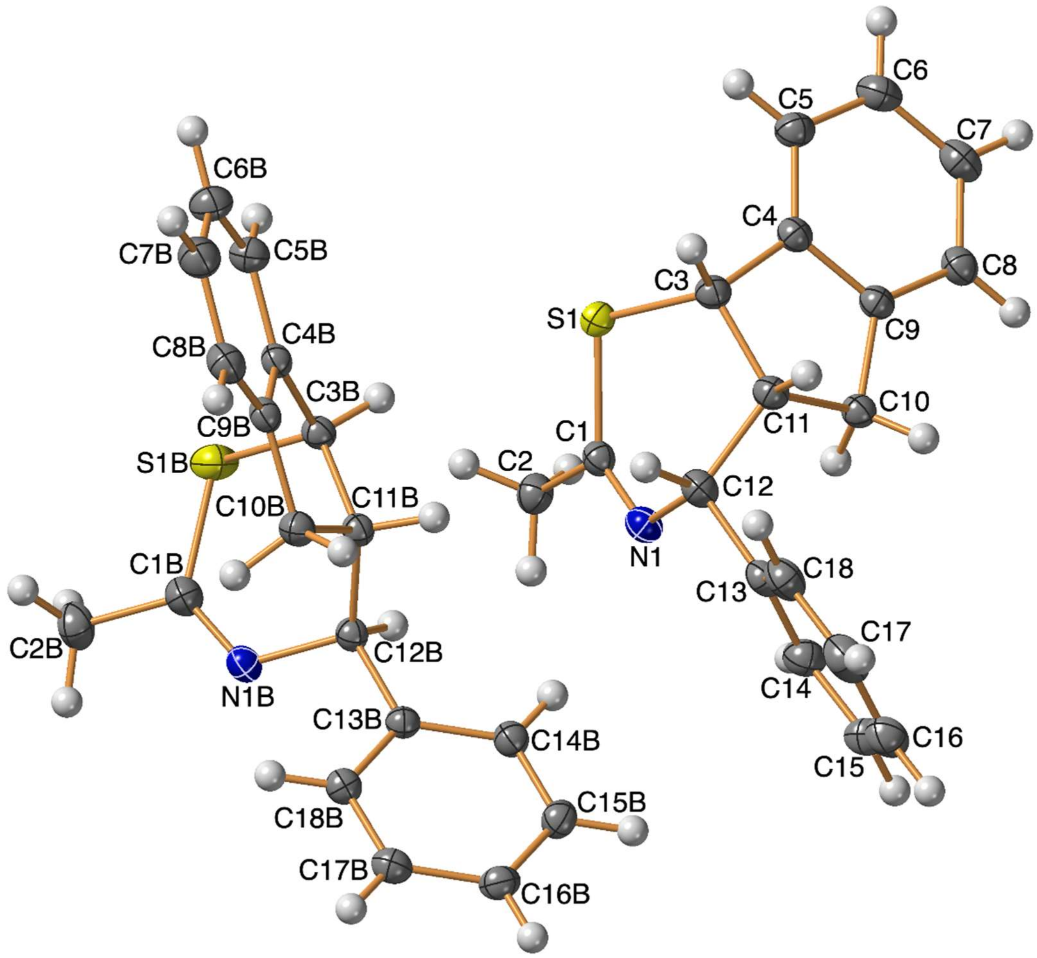

Figure S2. Thermal ellipsoid (50 % probability) plot of **13aa**.

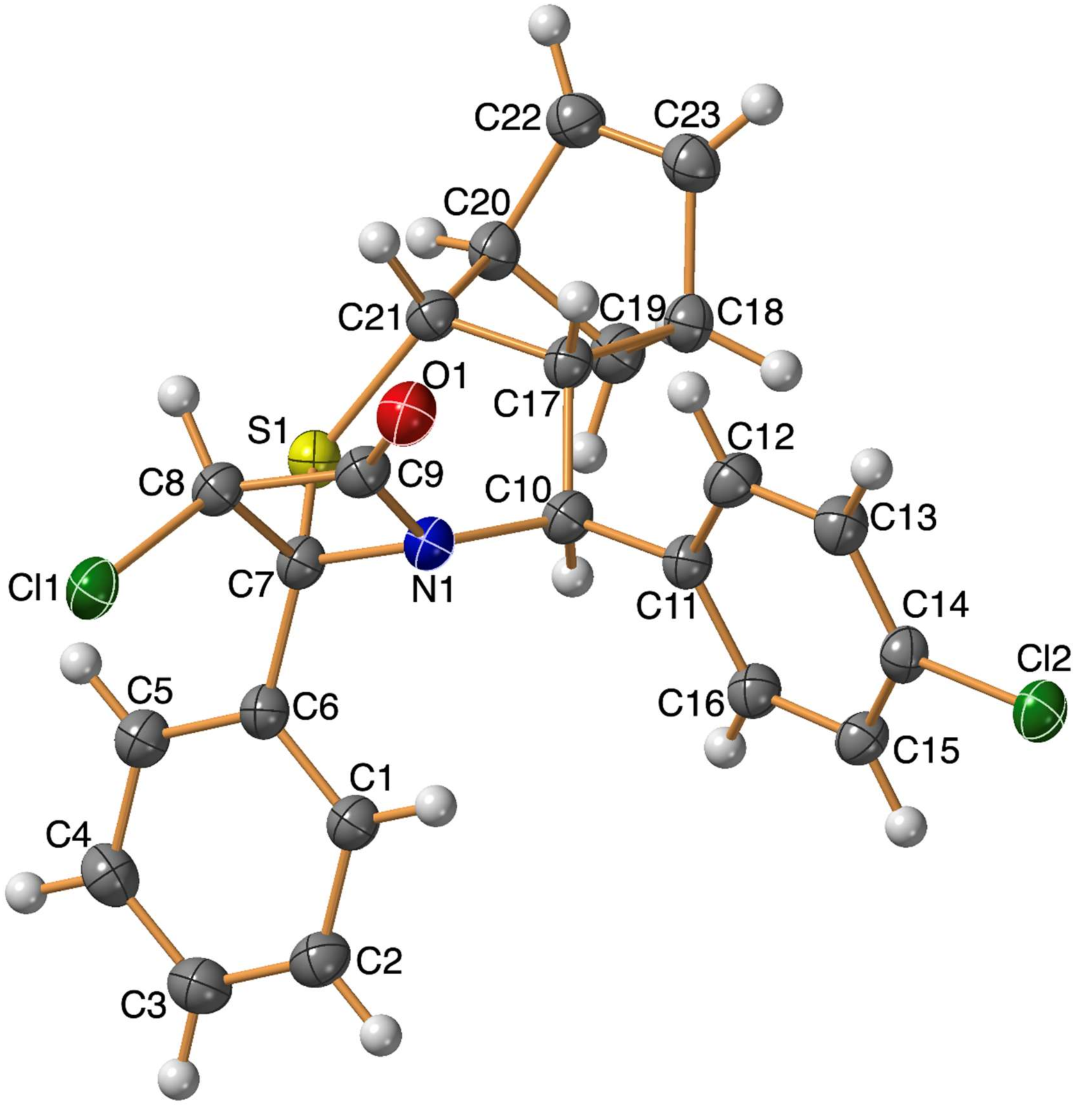

Figure S3. Thermal ellipsoid (50 % probability) plot of **21bb**.

4. Copies of NMR spectra

(4*S,4a*R**,5*S**,8*R**,8a*R**)-2-methyl-4-phenyl-4a,5,8,8a-tetrahydro-4*H*-5,8-methanobenzo[*e*][1,3]thiazine (11aa)**

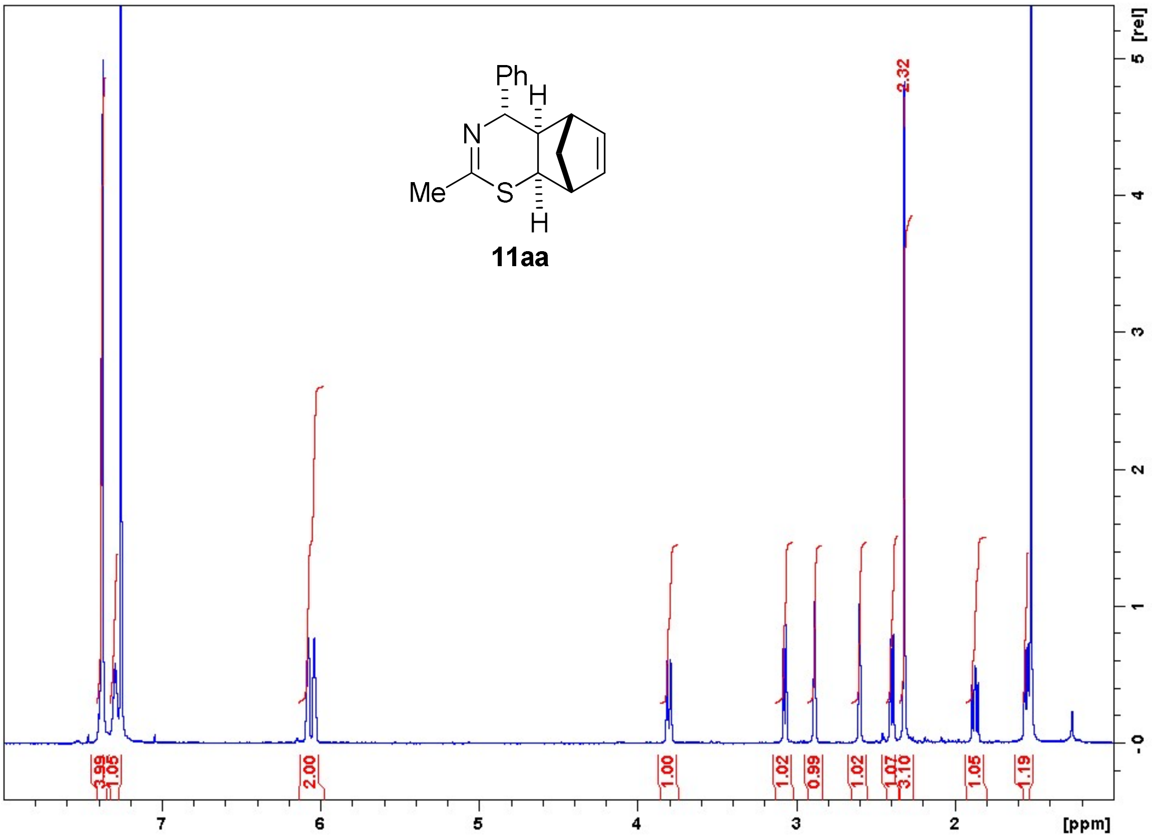

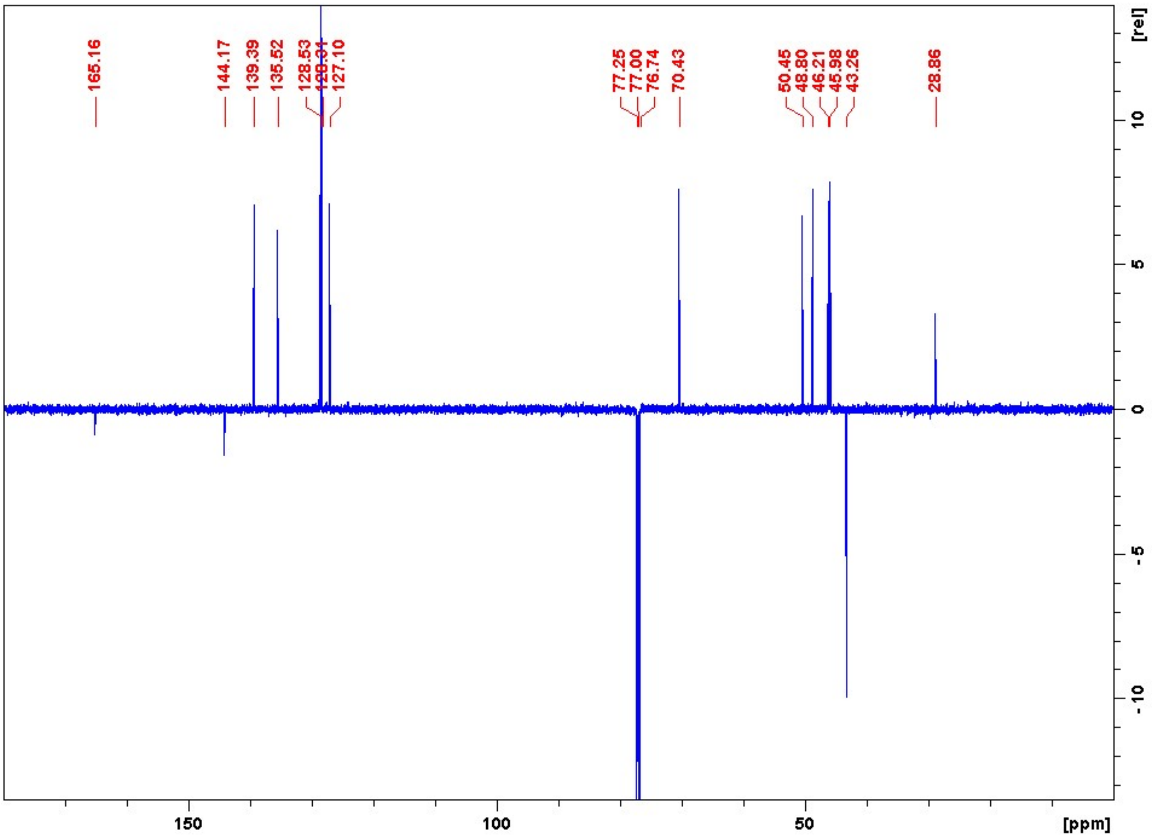

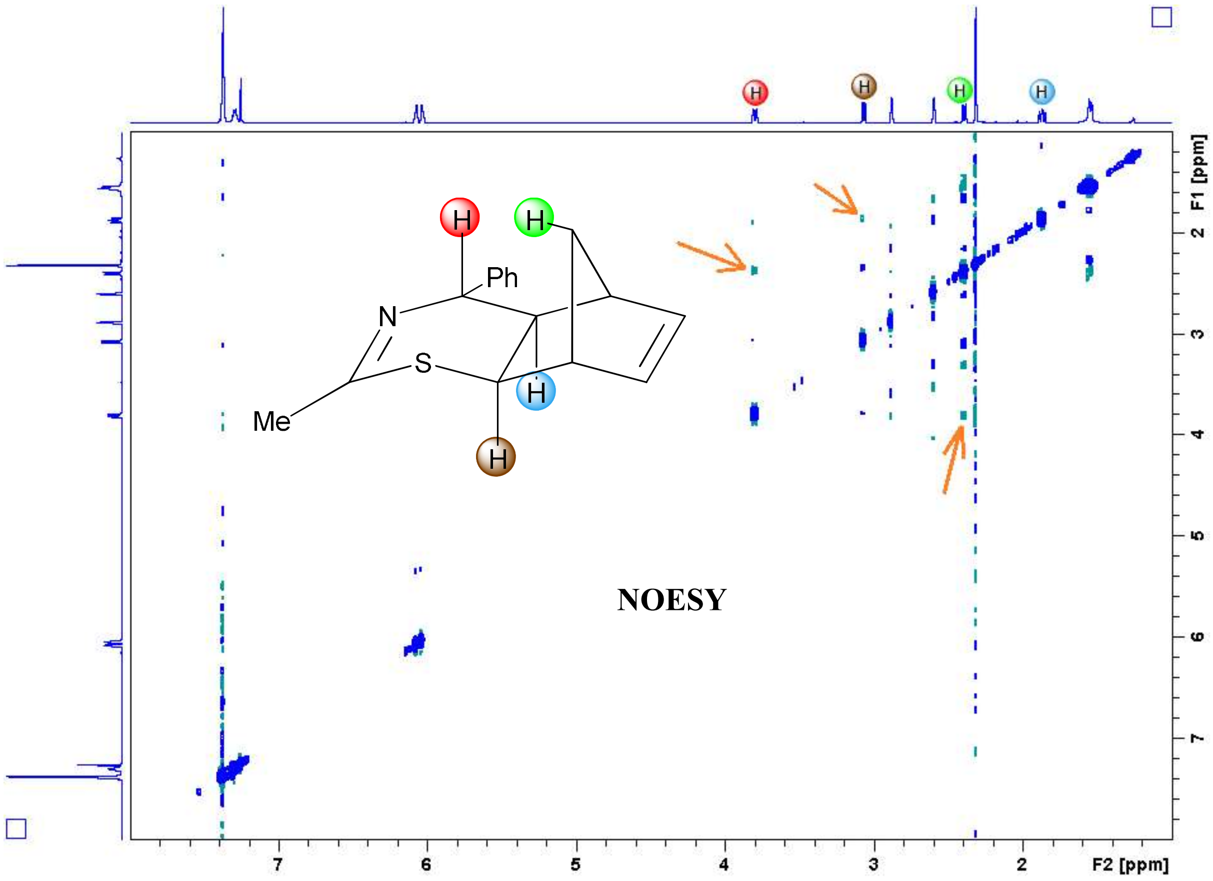

H
Ph
N
S
Me
NOESY
F1 [ppm]
F2 [ppm]

(4*S,4a*R**,5*S**,8*R**,8a*R**)-2-methyl-4-(4-chlorophenyl)-4a,5,8,8a-tetrahydro-4*H*-5,8-methanobenzo[*e*][1,3]thiazine (11ab)**

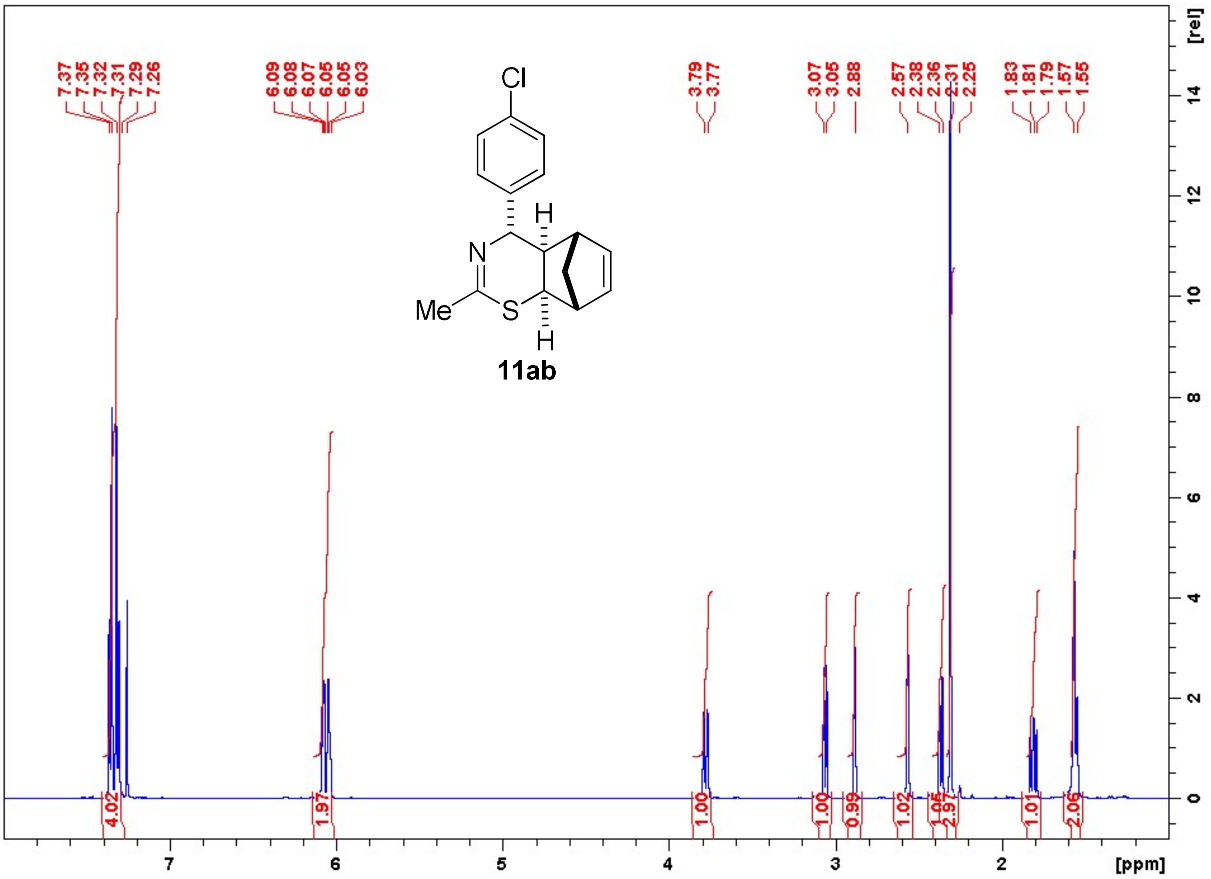

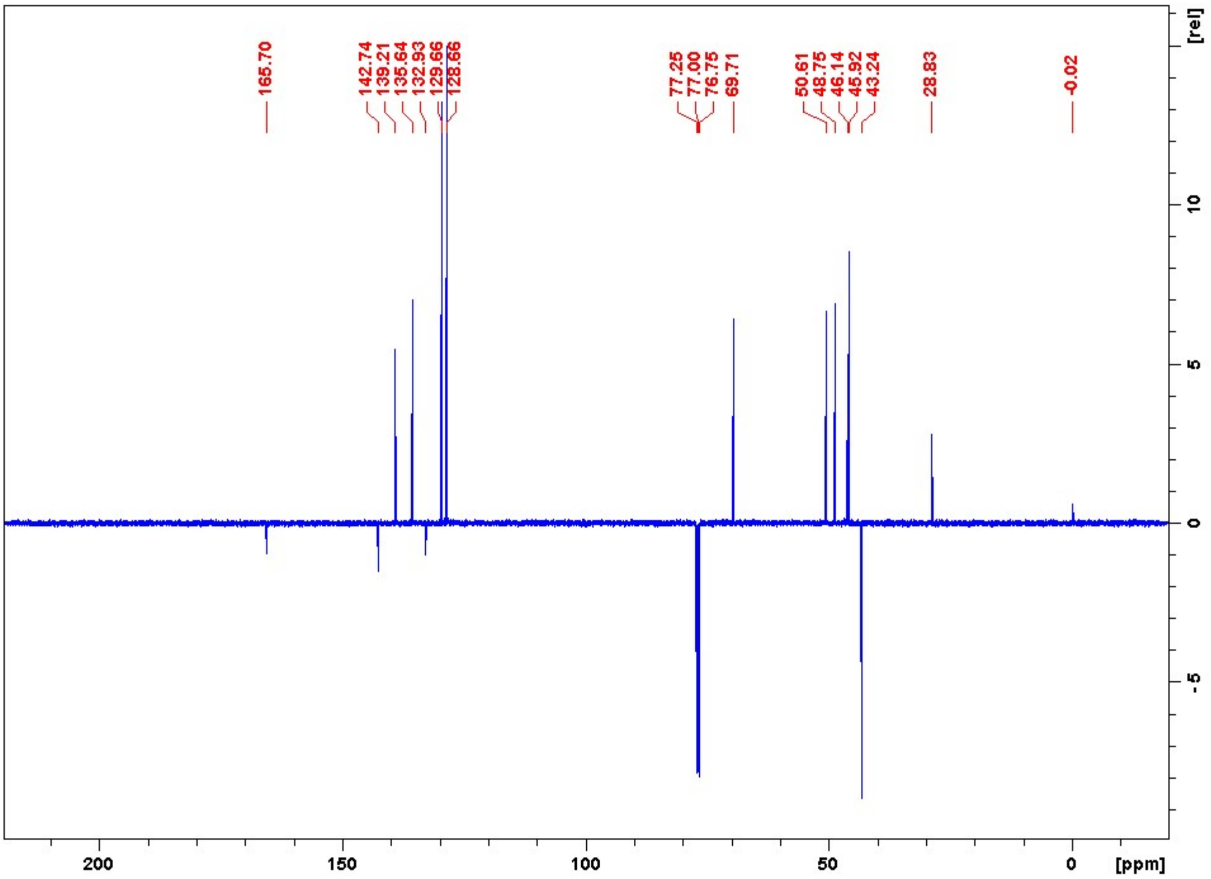

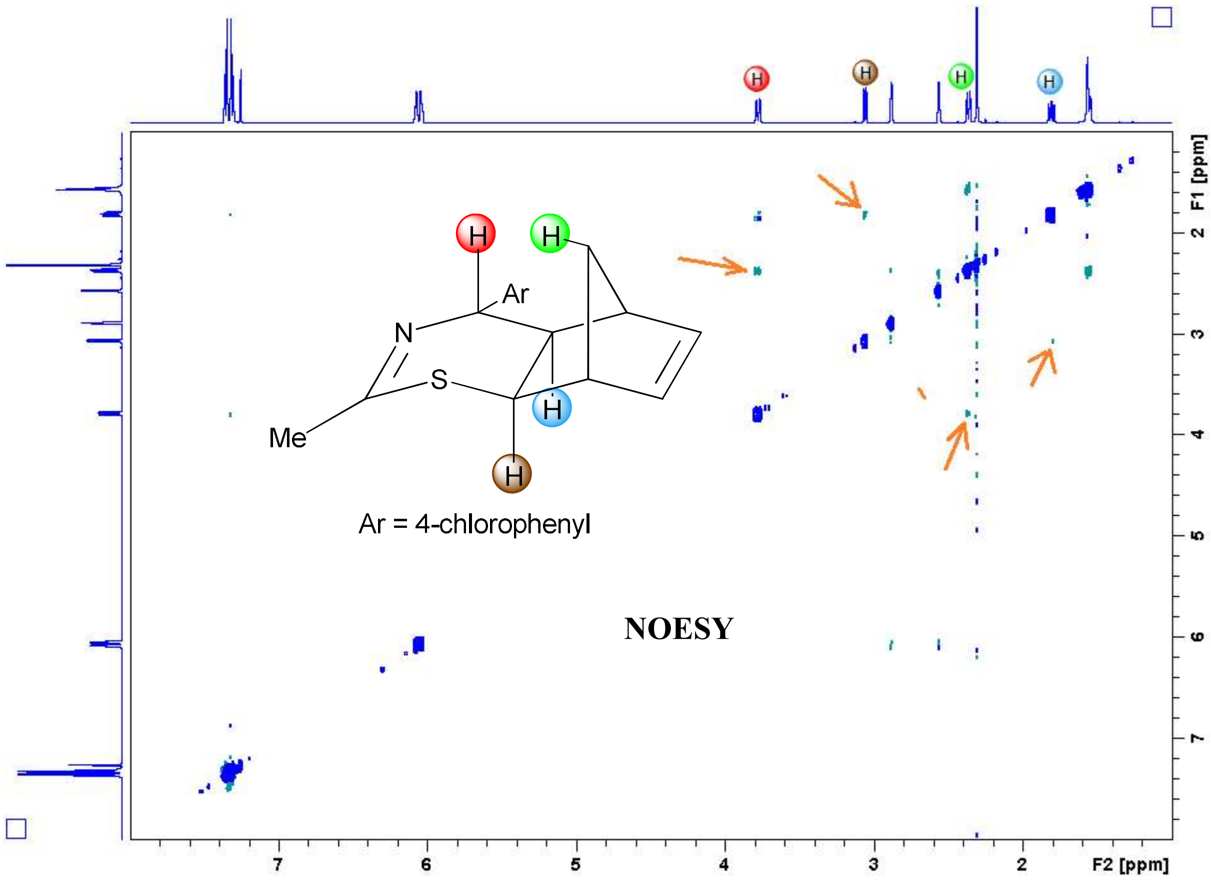

H
Ar
N
S
Me
H
H
H
Ar = 4-chlorophenyl
NOESY
F1 [ppm]
F2 [ppm]

(4*S,4a*R**,5*S**,8*R**,8a*R**)-2,4-diphenyl-4a,5,8,8a-tetrahydro-4*H*-5,8-methanobenzo[*e*][1,3]thiazine (11ba)**

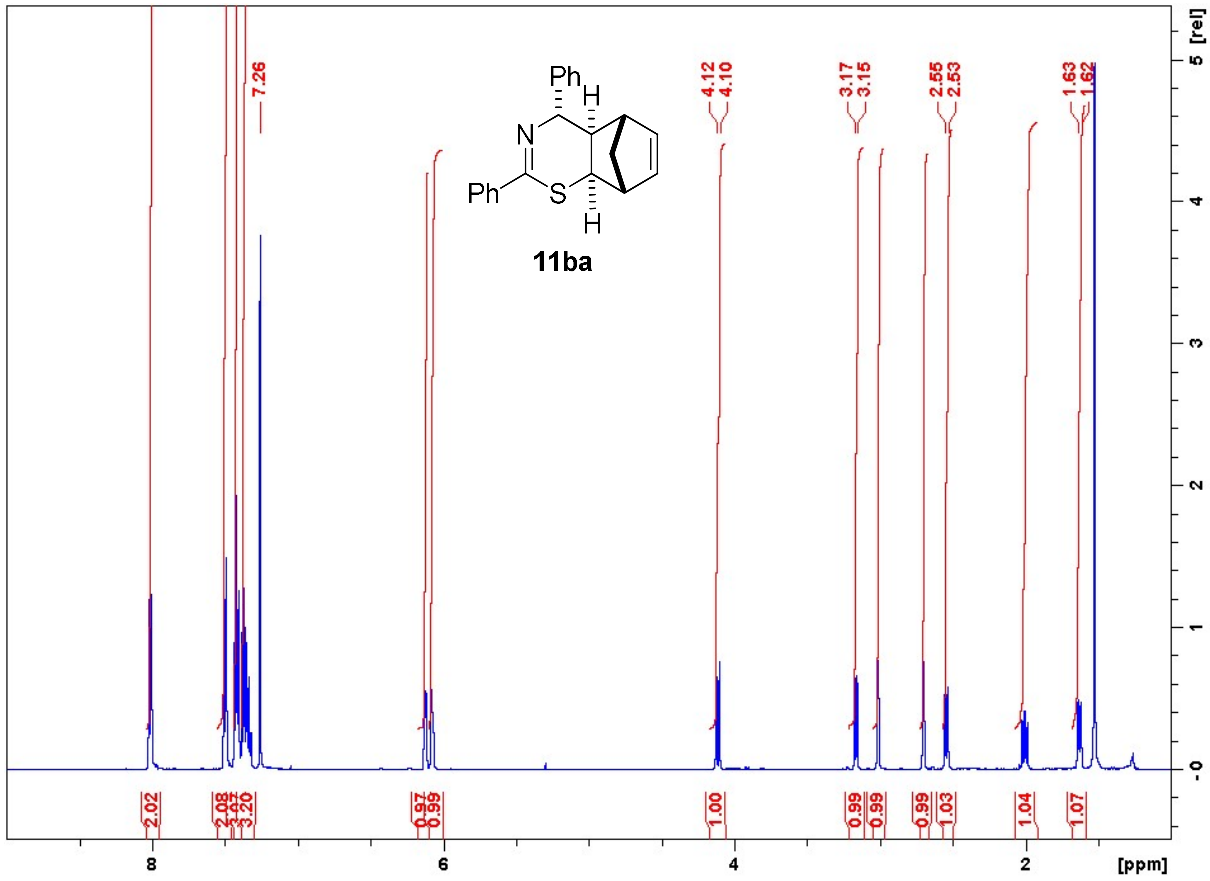

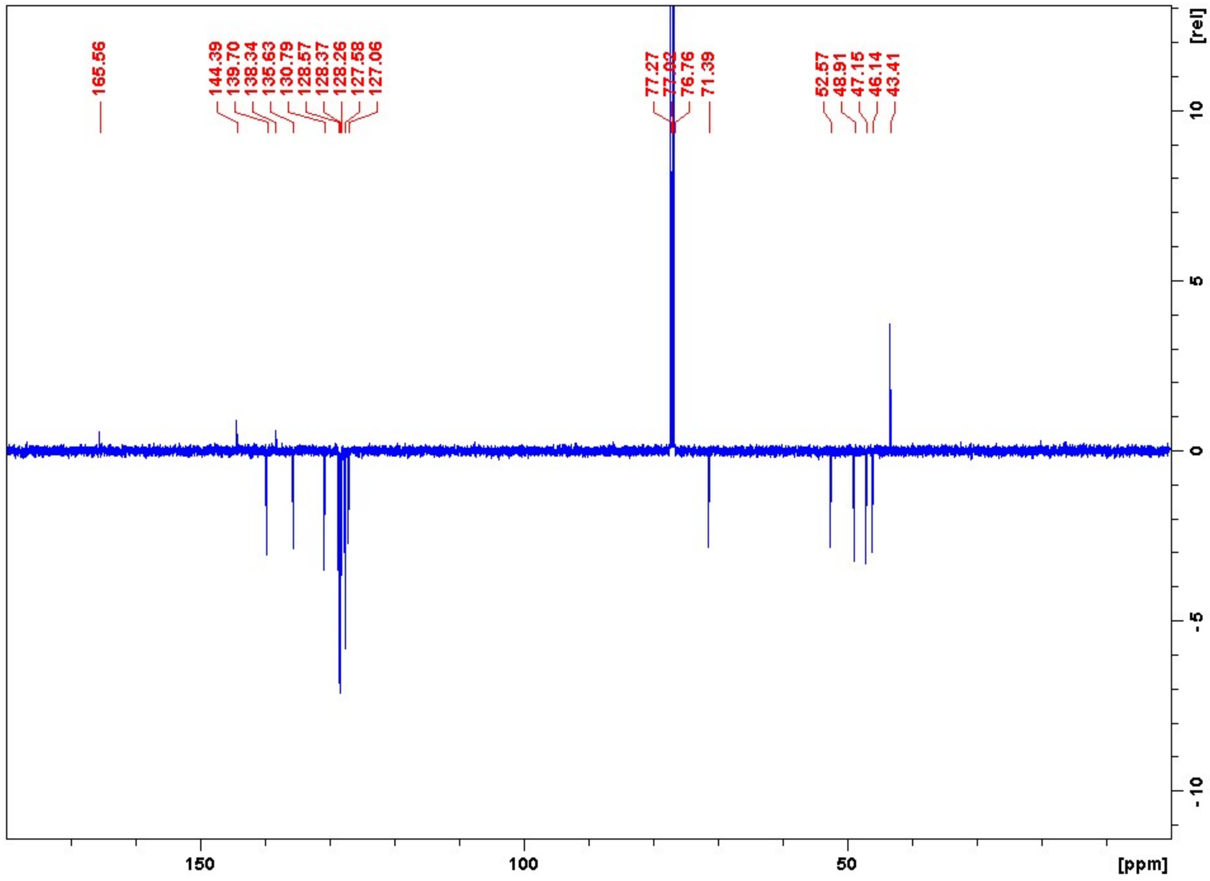

(4*S,4a*R**,5*S**,8*R**,8a*R**)-4-(4-chlorophenyl)-2-phenyl-4a,5,8,8a-tetrahydro-4*H*-5,8-methanobenzo[*e*][1,3]thiazine (11bb)**

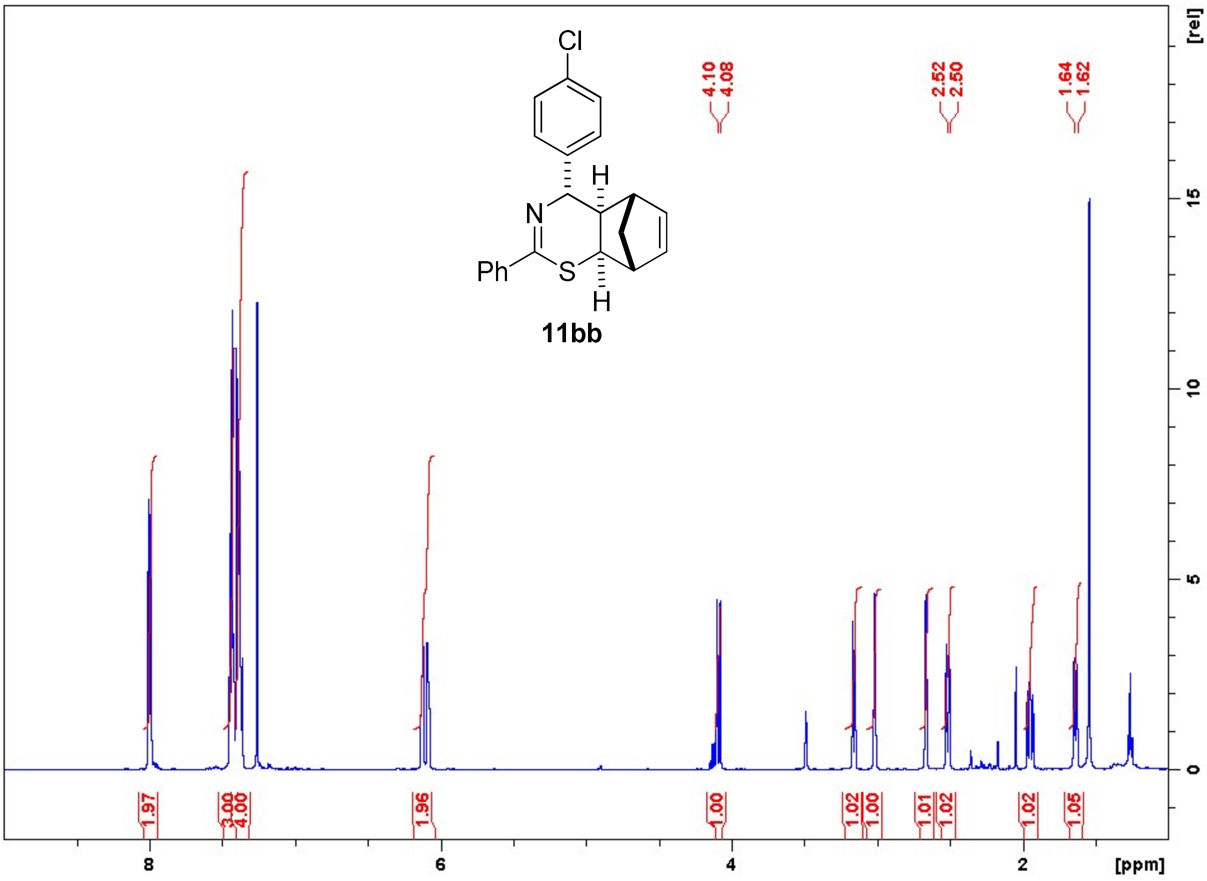

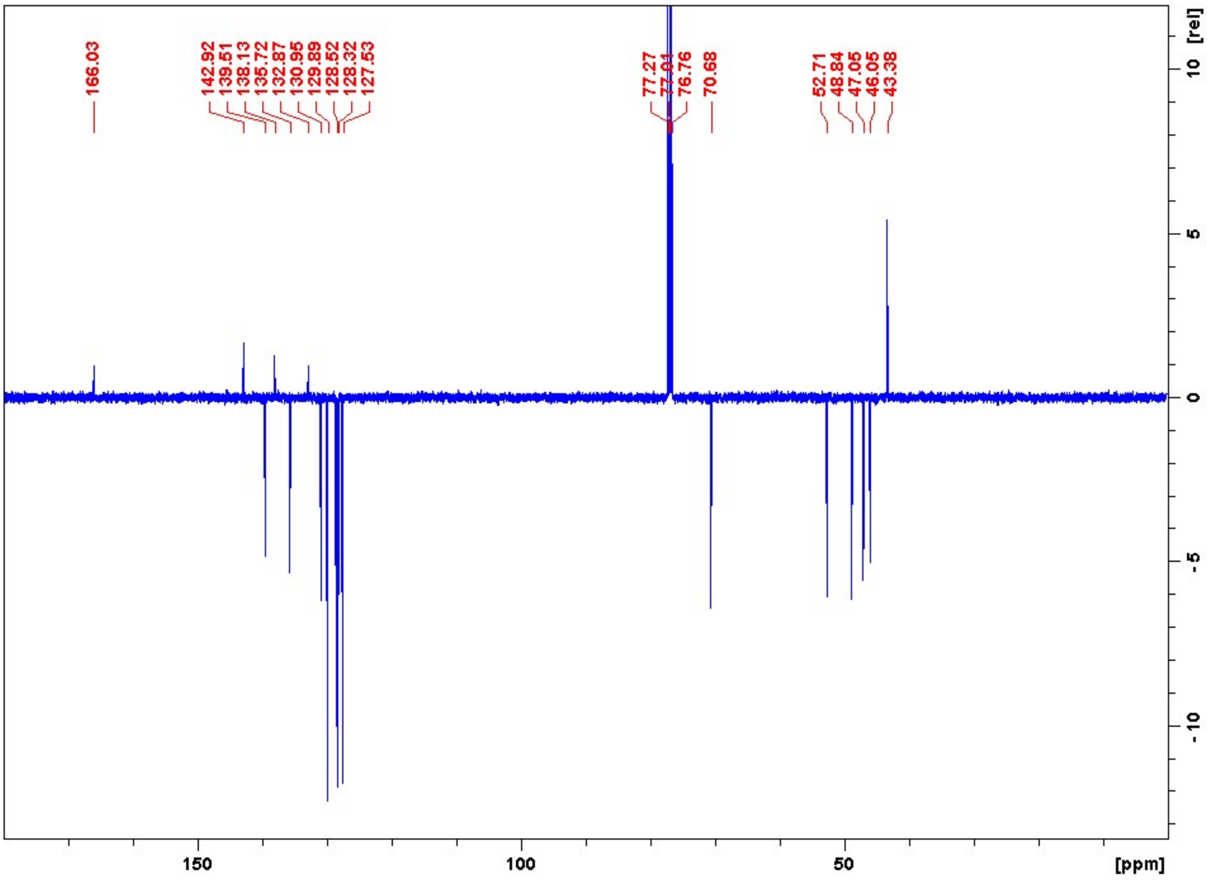

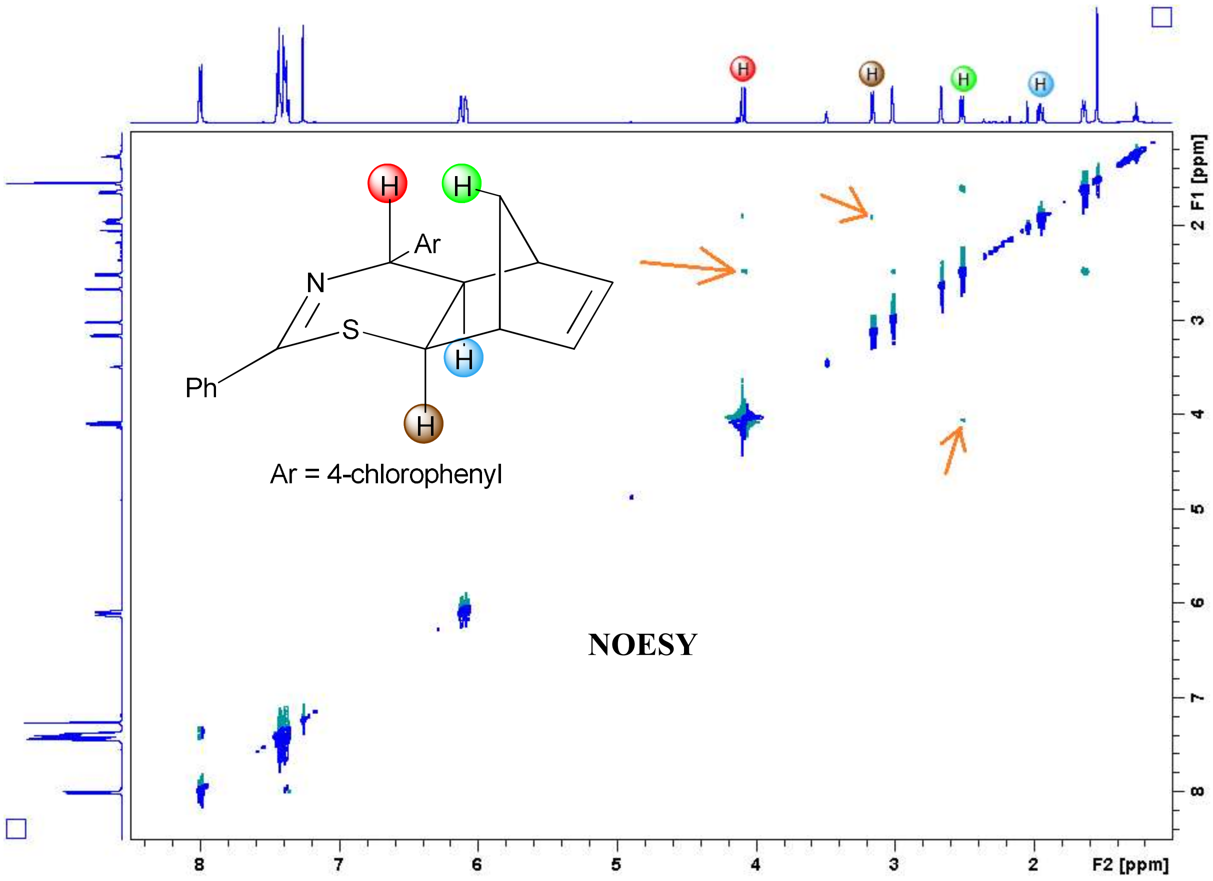
Ar
N
S
Ph
Ar = 4-chlorophenyl
NOESY
F1 [ppm]
F2 [ppm]

(4*R,4a*R**,9b*S**)-4-phenyl-2-methyl-4,4a,5,9b-tetrahydroindeno[2,1-*e*][1,3]thiazine (13aa)**

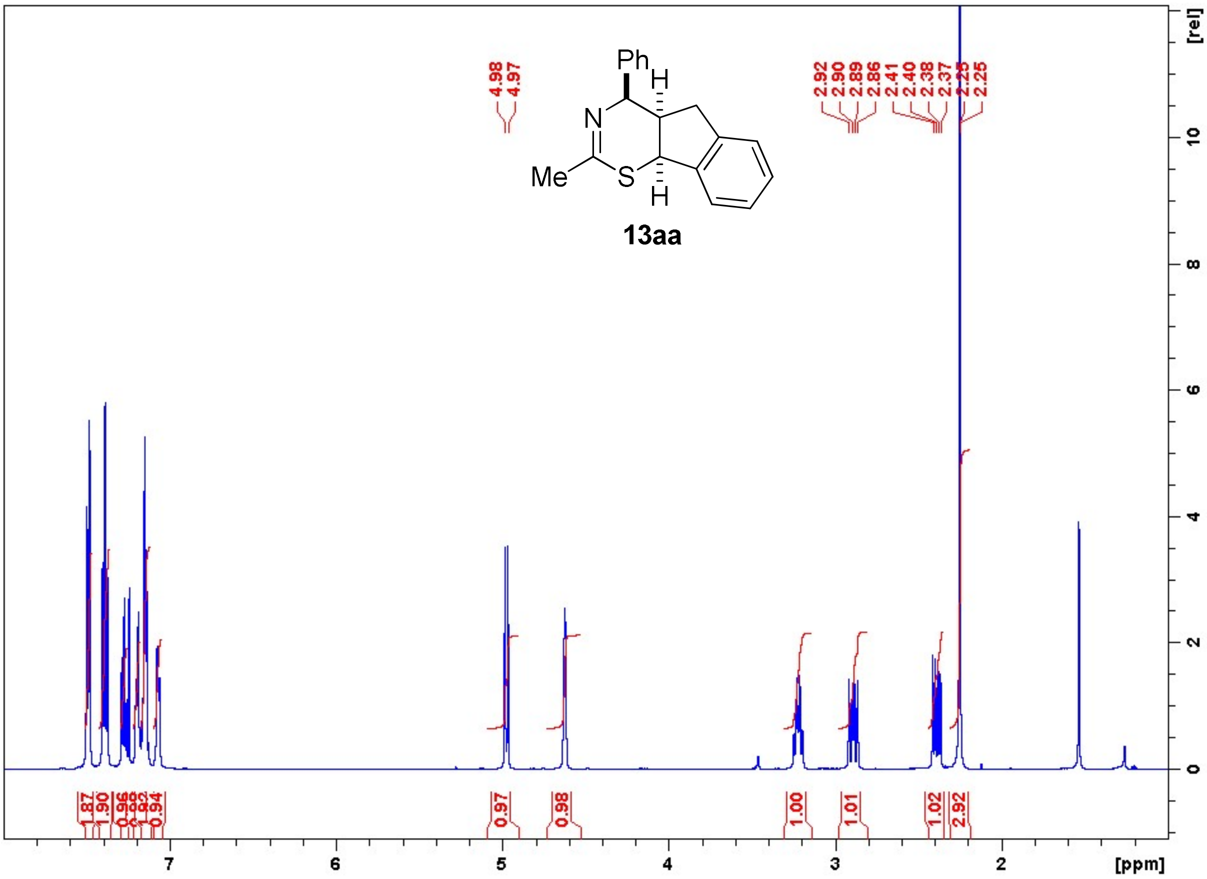

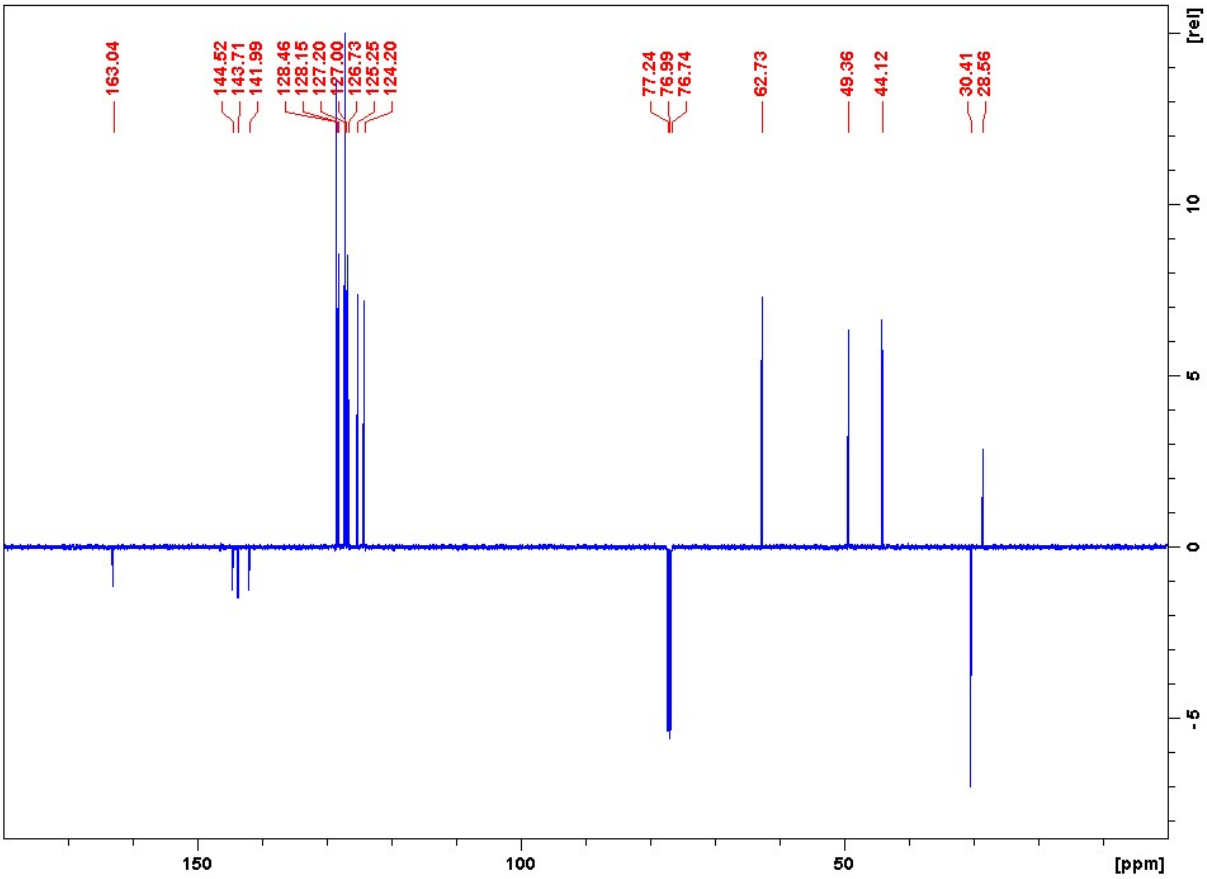

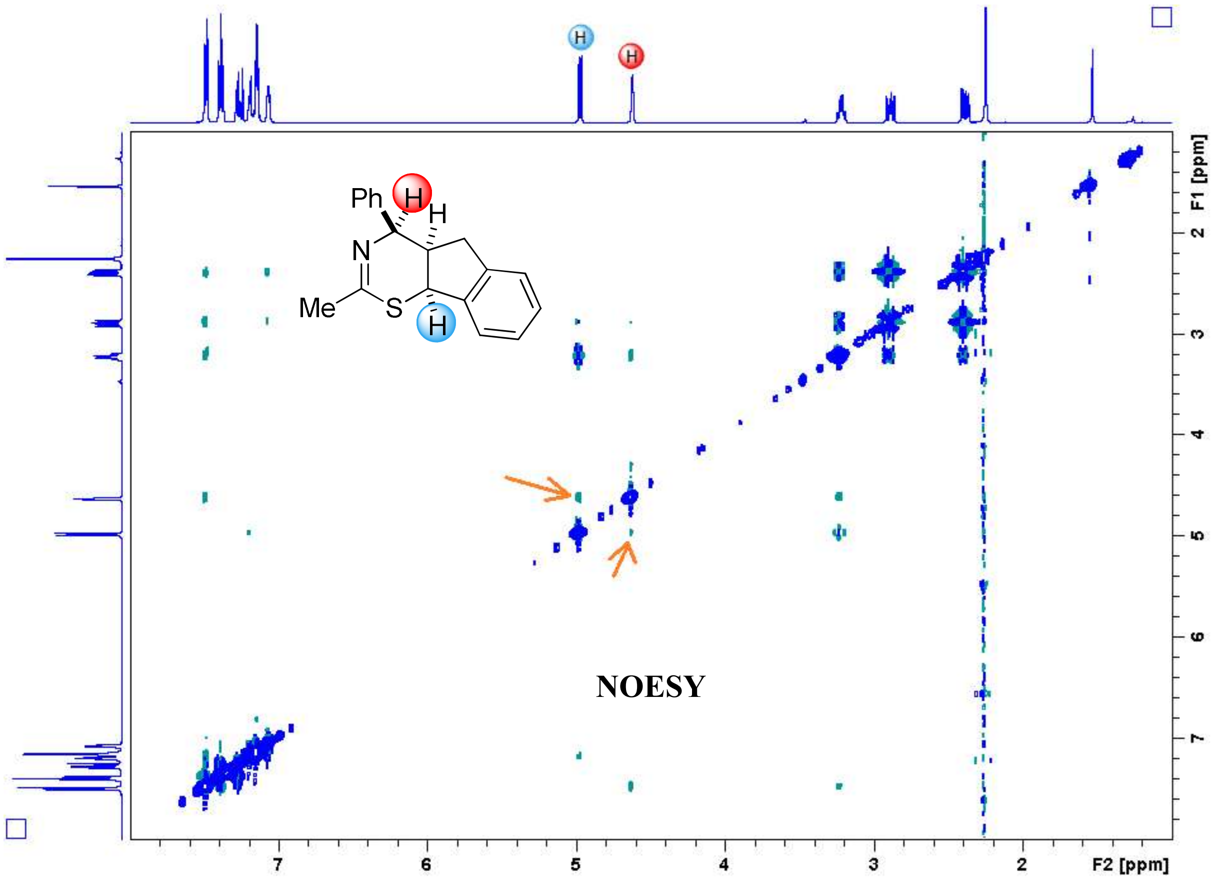
H
H
Ph
H
N
Me
S
H
F1 [ppm]
NOESY
7
6
5
4
3
2
F2 [ppm]

(4*R,4a*R**,9b*S**)-2,4-diphenyl-4,4a,5,9b-tetrahydroindeno[2,1-*e*][1,3]thiazine (13ba)**

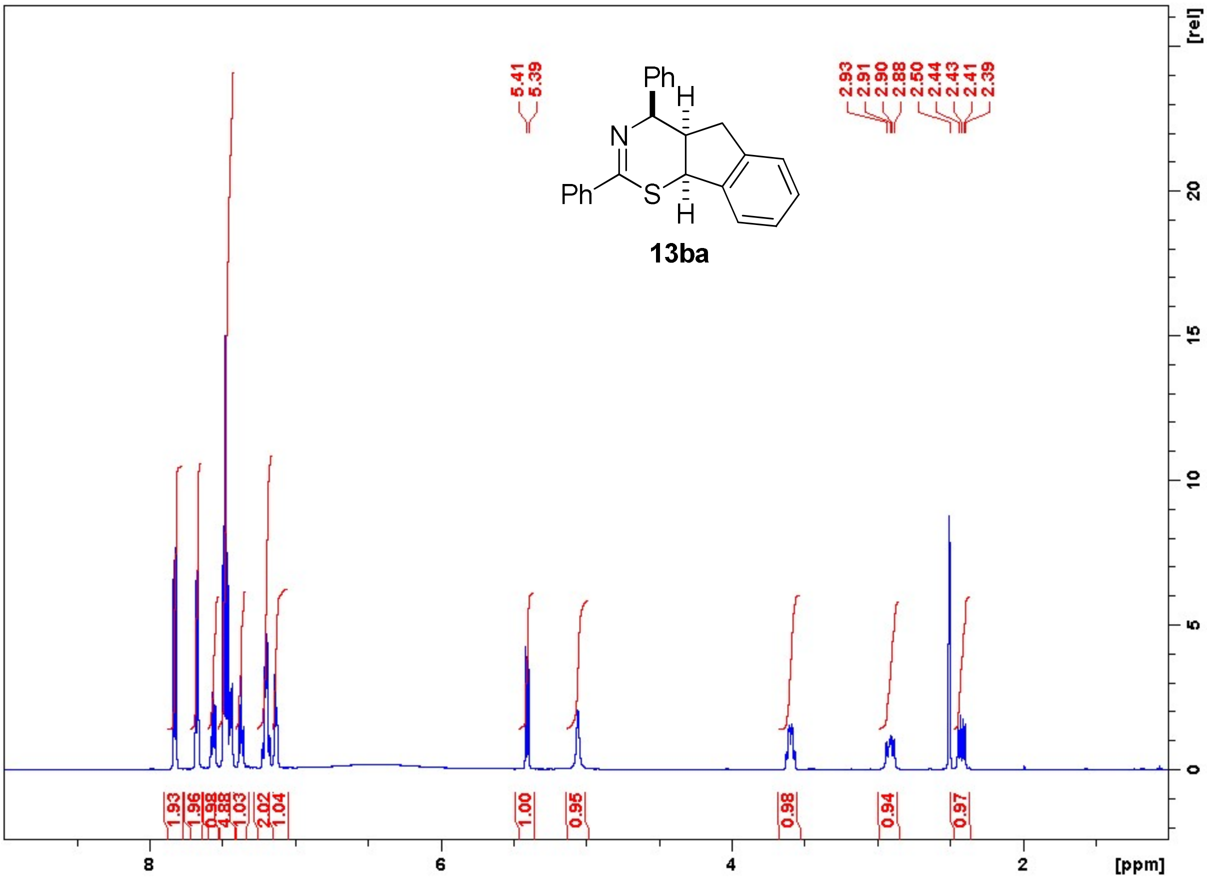

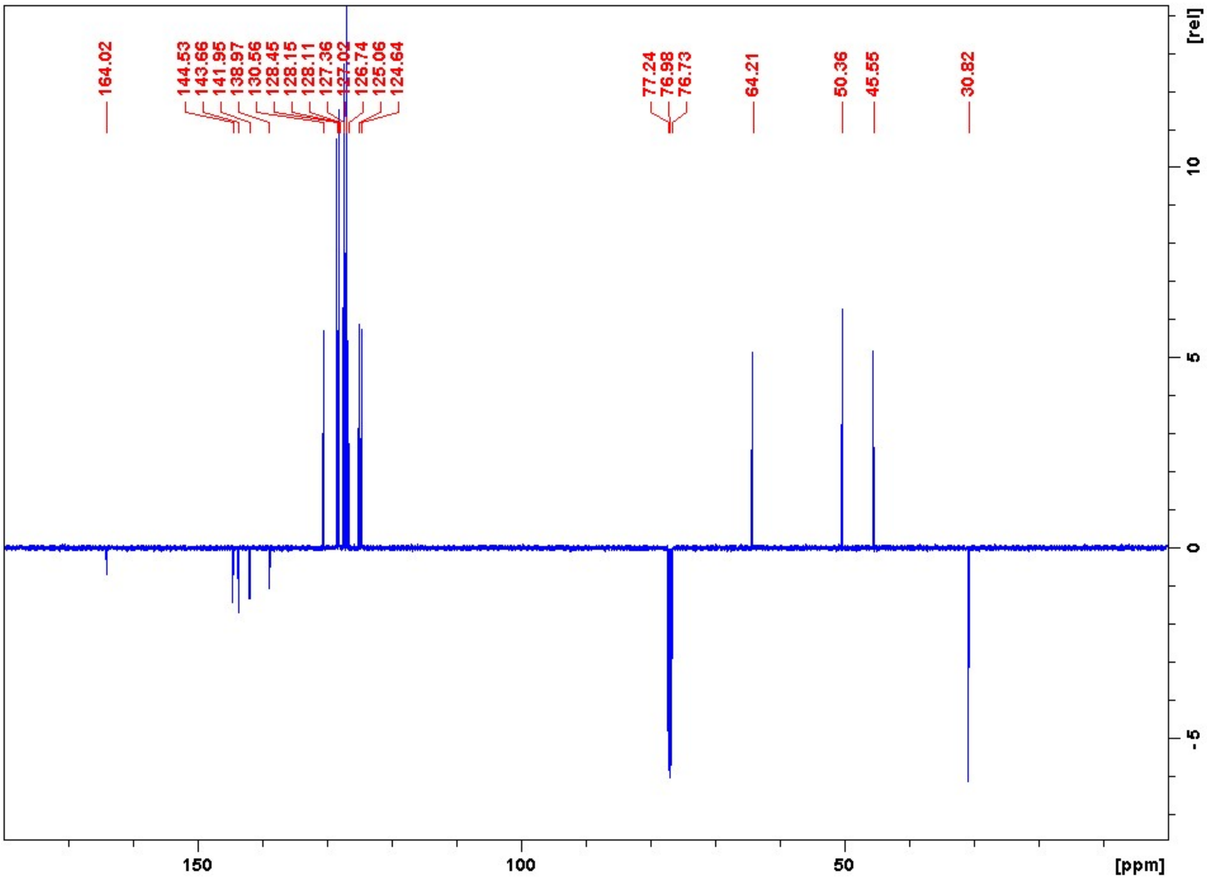

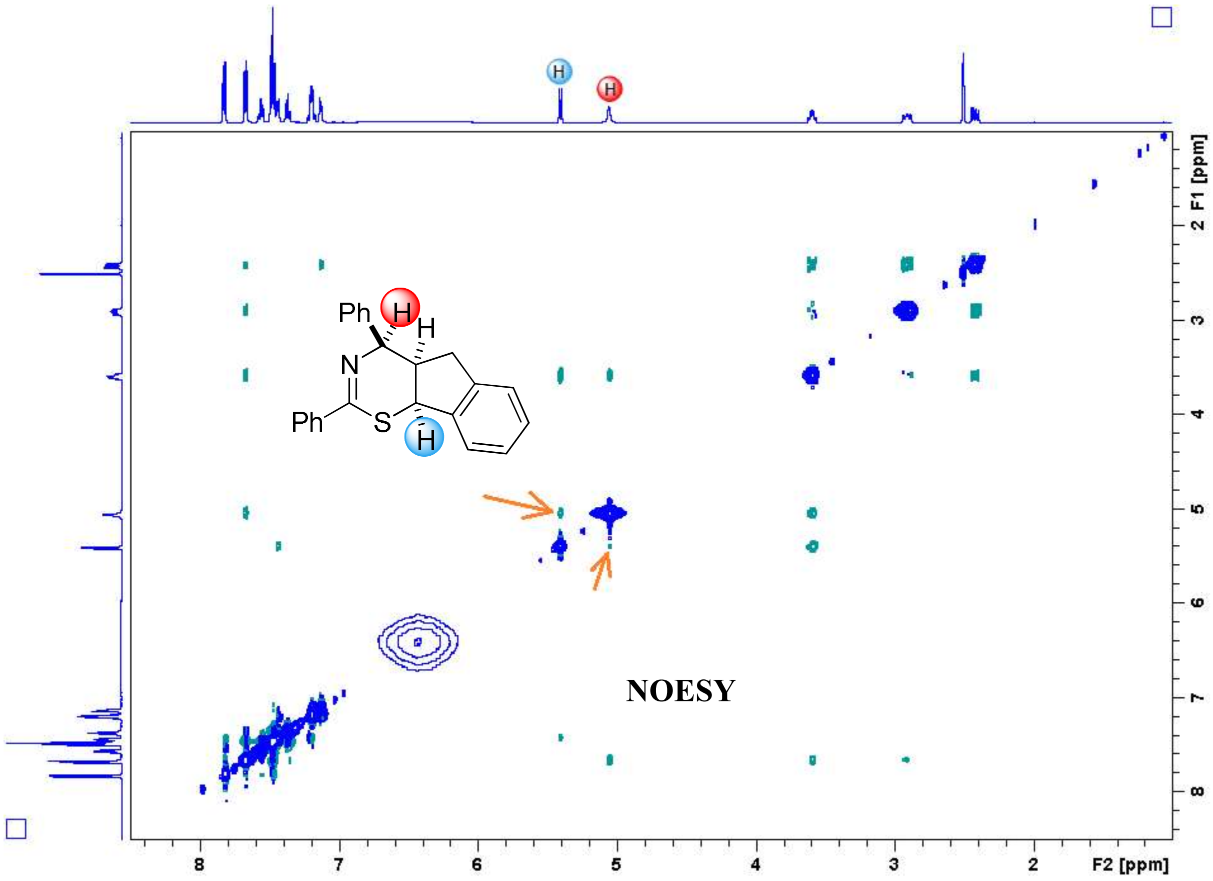
NOESY
Ph
H
H
N
Ph
S
H
F1 [ppm]
F2 [ppm]

(4*R,4a*R**,10a*R**,*Z*)-2-methyl-4-phenyl-4a,5,6,9,10,10a-hexahydro-4*H*-cycloocta[*e*][1,3]thiazine (15aa)**

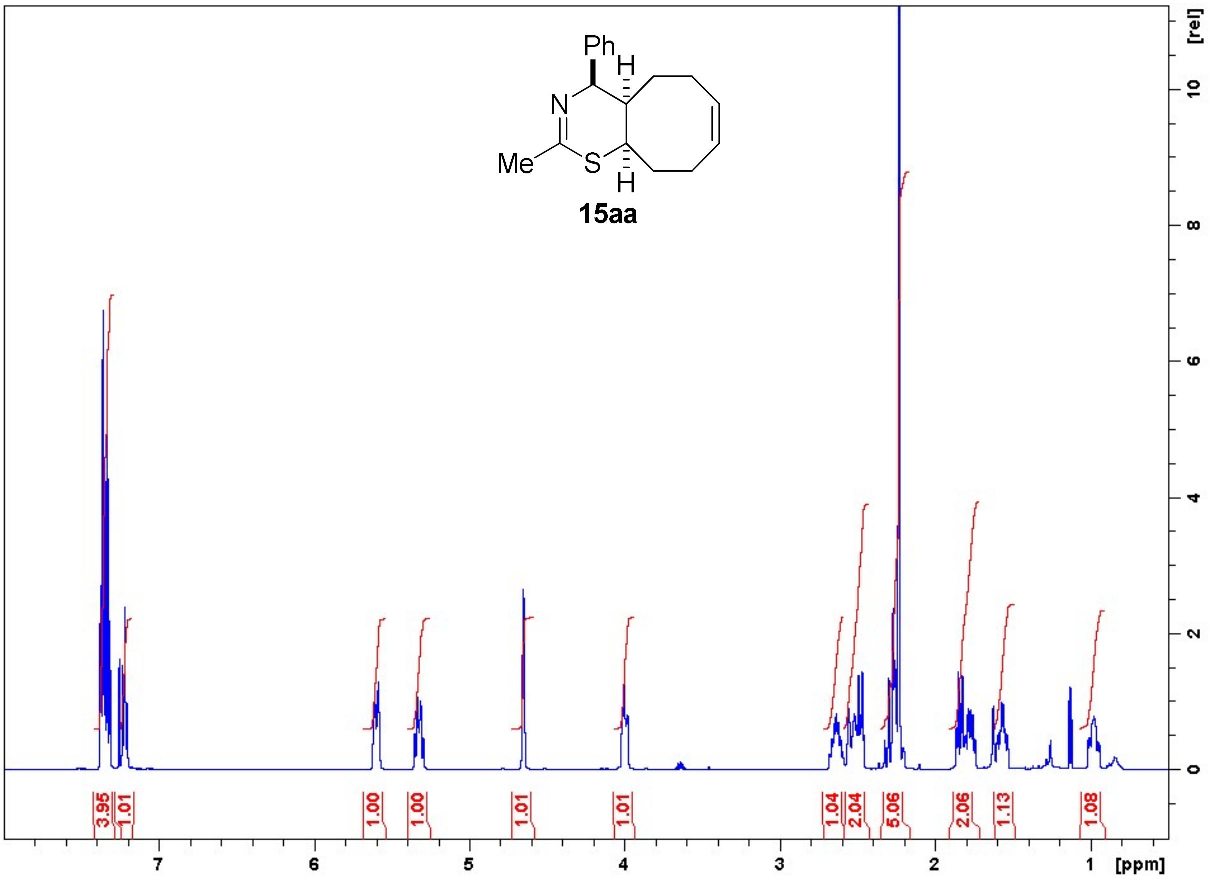

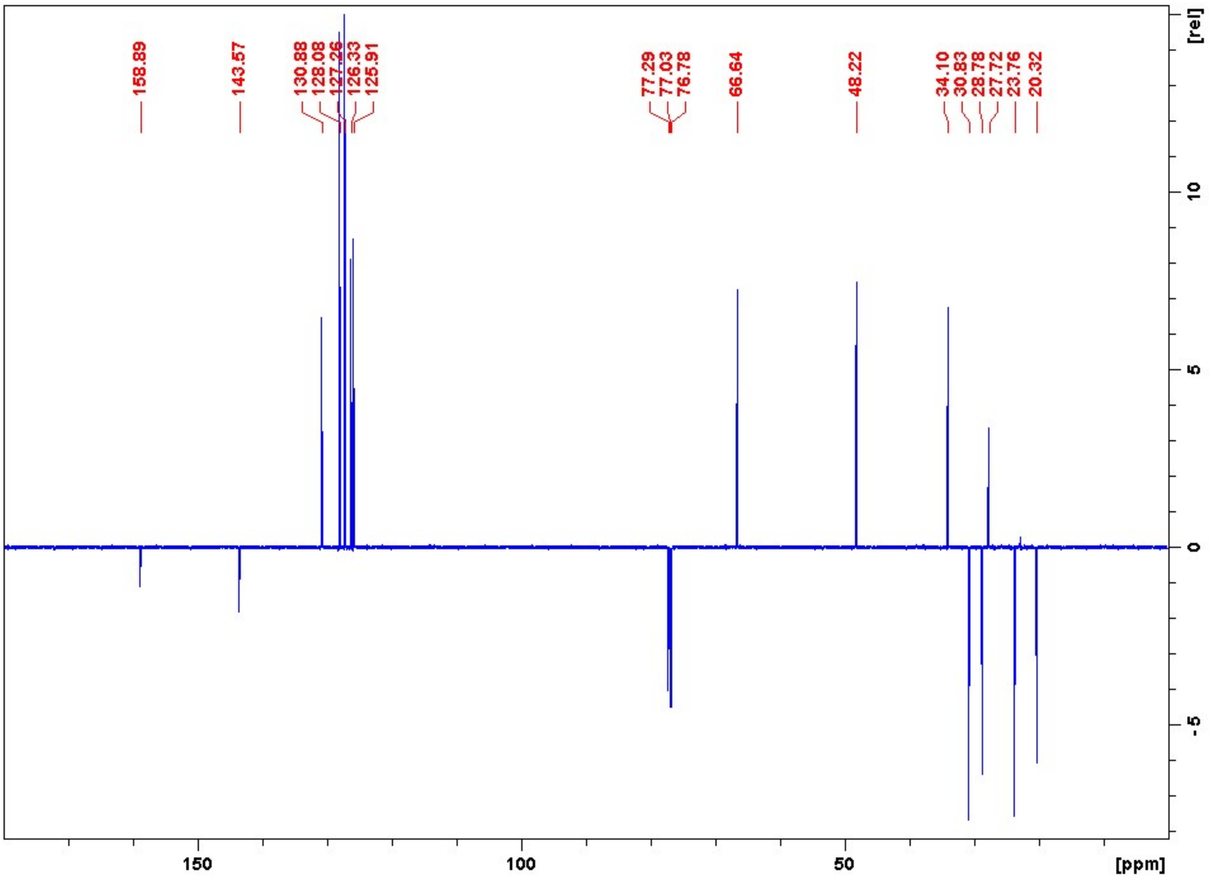

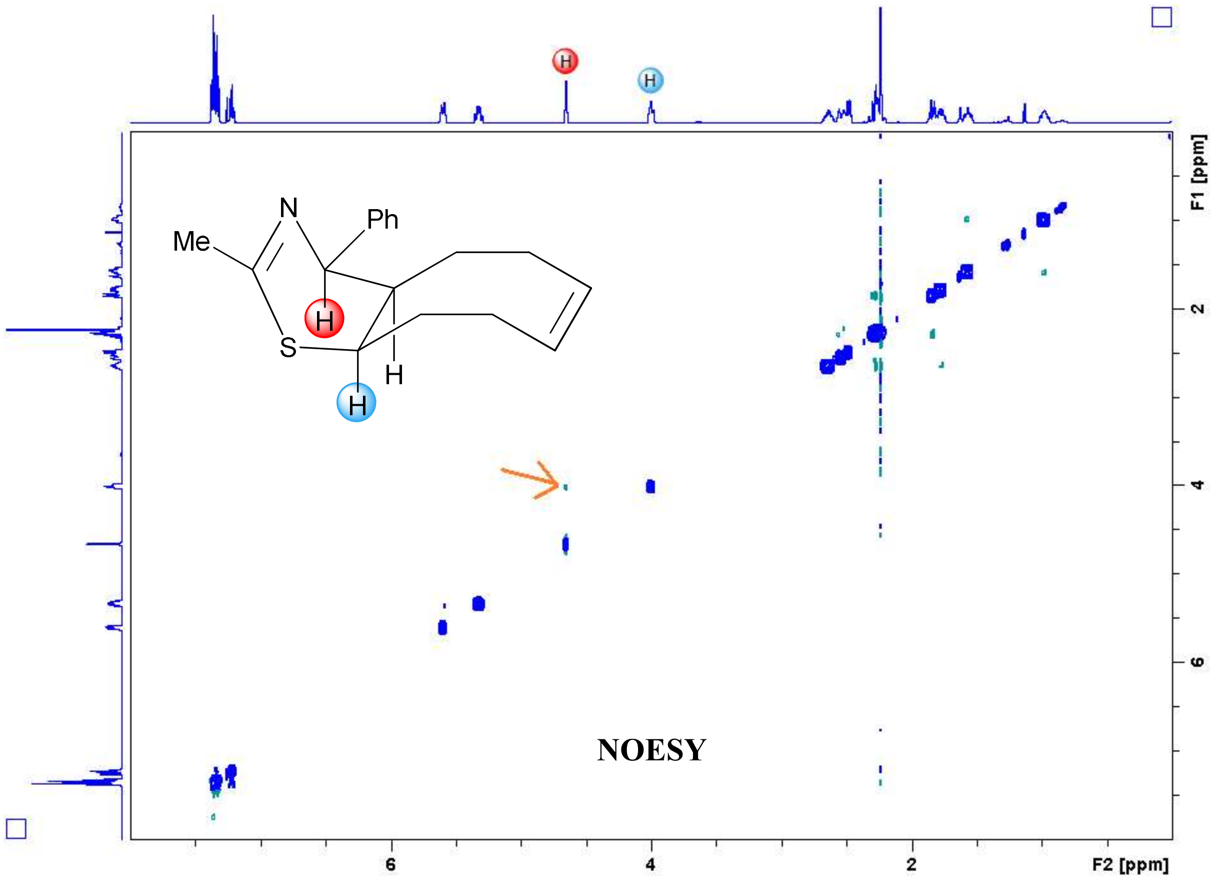
Me
N
Ph
H
S
H
H
NOESY
F1 [ppm]
F2 [ppm]
2
4
6

(4*R,4a*R**,10a*R**,*Z*)-2,4-diphenyl-4a,5,6,9,10,10a-hexahydro-4*H*-cycloocta[*e*][1,3]thiazine (15ba)**

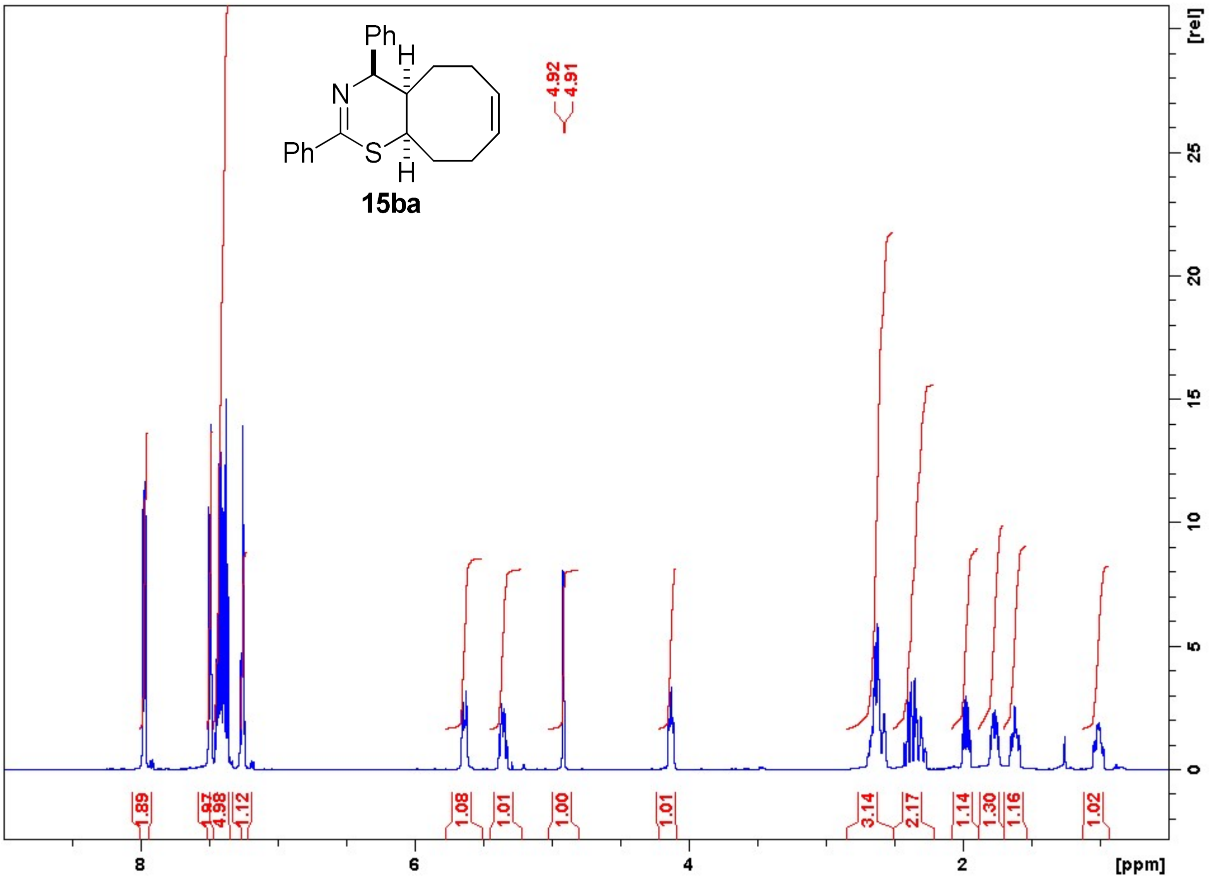

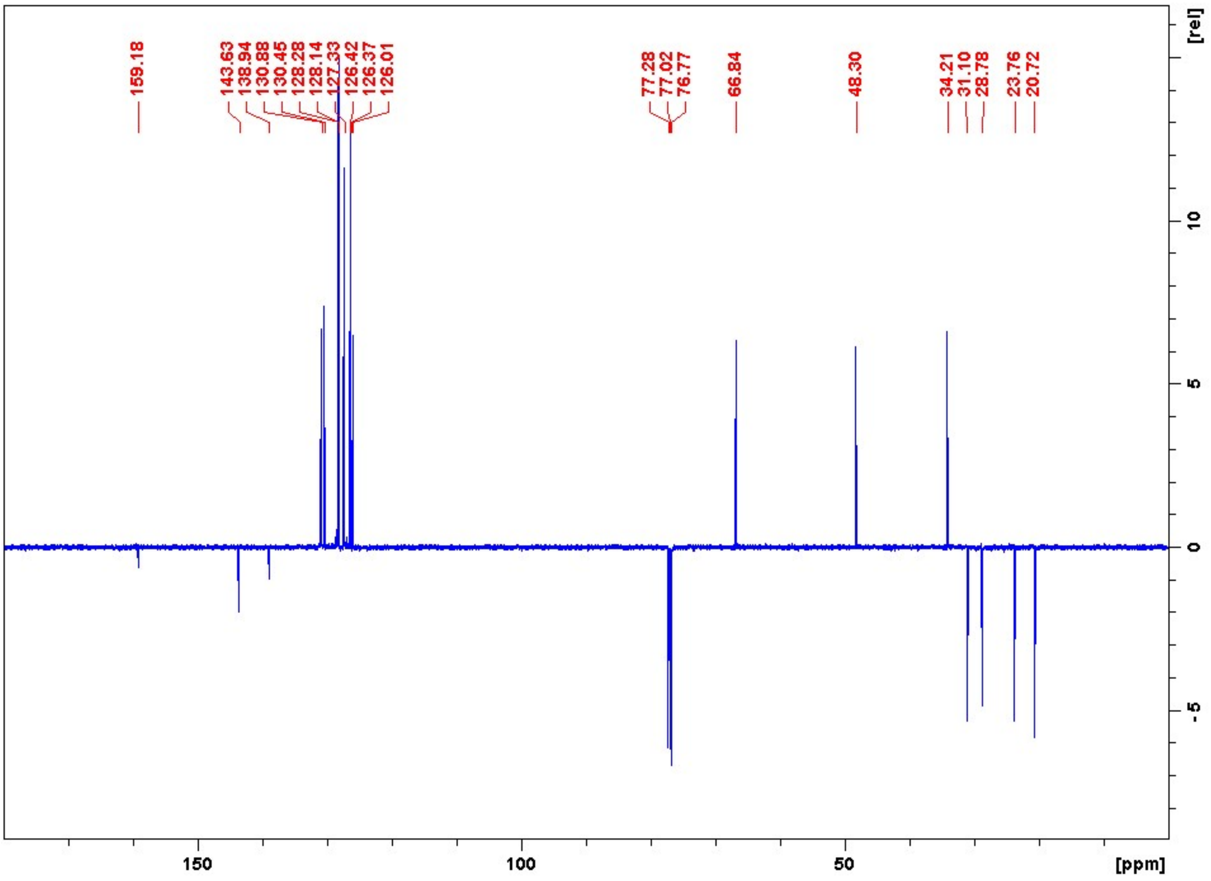

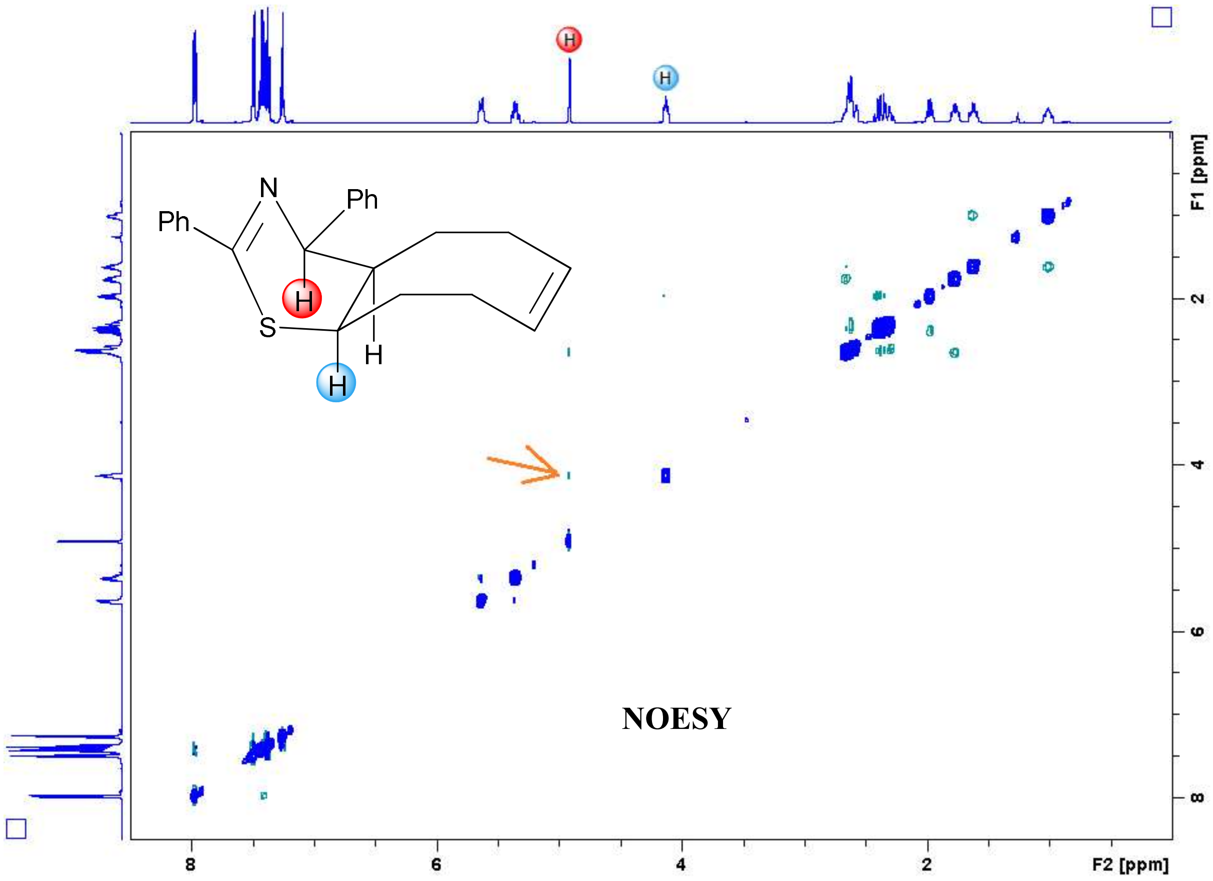
Ph
N
Ph
H
S
H
H
NOESY
F1 [ppm]
F2 [ppm]

(4*S,4a*R**,10a*R**,*Z*)-2,4-diphenyl-4a,5,6,9,10,10a-hexahydro-4*H*-cyclocta[*e*][1,3]thiazine (16ba)**

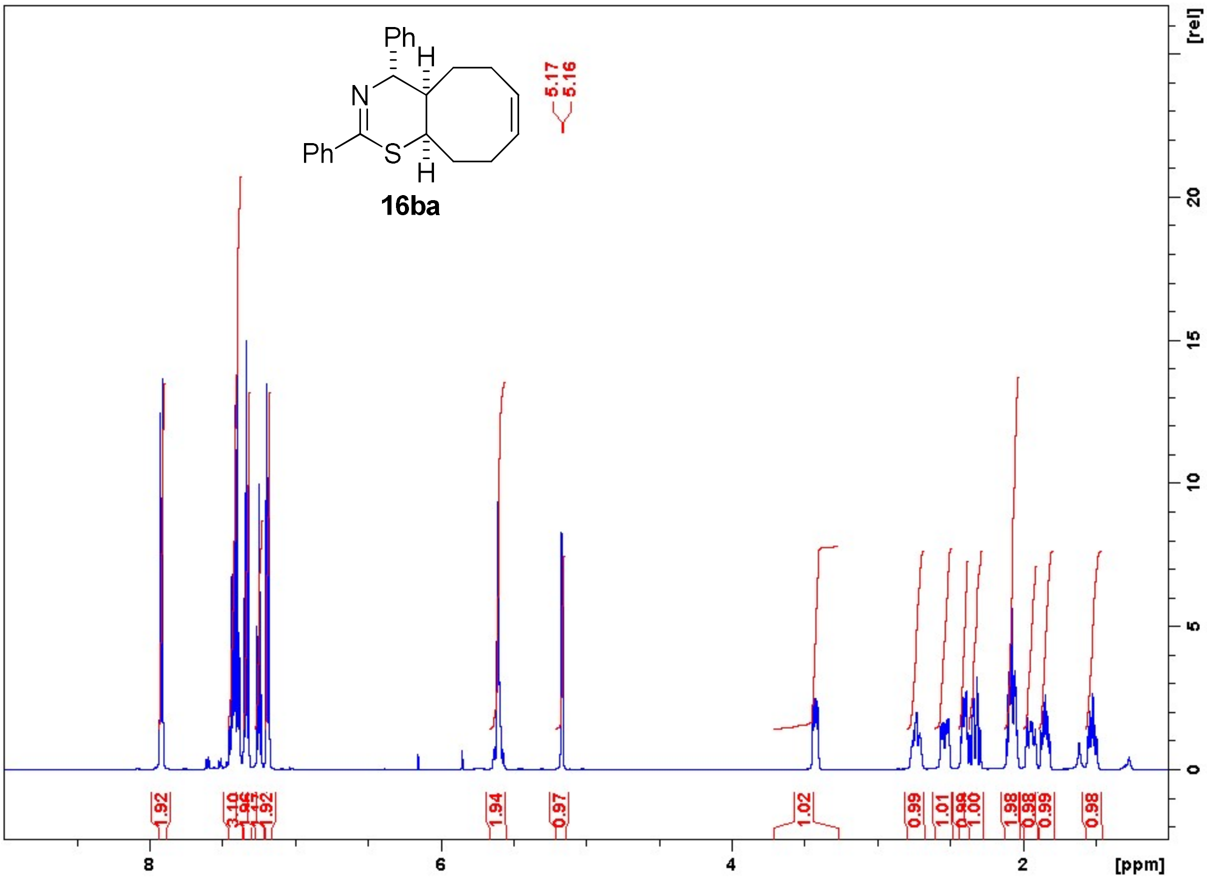

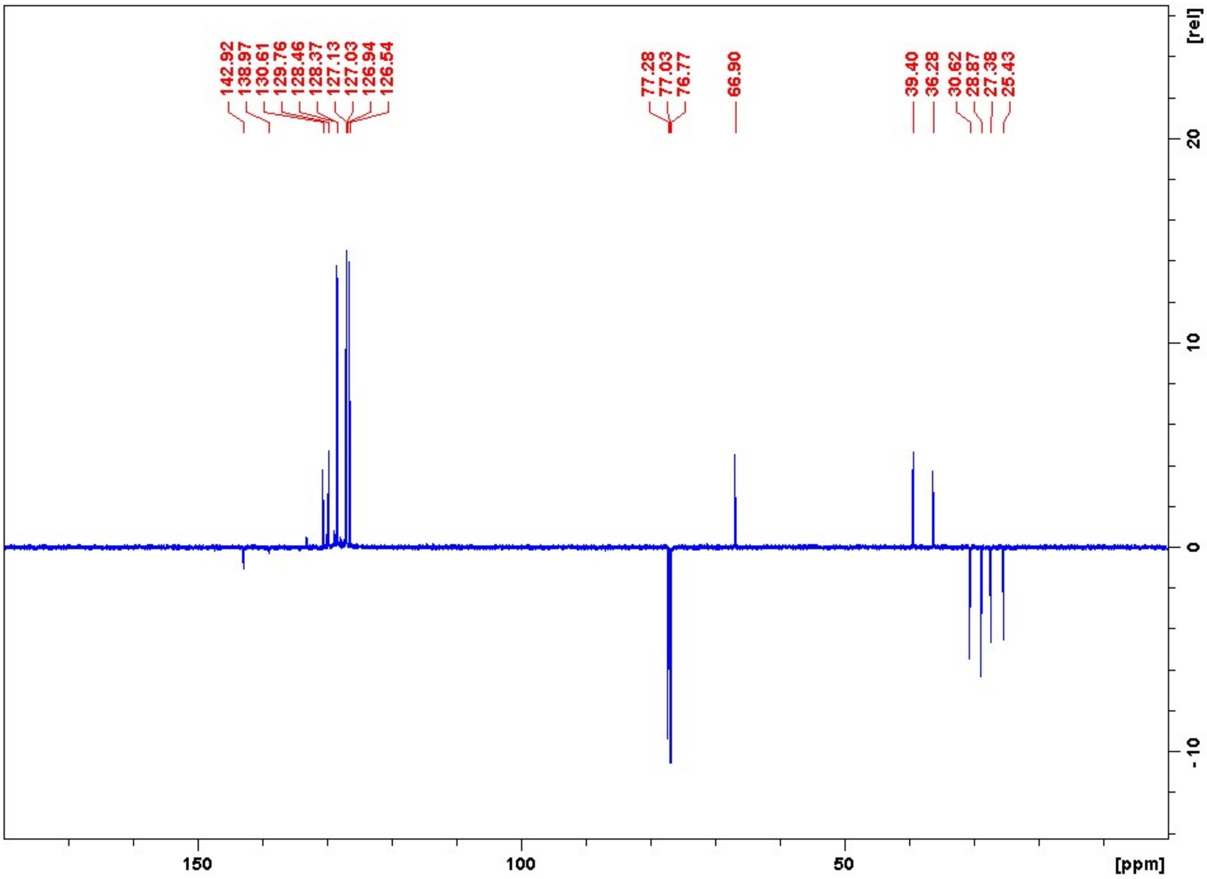

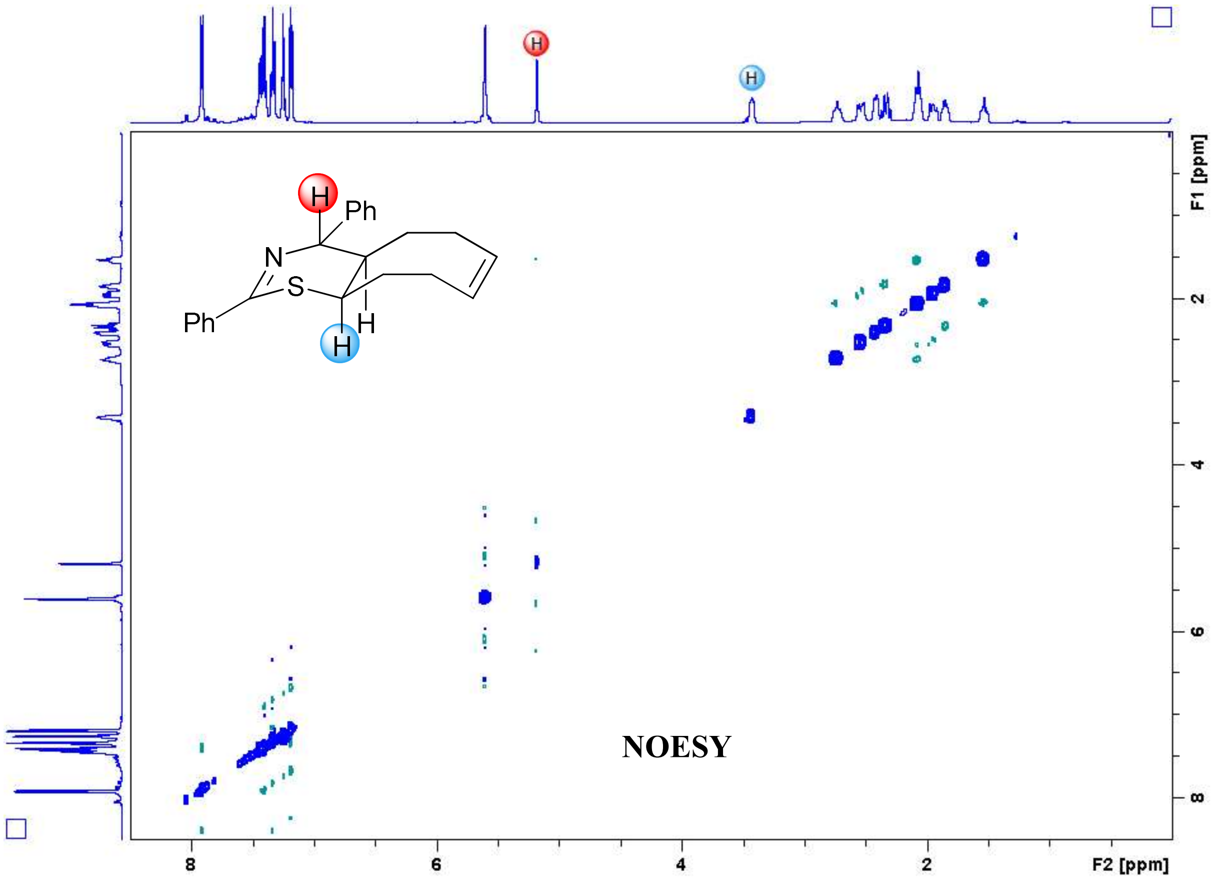
H
Ph
N
S
Ph
H
H
NOESY
F1 [ppm]
F2 [ppm]
8
6
4
2

(4*S,5*R**,8*R**,*Z*)-2,4-diphenyl-1-thia-3-azaspiro[4.7]dodeca-2,6-dien-8-ol (18)**

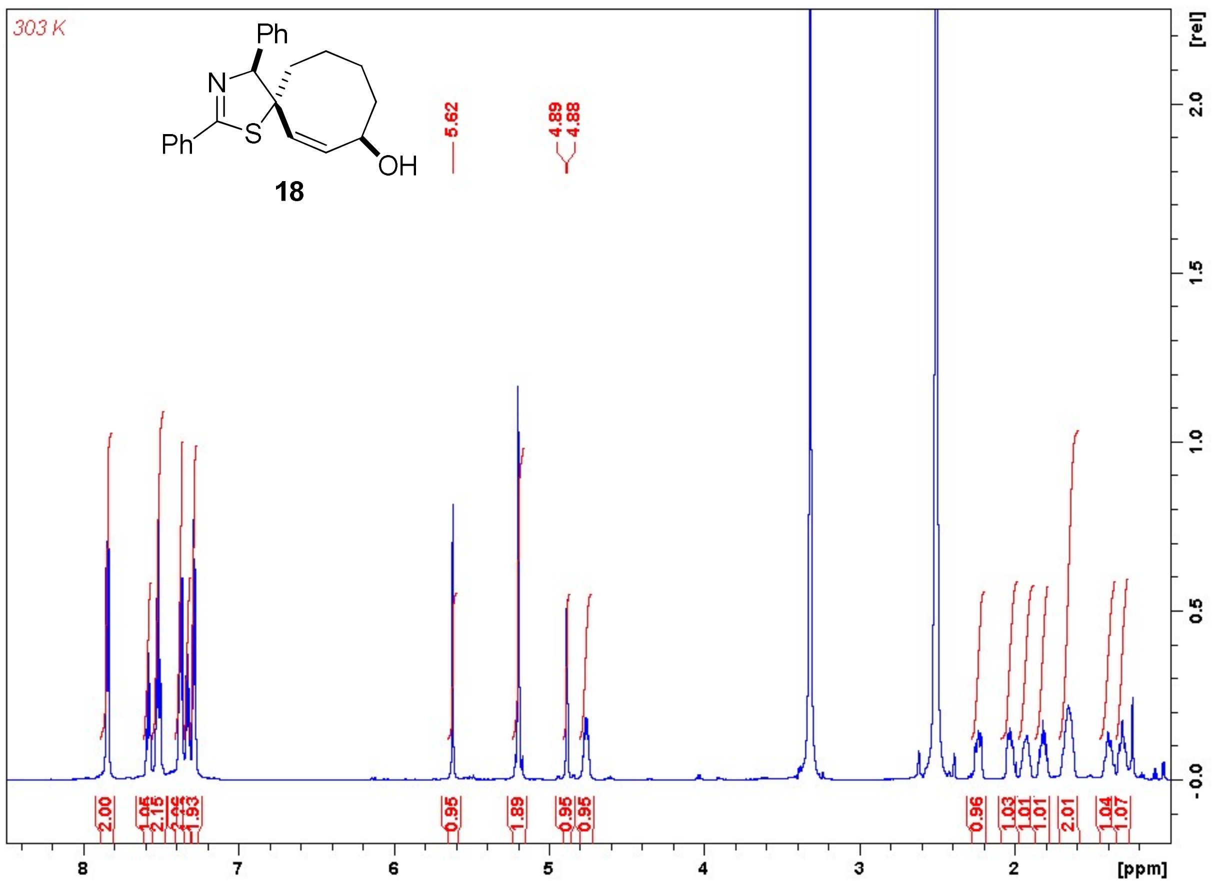

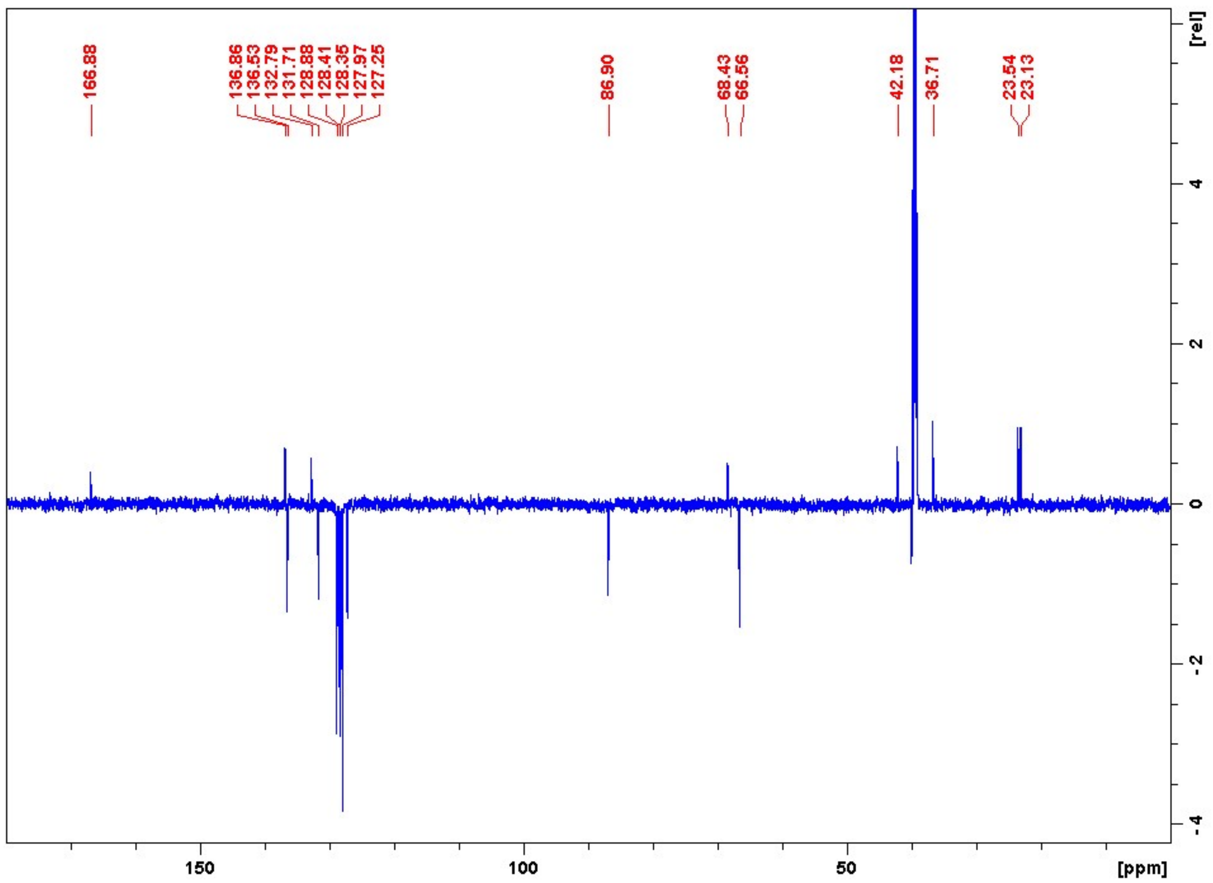

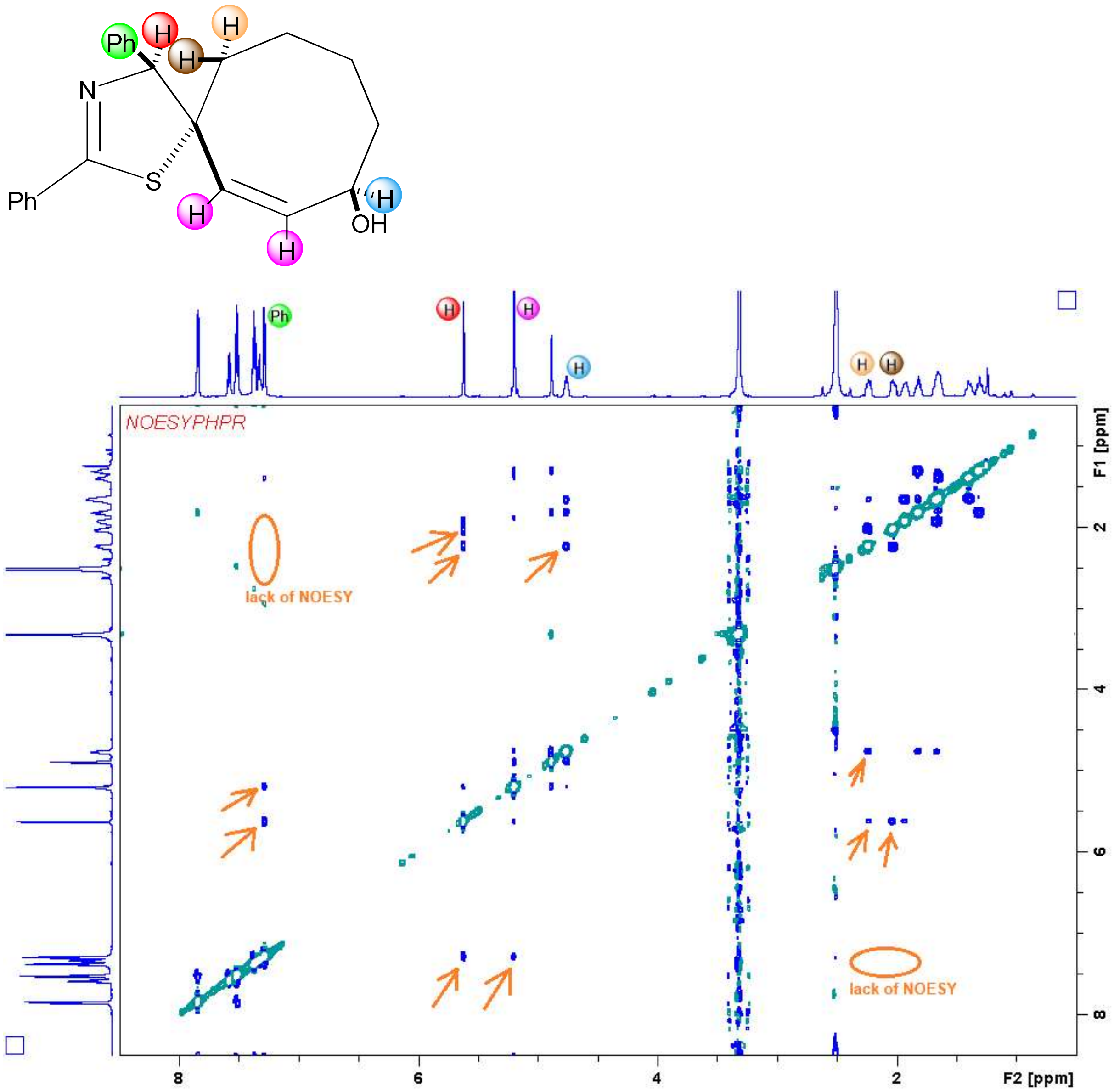
Ph
H
H
H
N
S
Ph
H
H
H
OH
NOESYPHPR
lack of NOESY
lack of NOESY
F1 [ppm]
F2 [ppm]

(4*S,4a*S**,8a*S**)-2-methyl-4-phenyl-4a,5,6,8a-tetrahydro-4*H*-benzo[*e*][1,3]thiazine (20aa)**

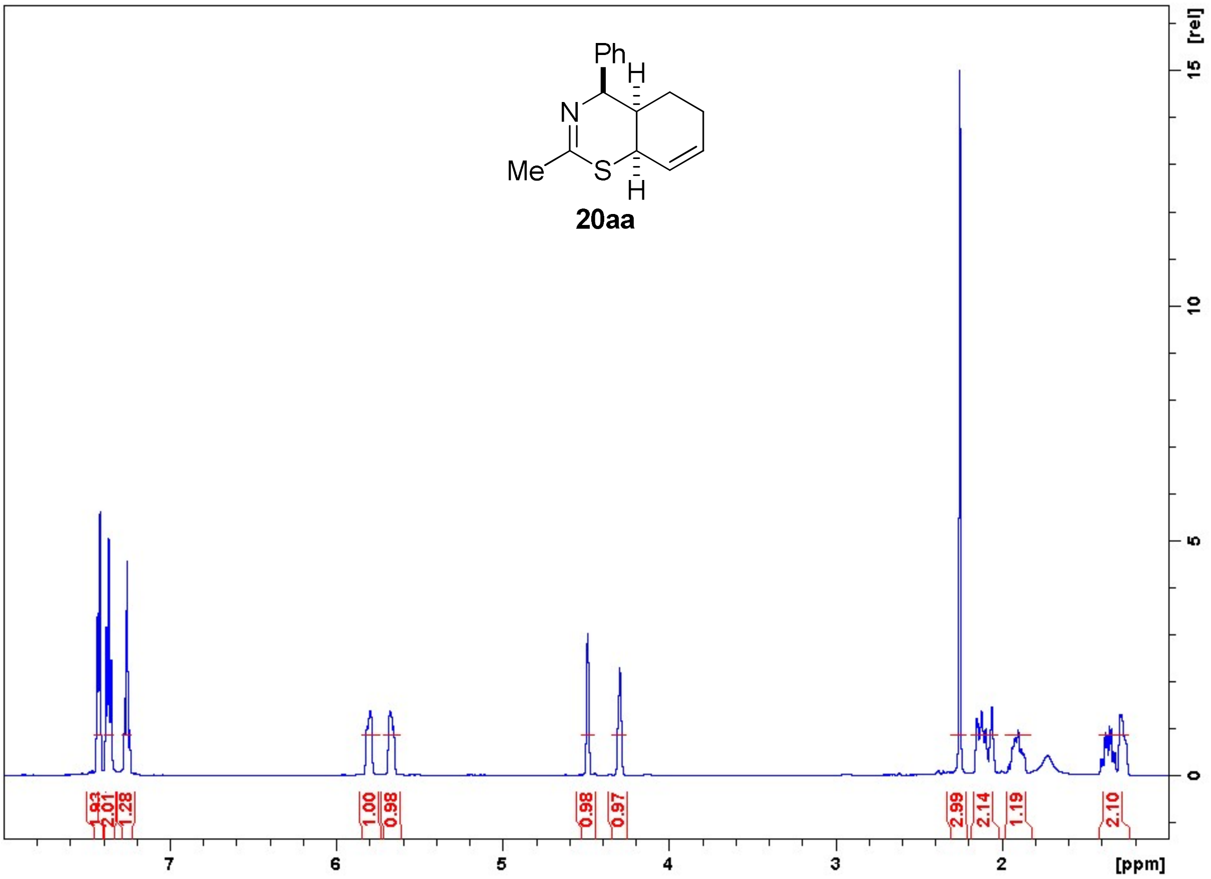

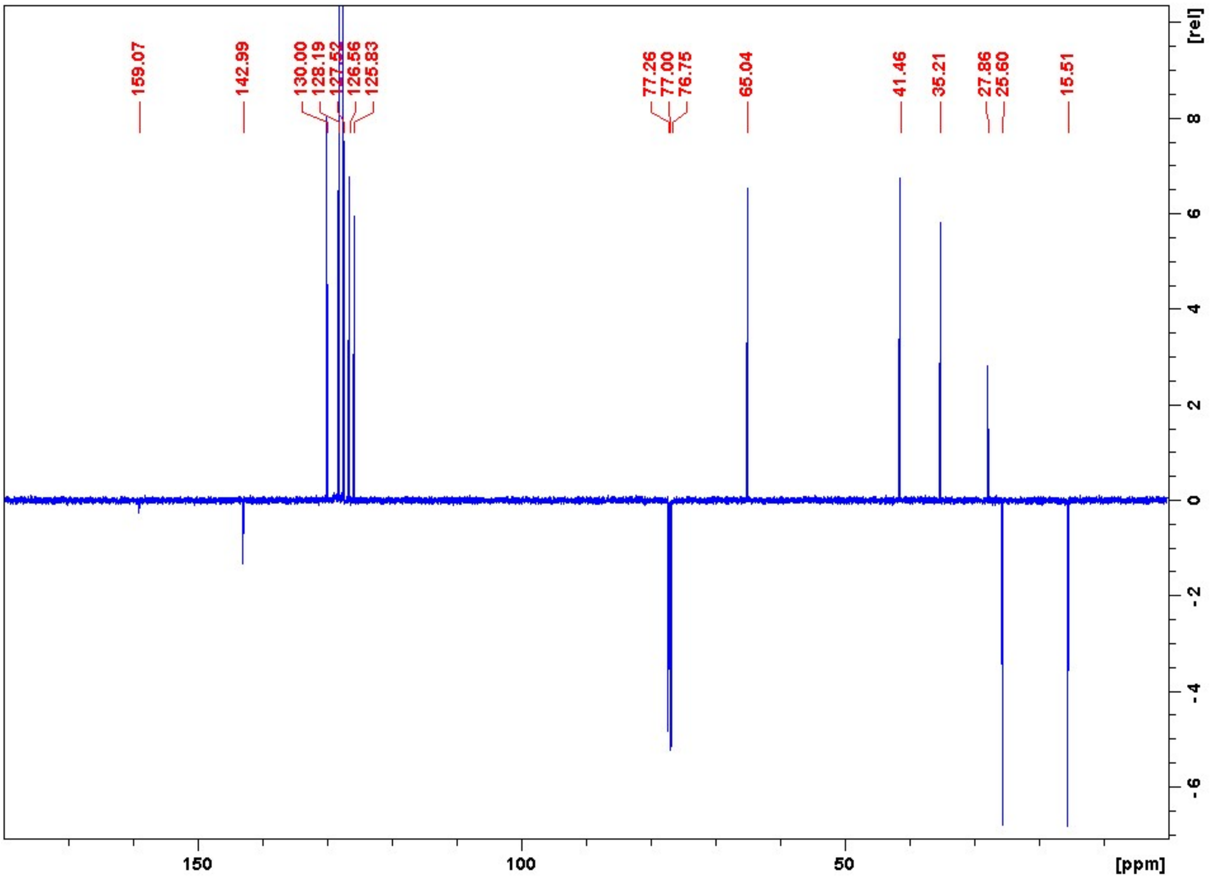

(4*S,4a*S**,8a*S**)-2,4-diphenyl-4a,5,6,8a-tetrahydro-4*H*-benzo[*e*][1,3]thiazine (20ba)**

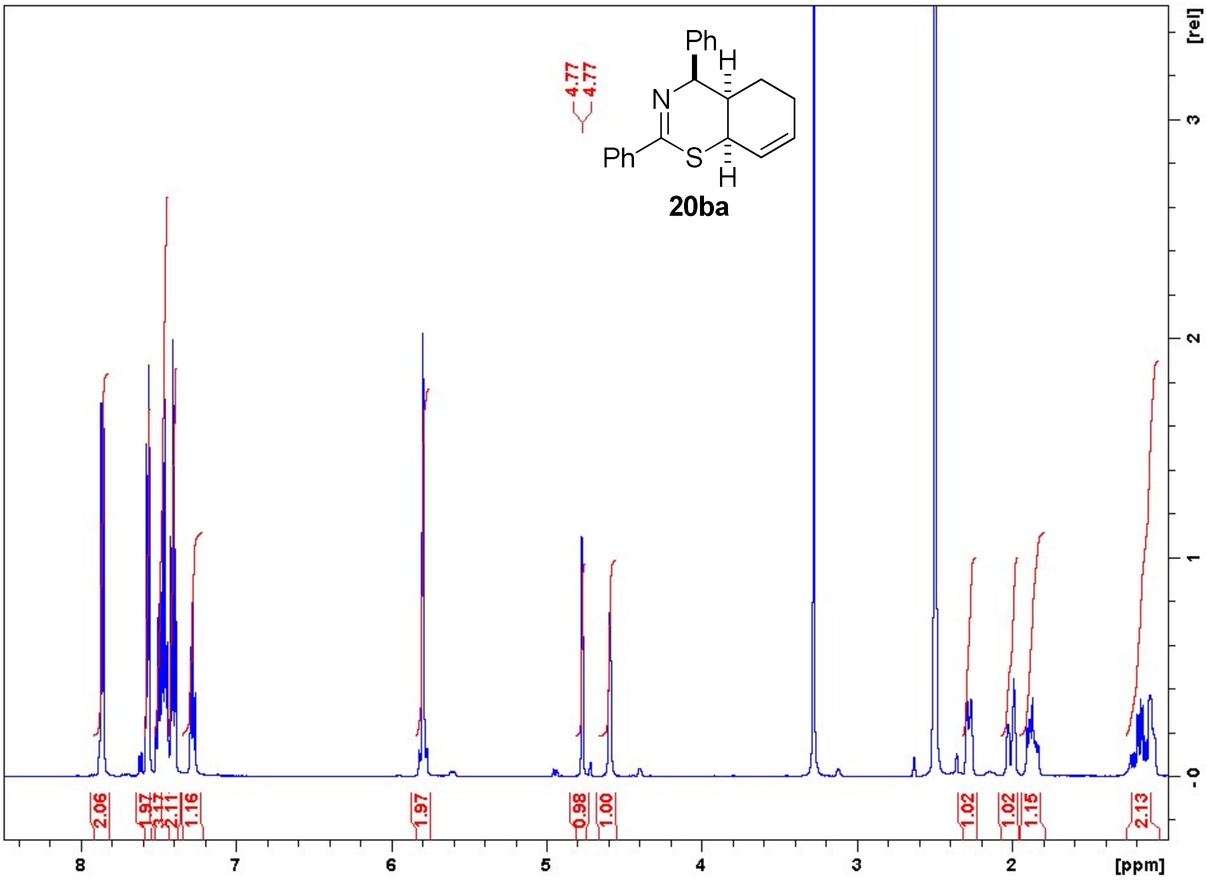

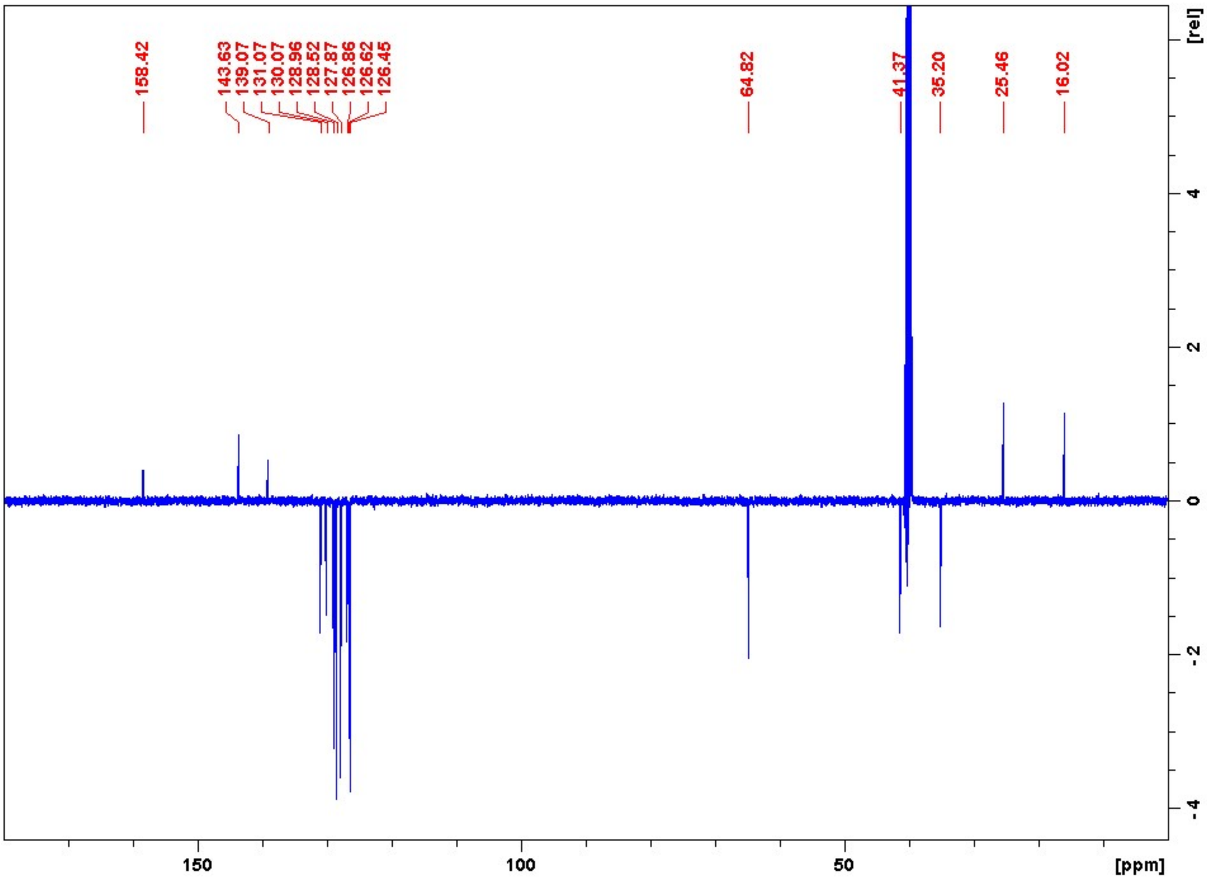

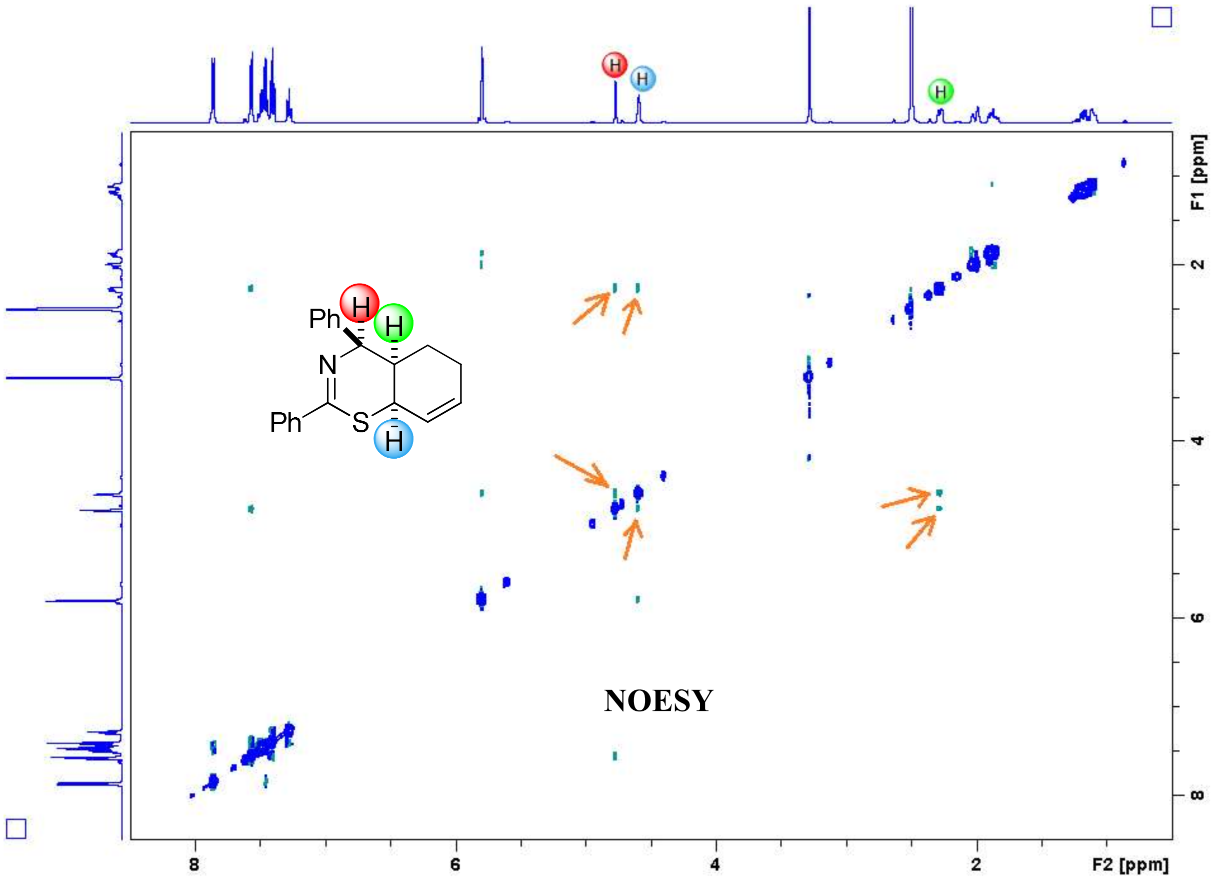

Ph
H
H
N
Ph
S
H
NOESY
8
6
4
2
F2 [ppm]
F1 [ppm]

(2*S,2a*R**,3a*R**,4*R**,7*S**,7a*R**,8*S**)-2-chloro-8-(4-chlorophenyl)-2a-methyl-2,2a,3a,4,7a,8-hexahydro-4,7-methanoazeto[2,1-*b*]benzo[*e*][1,3]thiazin-1(7*H*)-one (21ab)**

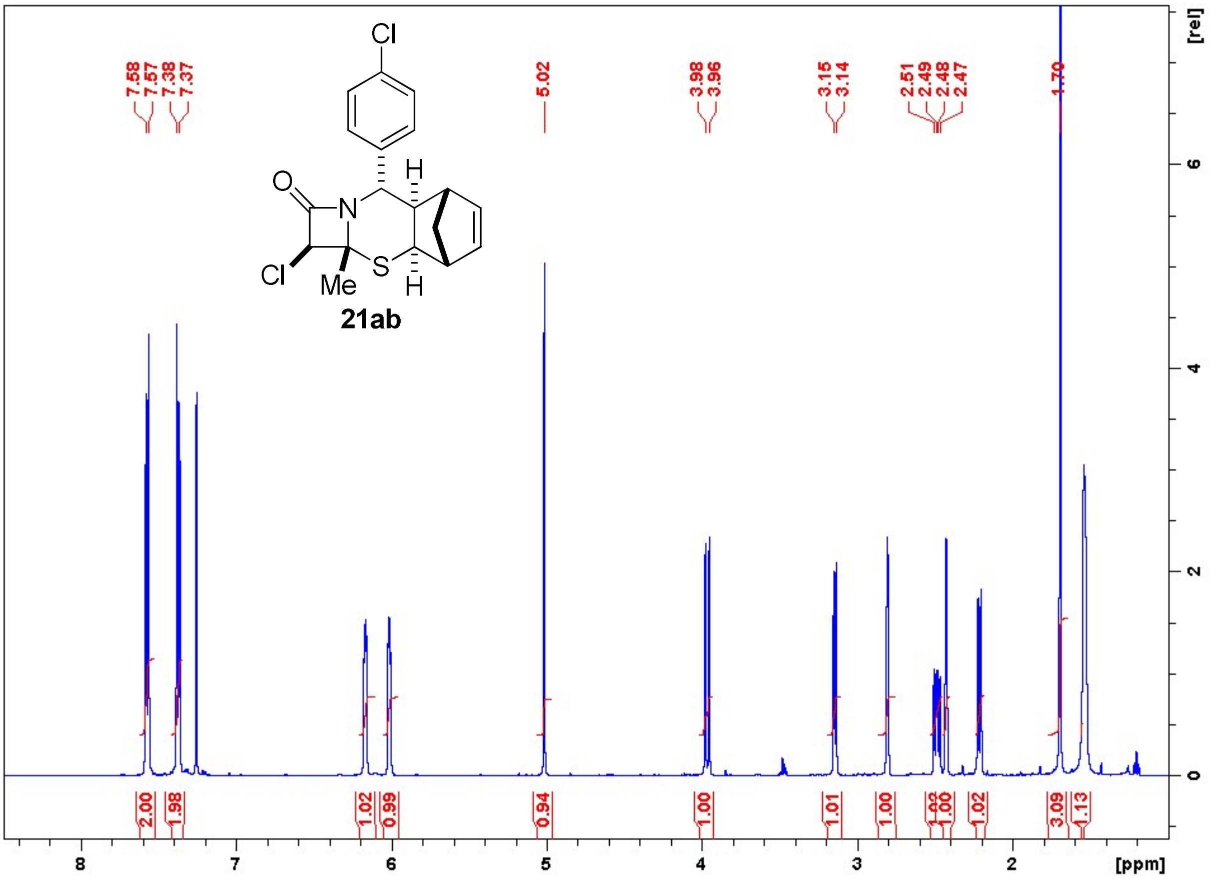

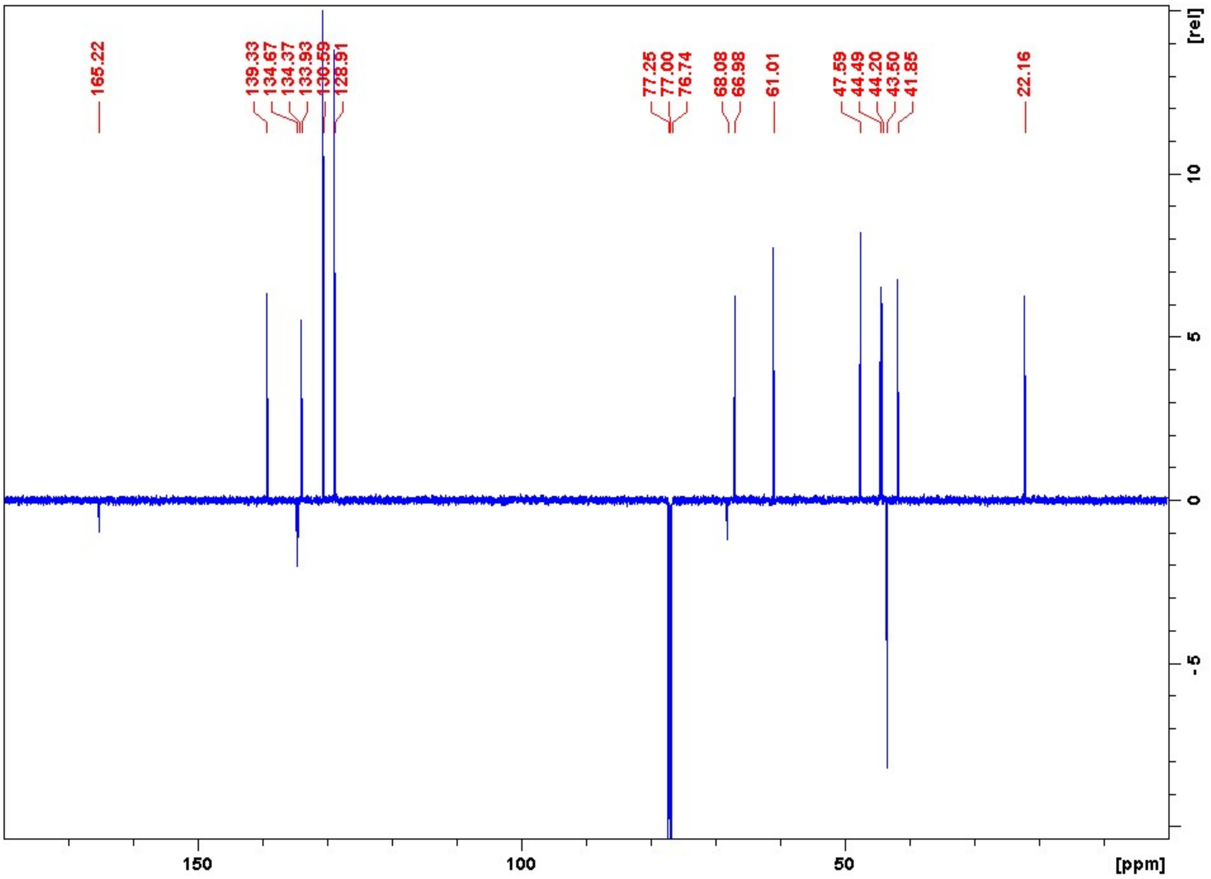

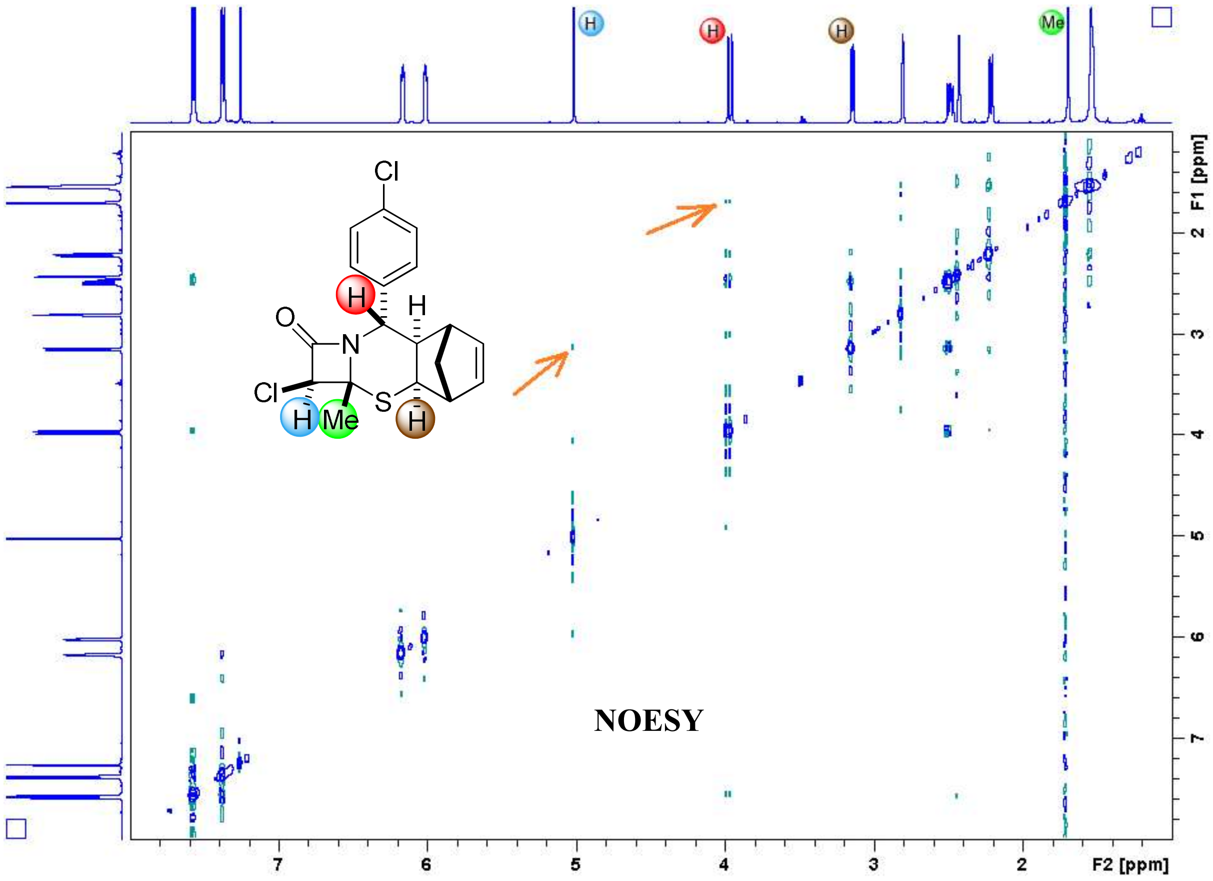

NOESY
F1 [ppm]
F2 [ppm]

(2*R,2a*S**,3a*R**,4*R**,7*S**,7a*R**,8*S**)-2-chloro-2a,8-diphenyl-2,2a,3a,4,7a,8-hexahydro-4,7-methanoazeto[2,1-*b*]benzo[*e*][1,3]thiazin-1(7*H*)-one (21ba)**

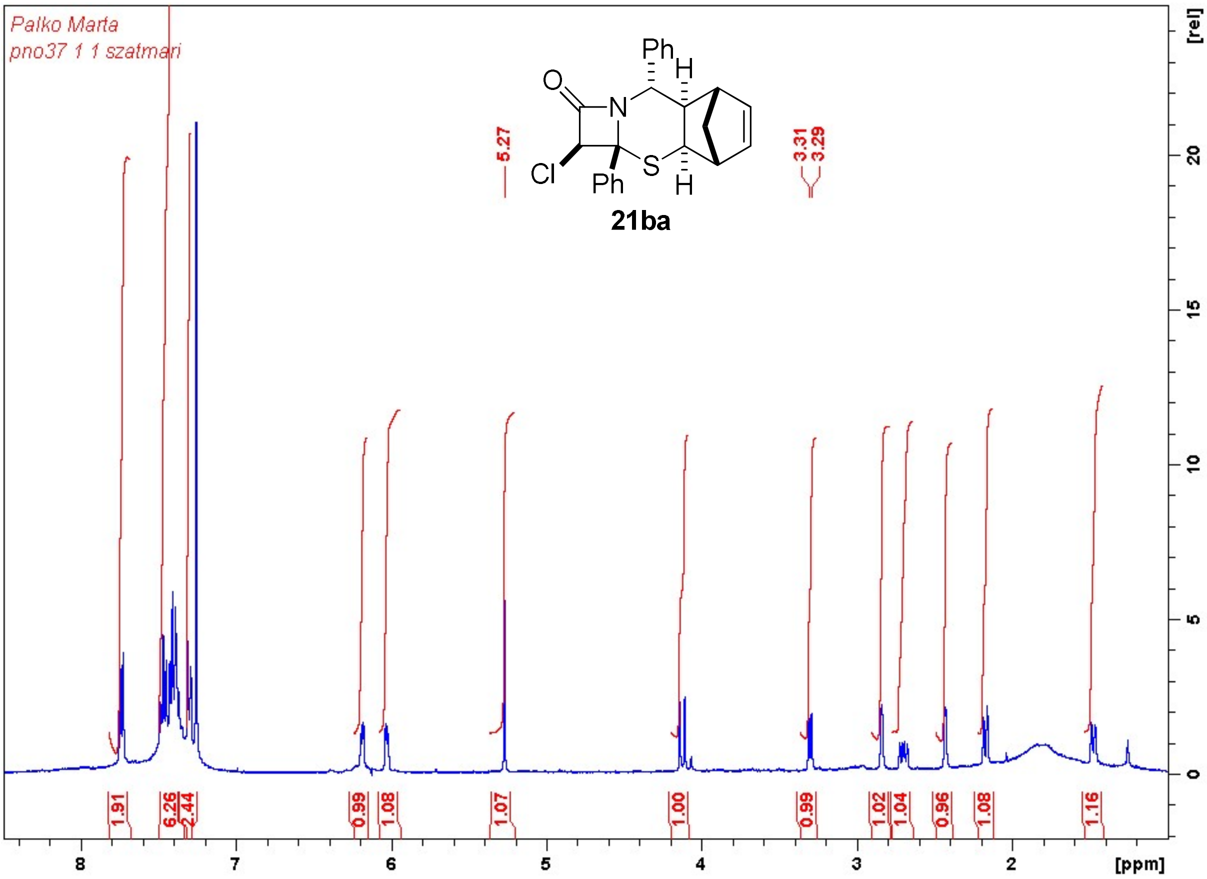

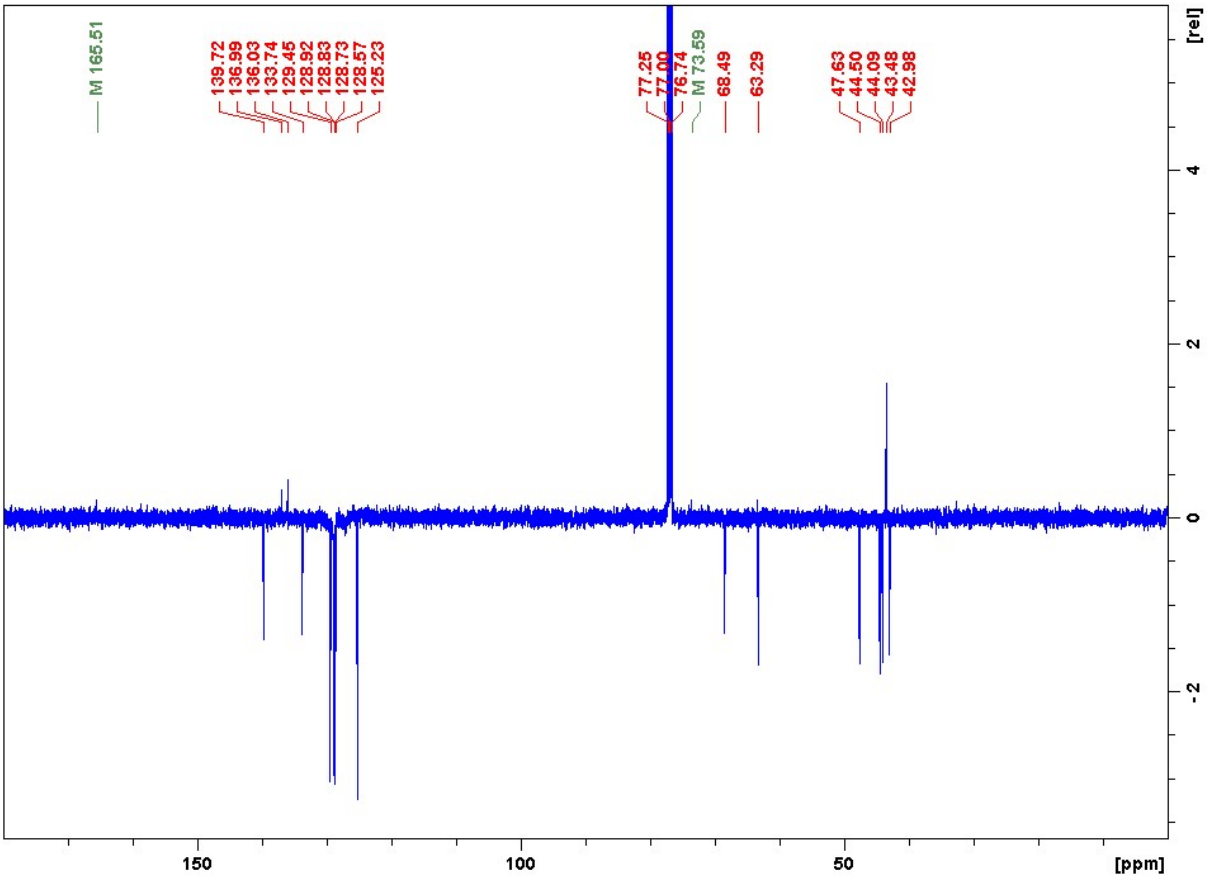

(2*S,2a*R**,3a*R**,4*R**,7*S**,7a*R**,8*S**)-2-chloro-2a-phenyl-8-(4-chlorophenyl)-2,2a,3a,4,7a,8-hexahydro-4,7-methanoazeto[2,1-*b*]benzo[*e*][1,3]thiazin-1(7*H*)-one (21bb)**

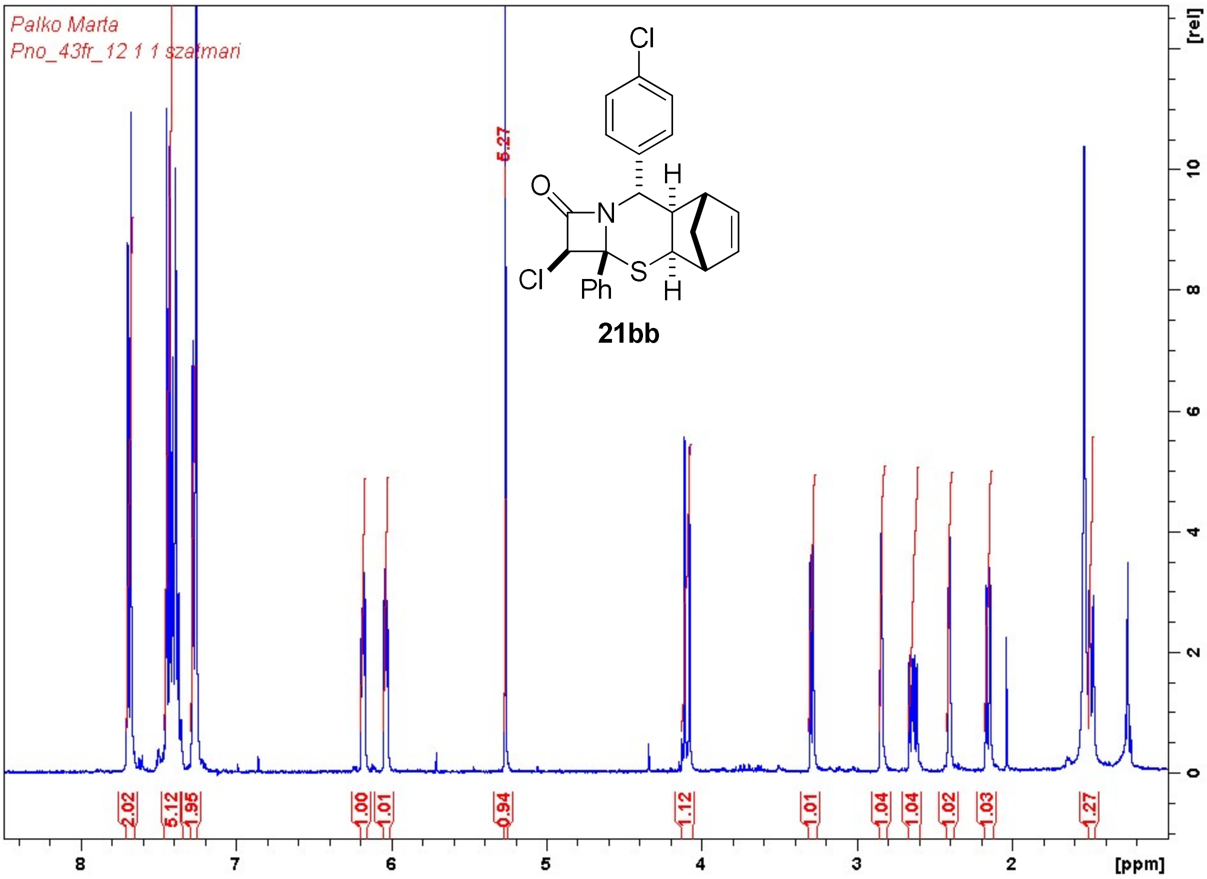

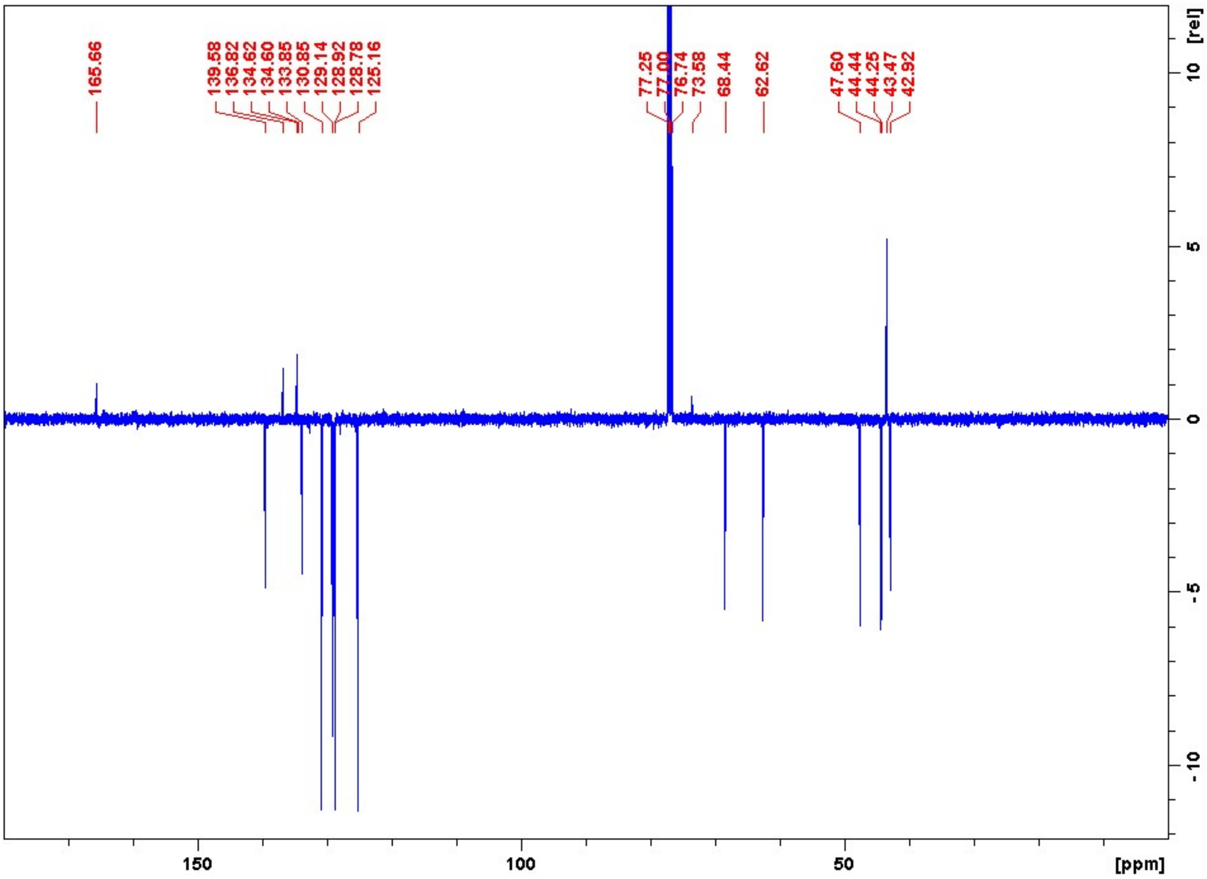

***S*-((1*R**,2*R**,3*R**,4*S**)-3-((*S**)-(2-chloroacetamido)(phenyl)methyl)bicyclo[2.2.1]hept-5-en-2-yl) ethanethioate (22aa)**

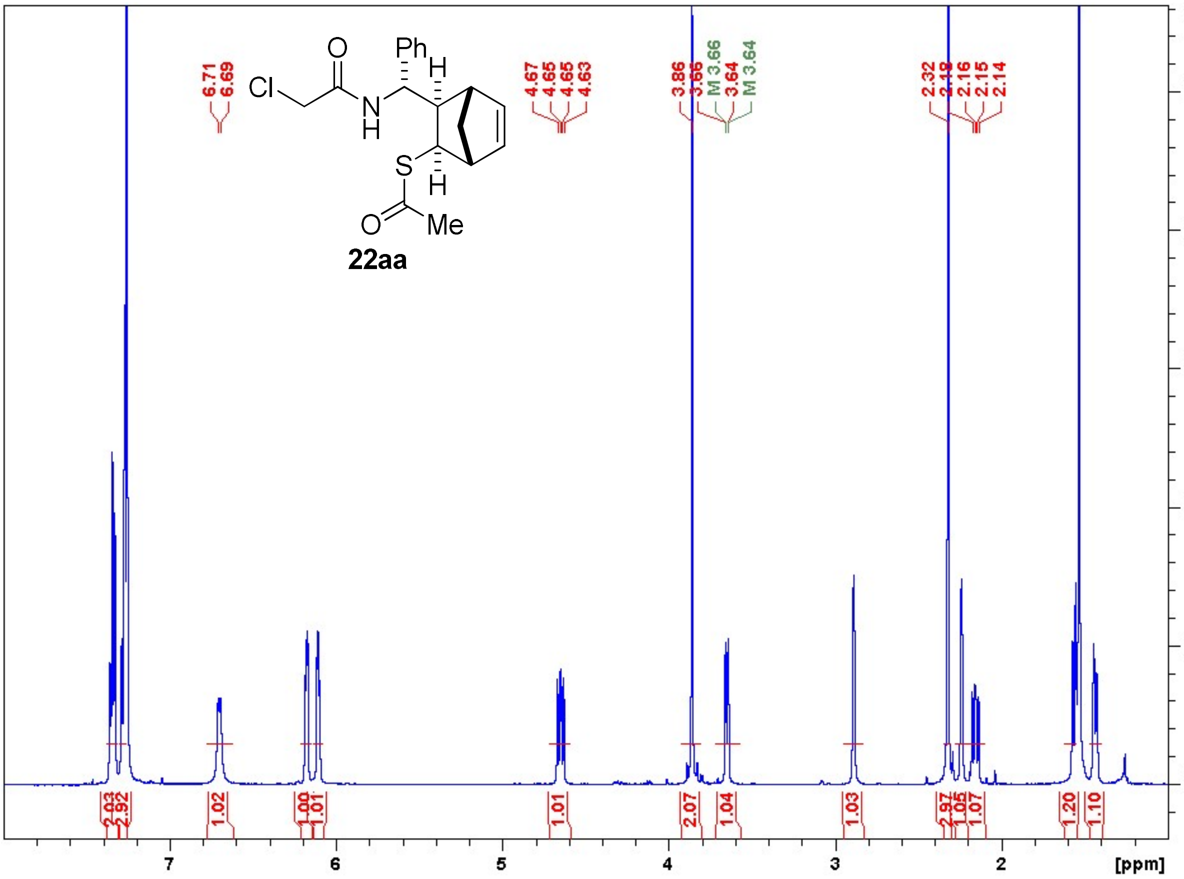

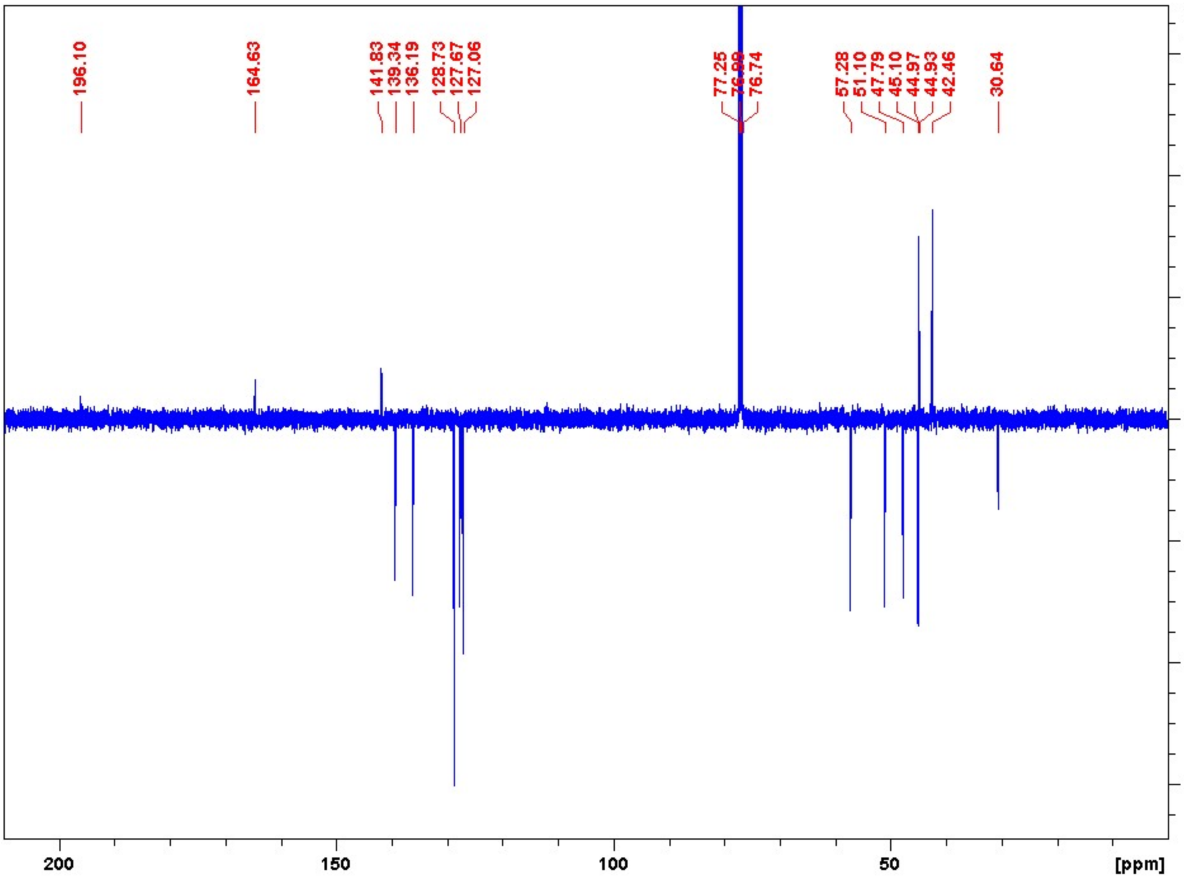

***S*-((1*R**,2*R**,3*R**,4*S**)-3-((*S**)-(2-chloroacetamido)(4-chlorophenyl)methyl)bicyclo[2.2.1]hept-5-en-2-yl) ethanethioate (22ab)**

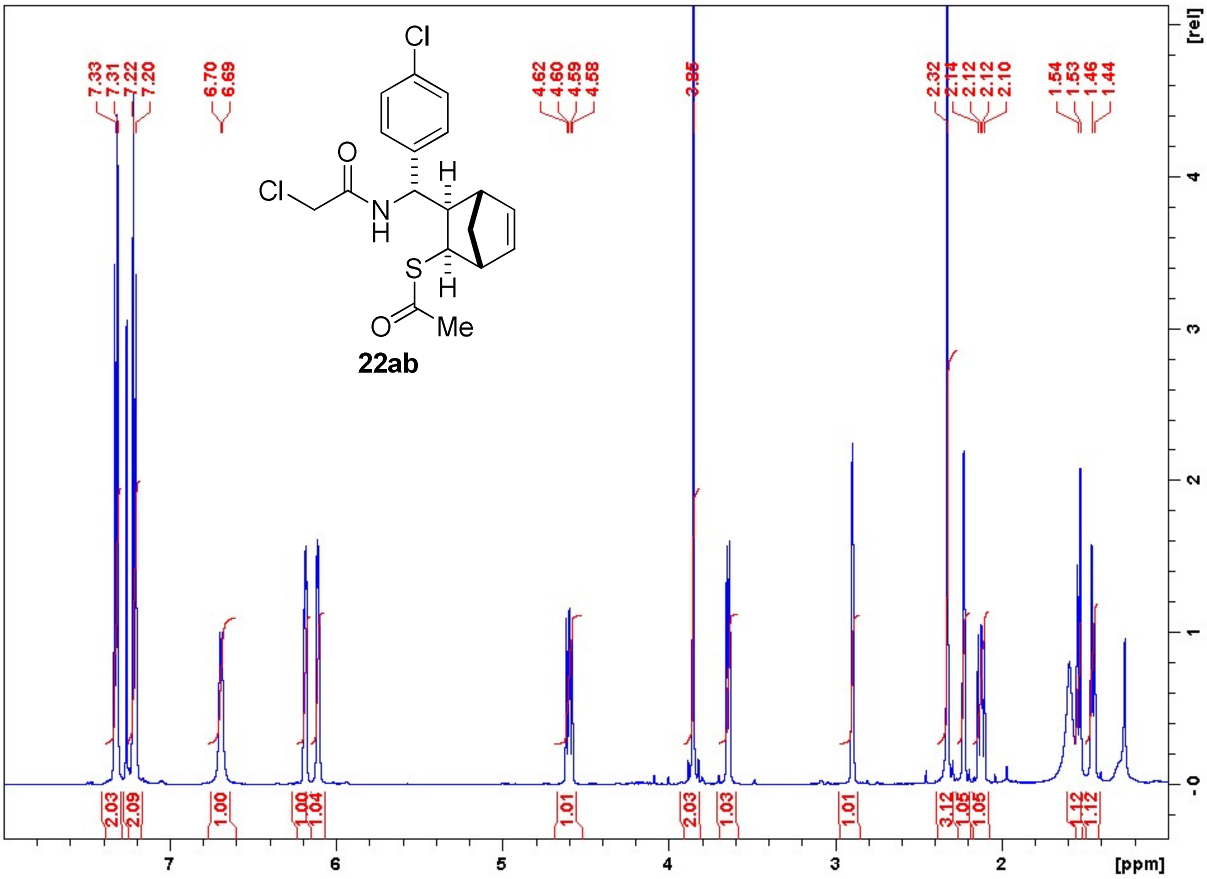

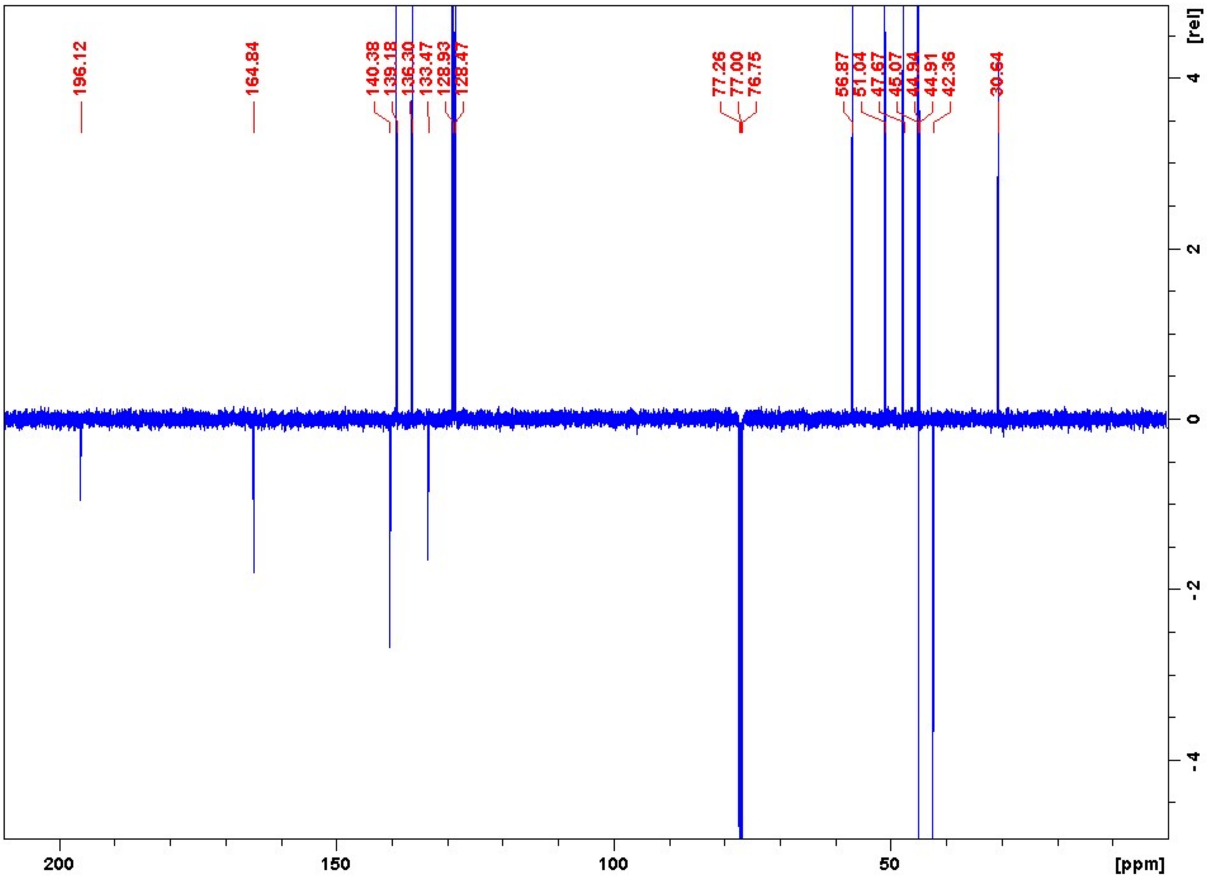

(5*S,5a*R**,6*S**,9*R**,9a*R**)-3,5-diphenyl-4,5,5a,6,9,9a-hexahydro-6,9-methanobenzo[*f*][1,4]thiazepine-2-carboxylic acid methyl ester (23ba)**

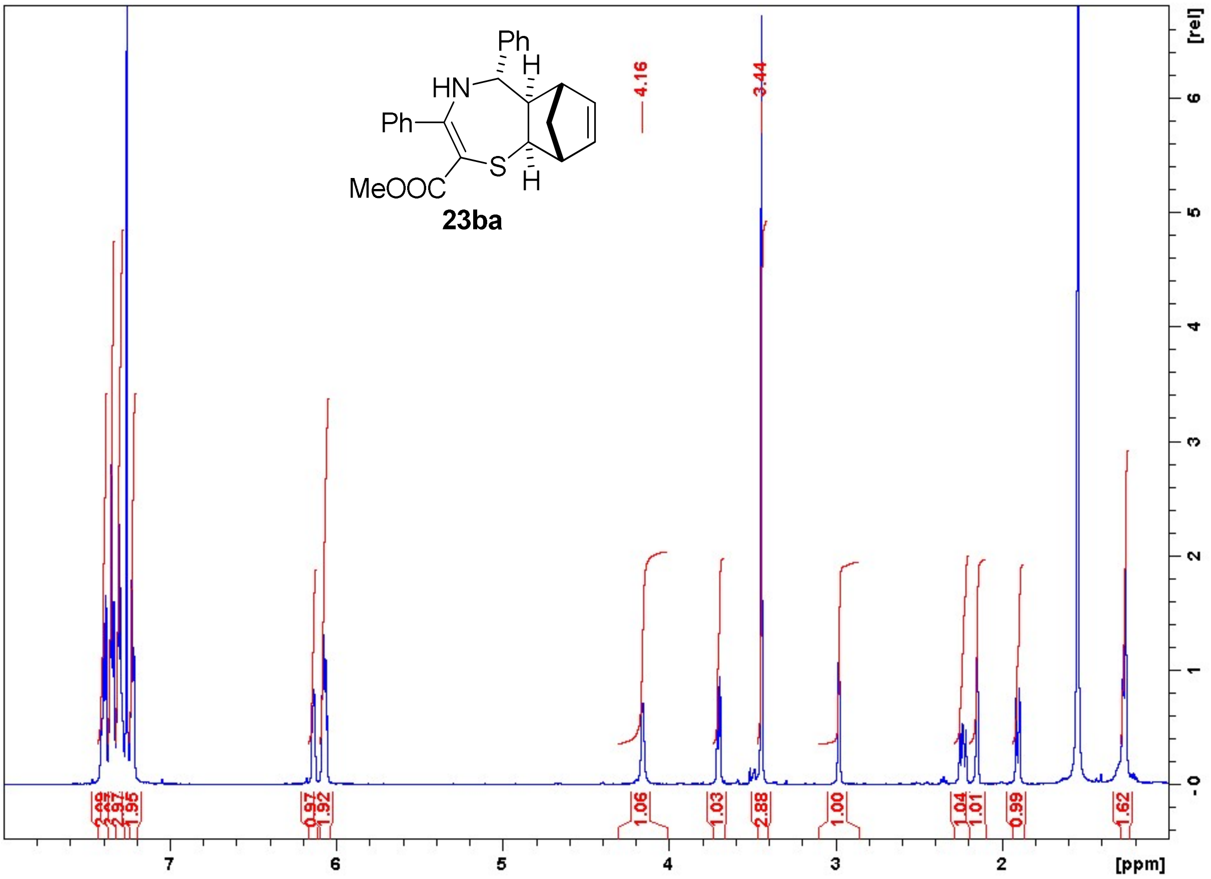

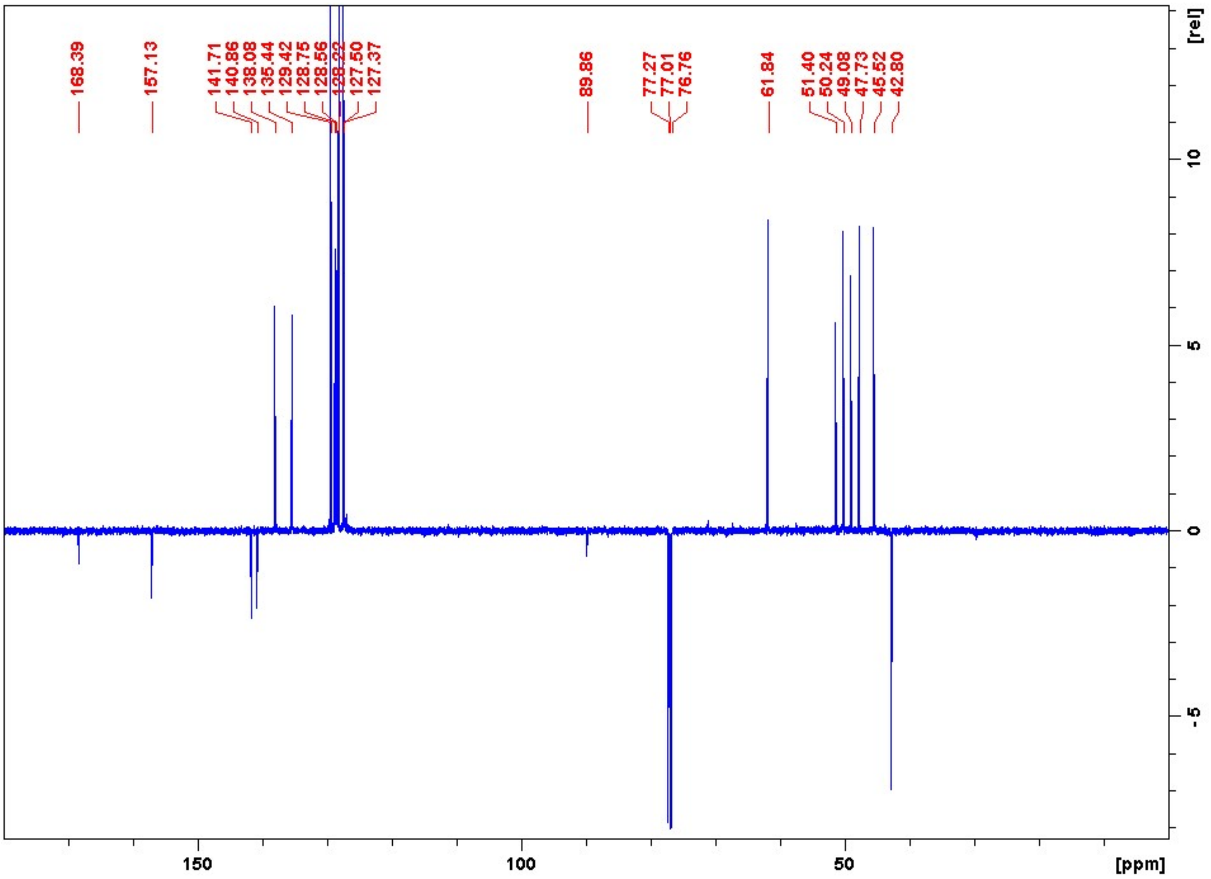

(5*S,5a*R**,6*S**,9*R**,9a*R**)-5-(4-chlorophenyl)-3-phenyl-4,5,5a,6,9,9a-hexahydro-6,9-methanobenzo[*f*][1,4]thiazepine-2-carboxylic acid methyl ester (23bb)**

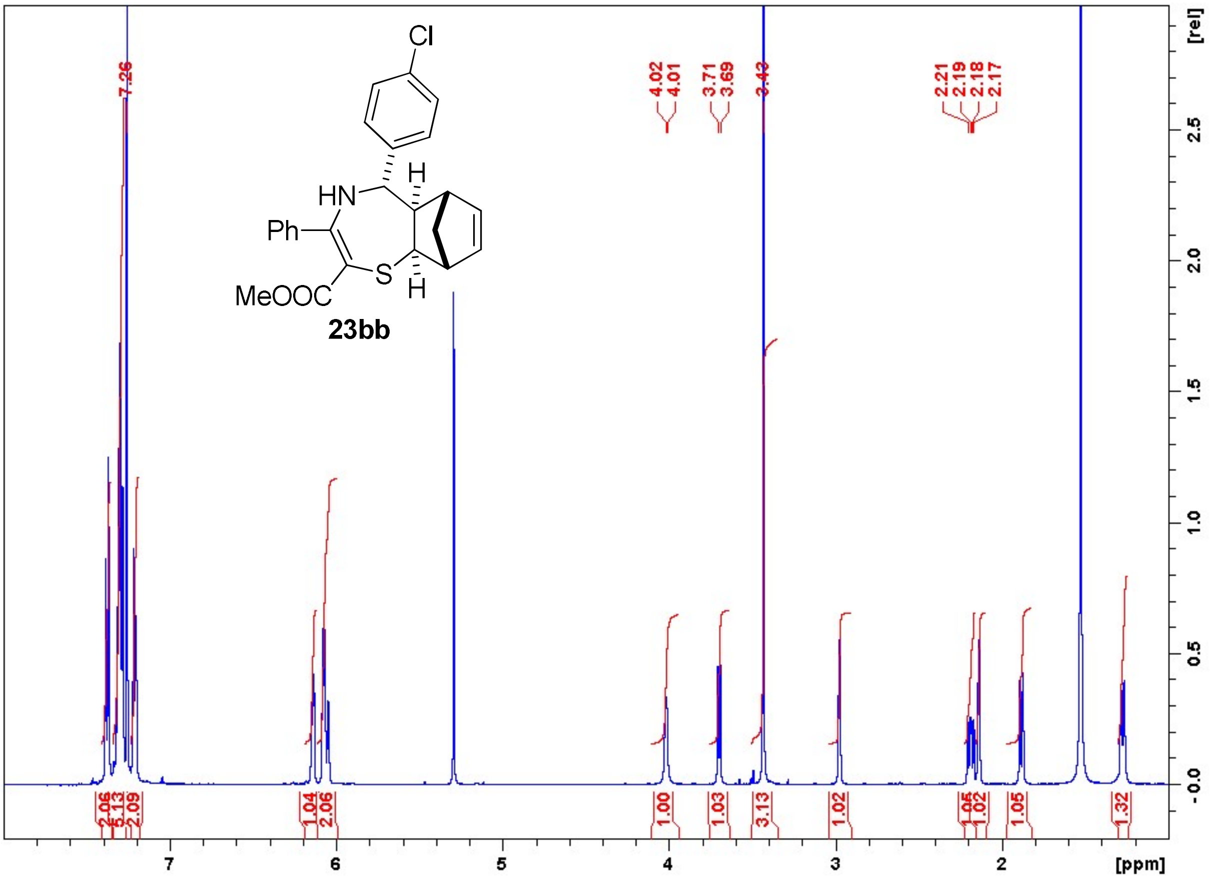

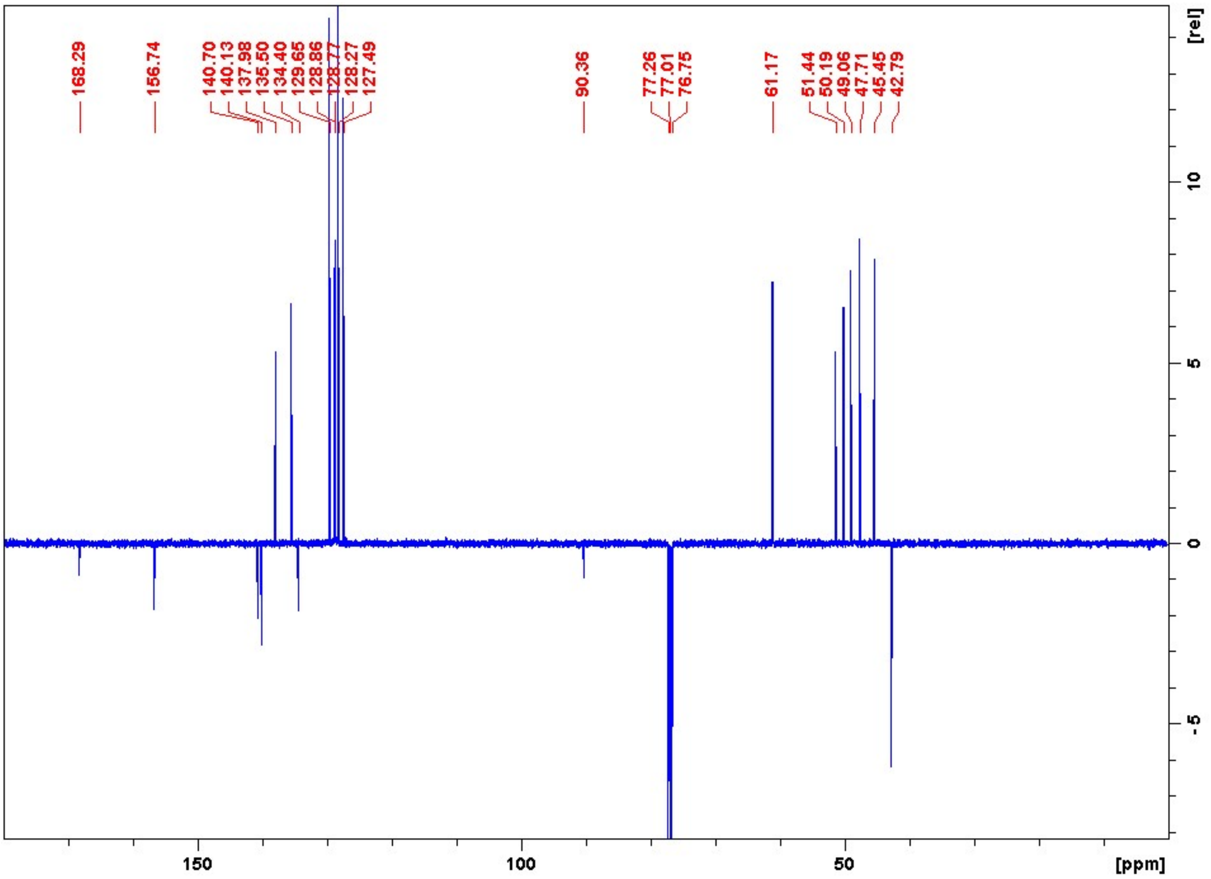

Supplement: Supplementary file 1 [file ijms-26-11543-s001.zip › ijms-3982942-supplementary.pdf]
